# Whole genome duplication through mitotic slippage causes nuclear instability

**DOI:** 10.1038/s44318-026-00912-y

**Published:** 2026-09-19

**Authors:** Simon Gemble, Margot Budzyk, Anthony Simon, Ruxandra Lambuta, Luca Nanni, Nicole Weiss, Audrey Forest, Yekaterina Miroshnikova, Federica Scotto Di Carlo, Véronique Marthiens, Corentin Verdel, Jing Fang, Chantal Desdouets, Sara A Wickström, Giovanni Ciriello, Elisa Oricchio, Geneviève Almouzni, Renata Basto

**Affiliations:** 1 Biology of Centrosomes and Genetic Instability, Institut Curie, Centre National de la Recherche Scientifique (CNRS), Unité Mixte de Recherche 144, Université Paris Sciences et Lettres (PSL Research University), Paris, France; 2 Swiss Institute for Experimental Cancer Research (ISREC), School of Life Sciences, EPFL, Lausanne, Switzerland; 3 Department of Computational Biology, University of Lausanne (UNIL), Lausanne, Switzerland; 4 Chromatin dynamics, Institut Curie, Centre National de la Recherche Scientifique (CNRS) Unité Mixte de Recherche 3664, Université Paris Sciences et Lettres (PSL Research University), University, Sorbonne Université, Paris, France; 5 Max Planck Research Group Biophysical Regulation of Cell State Dynamics, Max Planck Institute for Molecular Biomedicine, 48149 Münster, Germany; 6 Centre de Recherche des Cordeliers, Sorbonne Université, INSERM, Université de Paris, Paris, France; 7 Genomic Instability, Metabolism, Immunity and Liver Tumorigenesis laboratory, Equipe Labellisée LIGUE 2023, Paris, France; 8 Department of Cell and Tissue Dynamics, Max Planck Institute for Molecular Biomedicine, 48149 Münster, Germany; 9 Stem Cells and Metabolism Research Program, Faculty of Medicine, University of Helsinki, 00290 Helsinki, Finland; 10 Present Address: Institute of Molecular Biology, Mainz, Germany

**Keywords:** Cell Cycle, Chromatin, Transcription & Genomics, DNA Replication, Recombination & Repair

## Abstract

Whole-genome duplication (WGD), leading to polyploidy can arise in physiological and pathological contexts. WGD can occur via non-canonical cell cycles such as mitotic slippage, cytokinesis failure or endoreplication. Whether the routes to WGD influence the behaviour of the resulting polyploid cells remains unclear. Here, we compared these routes under both physiological and non-physiological conditions. Remarkably, only mitotic slippage led to widespread nuclear abnormalities defined by highly variable nuclear deformations that we termed nuclear instability. Mechanistically, we found that these nuclei were softer and thus more vulnerable to microtubule-driven deformations. The resulting nuclear instability leads to local nuclear reorganisation and changes in 3D genome organisation. Importantly, we observed similar nuclear instability in megakaryocytes, which are physiological polyploid cells generated by mitotic slippage, providing a molecular mechanism for their atypical nuclear architecture. In striking contrast, nuclear shape was stable in different physiological polyploid cells generated by cytokinesis failure and endoreplication. Overall, our findings highlight that the route towards WGD matters and that mitotic slippage uniquely destabilizes nuclear architecture, with implications for both physiology and disease.

## Synopsis

Whether the route to whole genome duplication (WGD) shapes the behavior of the resulting polyploid cells remains unclear. This work shows that mitotic slippage, but not other causes of WGD, produces irregular nuclear architectures marked by highly variable nuclear deformations, a behavior termed nuclear instability.

- Cells exiting mitosis upon slippage exhibit elevated histone acetylation, which softens the nucleus.
- Softer polyploid nuclei are more susceptible to deformation by the microtubule cytoskeleton.
- Deformation drives local nuclear reorganization and alters chromatin compaction and 3D genome architecture.

## Abstract (web summary)

Cells slipping out of mitosis exhibit elevated histone acetylation that softens the nucleus, making it more susceptible to deformation through the microtubule cytoskeleton.

## Graphical abstract

**Figure:**
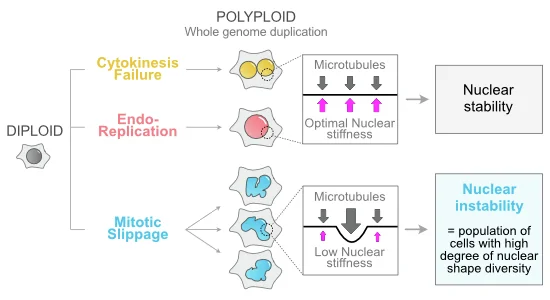

## Introduction

Most animal cells are diploid, carrying two copies of each chromosome (2N). Through a developmentally regulated and reproducible programme, certain cells increase their chromosome number through whole genome duplication (WGD), accumulating multiple sets of each chromosome (>2N)—hereafter called physiological polyploidy (Frawley and Orr-Weaver, 2015). In most cases, the increase in DNA content is related to an increase in metabolic activity, barrier function or even regenerative capacity. For instance, in mammals, megakaryocytes become polyploid (modal ploidy 16N) to increase their metabolic capacity and produce a high number of platelets (Mazzi et al, 2018; Carminita et al, 2024) while salivary gland cells become polyploid (1024N) during *Drosophila* larval development to produce large quantities of glue, which is necessary for *Drosophila* pupation (Frawley and Orr-Weaver, 2015; Zielke et al, 2011; Sigrist and Lehner, 1997). Mammalian hepatocytes also become polyploid (8N) during liver maturation. The role of polyploidy in this context is still debated, but previous studies suggested that polyploid hepatocytes may have higher metabolic and regenerative capacities (Davoli and de Lange, 2011; Gentric and Desdouets, 2015). In contrast, when WGD occurs in cells that should not become polyploid—hereafter called unscheduled polyploidy—it leads to genetic and chromosomal instability (Nano et al, 2019; Gemble et al, 2022; Kirsch-Volders et al, 2025; Ganem et al, 2007; Goupil et al, 2020; López et al, 2020; Prasad et al, 2022; Kuznetsova et al, 2015). Furthermore, the resulting tetraploidy (4N)—that results from a single WGD—is a common feature of human tumours. Indeed, about 30% of tumours are tetraploid, reaching up to 70% in some specific cases like non-small cell lung carcinomas (López et al, 2020; Zack et al, 2013; Bielski et al, 2018; Blakely et al, 2017).

**Figure 1:**
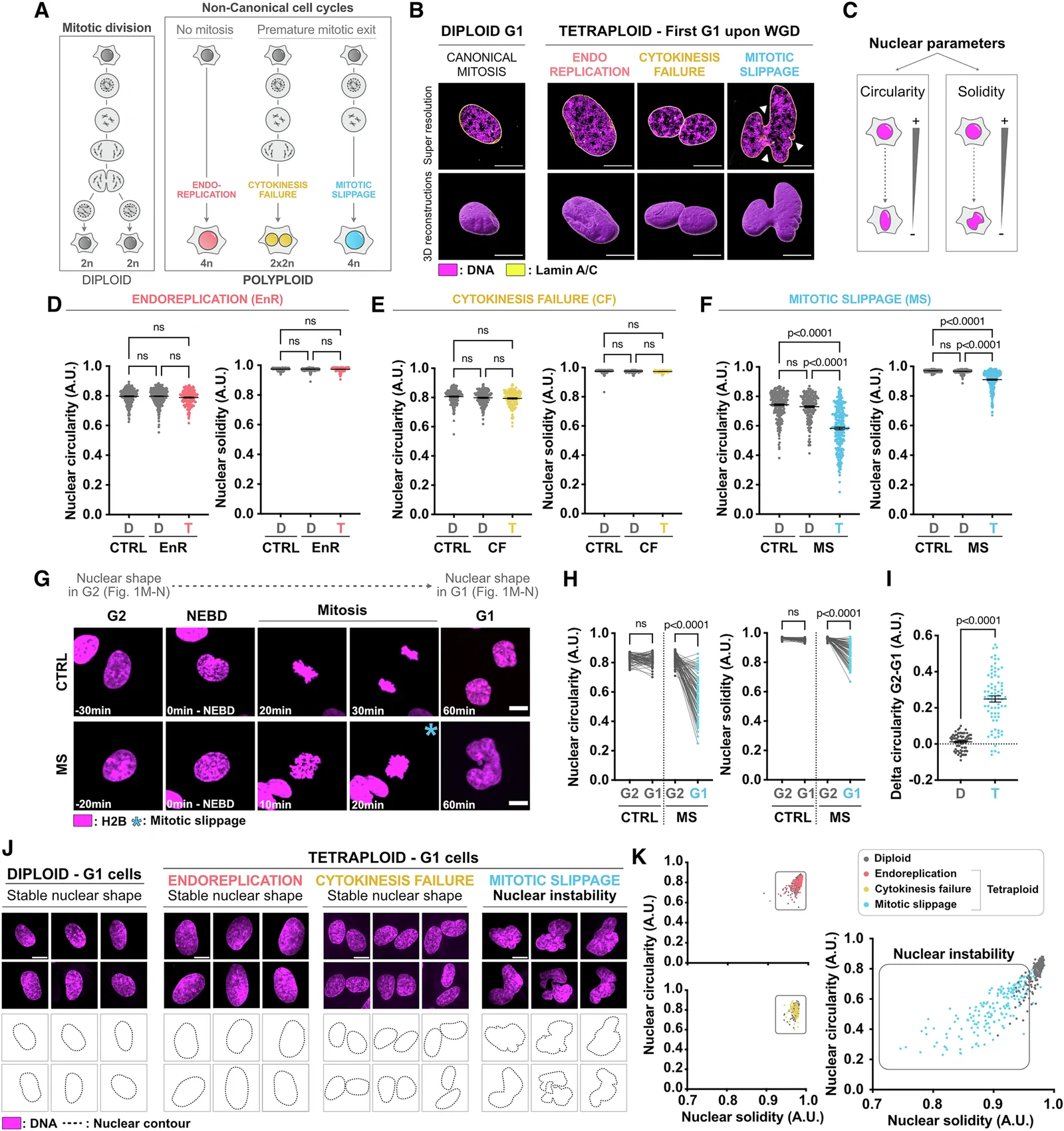
Unscheduled WGD induced through MS generates cells with nuclear instability. (**A**) Schematic representations of mitotic division (left panel) and of the different means employed to generate tetraploid cells (right panel). (**B**) Structural illumination microscopy (SIM) representative images (top) and their corresponding 3D reconstructions (bottom) showing nuclear shape in G1 RPE-1 cells upon canonical mitotic division, MS, CF or EnR. Lamin A/C in yellow, DNA in magenta. The white arrows indicate nuclear invaginations. (**C**) Schematic illustration of the circularity and solidity indices. (**D**–**F**) Graphs showing the nuclear circularity and solidity indices in RPE-1 G1 cells upon (**D**) EnR (in red), (**E**) CF (in yellow) and (**F**) MS (in blue). Mean ± SEM, >150 G1 cells analysed per condition, three independent experiments. (**G**) Stills from time-lapse imaging of RPE-1 cells stably expressing mCherry-H2B (in magenta) performing mitotic division (upper panel) or MS (lower panel). The blue asterisk indicates when the mitotic slippage occurred. (**H**) Graph presenting the nuclear circularity and solidity measured at the single cell level in the same cells in G2 and in G1 upon mitotic division (in grey) or MS (in blue). >80 cells analysed per condition, three independent experiments. (**I**) Graph showing the delta circularity (defined as the difference between nuclear circularity in G2 vs in G1) upon mitotic division (in grey) or MS (in blue) in RPE-1 cells. Mean ± SEM, >75 cells analysed per condition, three independent experiments. (**J**) Representative images showing G1 RPE-1 nuclei upon mitotic division, MS, CF and EnR (upper panel). DNA in magenta. The nuclear contour is indicated (black dotted line, lower panel). (**K**) Graph showing the nuclear circularity and solidity index from (**D**–**F**) in diploid (in grey) and in tetraploid G1 RPE-1 cells generated by EnR (in red), CF (in yellow) and MS (in blue). Scale bars: 10 µm. WGD whole genome duplication, D diploid, T tetraploid, MS mitotic slippage, CF cytokinesis failure, EnR endoreplication. NEBD nuclear envelope breakdown. AU arbitrary unit. (**D**–**F**) ANOVA test (one-sided). (**H**,** I**) *t*-test (two-sided). ns not significant. Source data are available online for this figure.

The canonical cell cycle typical of somatic cells relies on coupling genome duplication with mitotic cell division to generate two diploid daughter cells (Basu et al, 2022). Polyploid cells can be generated by alternative cell cycles - hereafter called non-canonical cell cycles. These cell cycles rely on incomplete or absent mitosis, uncoupling the duplication of the genome from cell division, leading thus to WGD (Gemble and Basto, 2020). Cells can exit mitosis before or after chromosome segregation through mitotic slippage (MS) or cytokinesis failure (CF), respectively (Fig. 1A). Otherwise, cells can alternate S-phase with G-phase using a cell cycle called endoreplication (EnR) (Fig. 1A). MS and EnR generate polyploid cells with a single nucleus, while CF generates binucleated polyploid cells (Penington and Streatfield, 1975; Guidotti et al, 2003; Sigrist and Lehner, 1997). However, the functional consequences of these non-canonical cell cycles remain poorly understood. Indeed, while MS, CF and EnR have been proposed to take place in physiological and pathological contexts (Frawley and Orr-Weaver, 2015; Davoli and de Lange, 2011; Gemble and Basto, 2020; Ganem et al, 2007; Zeng et al, 2023), it is still not clear whether different means to reach WGD lead to different outcomes.

Here, by combining comparative analyses integrating physiological and unscheduled models of WGD, we found that MS, unlike CF and EnR, generates polyploid cells with irregular nuclear architecture. Mechanistically, we identified that MS leads to mitotic exit with high levels of histone 3 phosphorylation on serine 10 (H3S10). This impairs chromatin compaction, compromising nuclear stiffness and allowing microtubules to promote nuclear deformability. This induces local nuclear reorganisation and changes in chromatin compaction and in 3D genome organisation. Importantly, we observed that a similar mechanism contributes to the irregular nuclear shape of megakaryocytes, physiological polyploid cells generated by MS. Overall, these data highlight the fact that different means to generate WGD have different outcomes, which may influence downstream cell functions.

## Results

### Mitotic slippage, unlike cytokinesis failure and endoreplication, generates tetraploid cells with nuclear deformations

To define whether different routes of WGD have different outcomes, we induced unscheduled WGD through mitotic slippage (MS), cytokinesis failure (CF) or endoreplication (EnR) in RPE-1 cells (Fig. 1A; Appendix Fig. S1A) (Gemble et al, 2022; Bernhard et al, 2023), which are a p53-proficient diploid human cell line. Briefly, we induced MS by inhibiting Eg5 and MPS1 or by depleting CCNB1 using an AID degron—both approaches lead to premature mitotic exit before chromosome segregation. To induce CF, we used either a myosin inhibitor or an MKLP2 inhibitor, both of which prevent cytokinesis. Lastly, for EnR, we used a mitotic kinase inhibitor, or we depleted CCNA2 using an AID degron to prevent mitotic entry (see methods) (Gemble et al, 2022; Bernhard et al, 2023). As previously described, these strategies generate a mixed population of diploid (2N) and tetraploid (4N) cells (see Methods) that can be distinguished by nuclear and cell size (Gemble et al, 2022). Importantly, to avoid any effect due to cell cycle progression, cells were synchronised in the first G1 upon WGD using the CDK4/6 inhibitor Palbociclib (Fry et al, 2004) at 1 µM.

Using these highly comparable systems, we observed that MS, but not CF and EnR, generated tetraploid cells with abnormal nuclear shape (Fig. 1B). To quantitatively analyse the nuclear architecture in these cells, we measured nuclear circularity and solidity, two indices, informing on nuclear elongation and on the presence of nuclear invaginations, respectively (Fig. 1C). We observed that tetraploid cells generated through MS showed decreased nuclear circularity and solidity, while CF and EnR showed values similar to diploid cells (Fig. 1D–F). Similar results were obtained using alternative methods and other diploid cell lines—BJ and HCT116 (Appendix Fig. S1A–H). Interestingly, CF also generates a small percentage of mononucleated cells that do not exhibit nuclear deformation (Appendix Fig. S1I). This suggests that premature mitotic exit with a single tetraploid nucleus is not sufficient to cause nuclear deformations. Additionally, when cells were cultured in 3D, nuclear deformations upon MS were still detected (Appendix Fig. S1J–M). Inducing MS in asynchronous cells, also showed similar results (Appendix Fig. S1N). We also found that the nuclear area was similar in MS- and EnR-generated newly born tetraploid RPE-1 and HCT116 cell lines (Appendix Fig. S1O,P), suggesting that nuclear defects upon MS do not affect the overall nuclear size but only nuclear architecture.

We next performed live imaging approaches using cells stably expressing mCherry-H2B, to follow the transition from G2 to the subsequent G1 at the single cell level. In control diploid cells that undergo canonical mitotic divisions, nuclear circularity and solidity from G2 to G1 remained stable (Fig. 1G,H; Expanded view Movie 1). In contrast, in tetraploid cells generated by MS, an obvious decrease could be noticed (Fig. 1G,H; Expanded view Movie 2). Plotting the delta circularity (defined as the difference between nuclear circularity in G2 vs in G1) showed values close to 0 in diploid cells (Fig. 1I). This indicates that nuclear architecture was similar before and after mitosis in diploid cells - i.e. nuclear architecture was stable over subsequent cell cycles, as expected. Delta circularity increases in MS-induced tetraploid cells, indicating that MS impairs the re-establishment of a typical nuclear architecture in newly born tetraploid cells. Indeed, we observed that MS-induced tetraploid G1 cells exhibited heterogenous nuclear shapes (Fig. 1J), that were still present in the following G2 (Appendix Fig. S1Q). To understand whether these abnormal shapes were maintained over multiple cell cycles, we induced MS in a p53-depleted RPE-1 cell line, which is permissive to tetraploid cell proliferation (Gemble et al, 2022; Ganem et al, 2014; Vittoria et al, 2018; Kuffer et al, 2013). Nuclear circularity and solidity remained low over multiple cell cycles in MS-generated tetraploid cells (Appendix Fig. S1R–T).

At the population level, we were struck by the level of heterogenous nuclear shapes upon MS. Indeed, we found that MS generated a population of tetraploid cells displaying a wide range of nuclear circularity and solidity indexes (Fig. 1J,K). This reflects that WGD, when induced through MS, generates a high degree of nuclear shape diversity that seems stochastic—a phenomenon that we called here nuclear instability. In contrast, diploid, CF- and EnR-generated tetraploid cells are clustered in a homogenous cell population with nuclear circularity and solidity indexes close to 1, indicating that these cells have a uniform nuclear architecture (Fig. 1J,K).

Together, these findings show that MS generates nuclear instability—i.e., a high degree of nuclear shape diversity, while CF and EnR induction do not impair nuclear morphology.

### Upon mitotic slippage, cells enter G1 with high H3S10 phosphorylation levels

**Figure 2:**
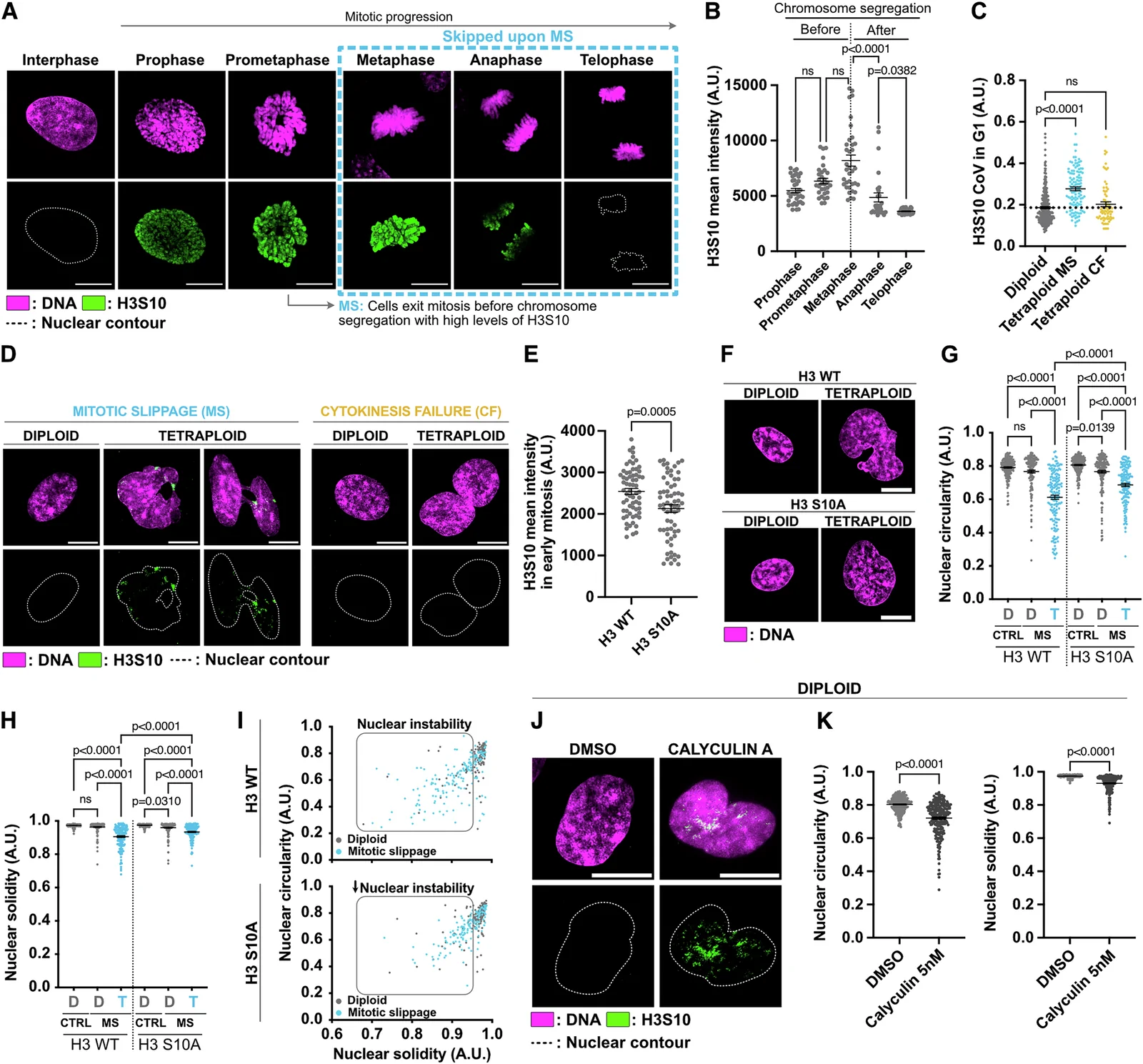
High levels of H3S10 phosphorylation contribute to nuclear deformations upon mitotic slippage. (**A**) SIM representative images showing diploid RPE-1 mitotic cells. H3S10 in green, DNA in magenta. (**B**) Graph showing H3S10 mean intensity during mitosis in diploid RPE-1 cells. Mean ± SEM, >25 mitotic cells analysed per condition, three independent experiments. (**C**) Graph showing the H3S10 coefficient of variation (CoV—defined as the ratio of the standard deviation to the mean) in G1 in diploid (in grey) or tetraploid RPE-1 cells generated by MS (in blue) or by CF (in yellow). Mean ± SEM, >75 cells analysed per condition, three independent experiments. (**D**) SIM representative images showing diploid and tetraploid G1 RPE-1 cells generated by MS or CF. H3S10 in green, DNA in magenta. (**E**) Graph showing the H3S10 mean intensity in RPE-1 diploid mitotic cells stably expressing H3 WT and H3S10A. Mean ± SEM, >65 mitotic cells analysed per condition, three independent experiments. (**F**) Representative images showing diploid and tetraploid G1 RPE-1 cells generated by MS and stably expressing H3 WT or H3S10A. DNA in magenta. (**G**,** H**) Graph showing the nuclear circularity (**G**) or solidity (**H**) index in diploid (in grey) and in MS-generated tetraploid (in blue) G1 RPE-1 cells stably expressing H3 WT or H3S10A. Mean ± SEM, >150 cells analysed per condition, three independent experiments. (**I**) Graphs showing the nuclear circularity and solidity index from (**G**–**H**) in diploid (in grey) and in tetraploid G1 RPE-1 cells (in blue) generated by MS. (**J**) SIM representative images showing diploid cells treated with DMSO or 5 nM Calyculin. H3S10 in green, DNA in magenta. (**K**) Graphs showing the nuclear circularity and solidity index in diploid RPE-1 cells treated with DMSO or 5 nM Calyculin. Mean ± SEM, >230 G1 cells analysed per condition, three independent experiments. Scale bar: 10 µm. D diploid, T tetraploid, MS mitotic slippage, CF cytokinesis failure, AU arbitrary unit. (**B**,** C**,** G**,** H**) ANOVA test (one-sided). (**E**,** K**) *t*-test (two-sided). ns not significant. Source data are available online for this figure.

We then hypothesised that the nuclear instability specific to MS could result from the fact that cells exit mitosis in a state which is not compatible with optimal nuclear envelope reformation. During mitotic entry, phosphorylation of Ser10 of Histone 3 (H3S10) promotes nuclear envelope breakdown (Poleshko et al, 2019; Prigent and Dimitrov, 2003; Hendzel et al, 1997) (Fig. 2A,B). As cells exited mitosis, the phosphorylation levels gradually decreased, allowing nuclear envelope reformation (Poleshko et al, 2019; Prigent and Dimitrov, 2003) (Fig. 2A,B). Since EnR cells do not enter mitosis, H3S10 phosphorylation levels should remain low throughout the cell cycle, while CF cells exit mitosis in a state in which H3S10 phosphorylation levels should also be low (Poleshko et al, 2019; Prigent and Dimitrov, 2003). In contrast, MS cells, that exit mitosis earlier, should display high H3S10 phosphorylation levels when exiting mitosis (Poleshko et al, 2019; Prigent and Dimitrov, 2003). In line with this model, we found using structural illumination microscopy that MS-induced tetraploid cells still have H3S10 phosphorylation in the first G1 upon WGD - forming patches close to the nuclear envelope (Fig. 2C,D). In contrast, diploid or CF-induced tetraploid cells do not exhibit H3S10 phosphorylation in G1 (Fig. 2C,D), as expected (Poleshko et al, 2019; Prigent and Dimitrov, 2003).

We next wanted to directly test the contribution of high H3S10 phosphorylation levels to nuclear architecture. We generated a cell line, expressing an H3 mutant version, where Serine 10 was replaced by an Alanine residue - H3S10A (Poleshko et al, 2019), in the background of wild type (WT) H3. A partial decrease in H3S10 phosphorylation during mitosis was noticed as expected (Fig. 2E). Importantly, this was sufficient to increase both nuclear circularity and solidity in newly born tetraploid MS-induced cells, improving their overall nuclear shape and thus decreasing nuclear instability (Fig. 2F–I). To confirm that maintaining H3S10 phosphorylation in G1 was sufficient to generate nuclear deformations, we inhibited in diploid cells the phosphatase responsible for H3S10 dephosphorylation at mitotic exit using low doses of Calyculin A (Ishihara et al, 1989). This treatment resulted in increased H3S10 phosphorylation levels in diploid G1 cells and remarkably, nuclear deformations characterised by invaginations similar to those found in MS-generated tetraploid cells were noticed (Fig. 2J,K).

Together, our results show that low H3S10 phosphorylation at mitotic exit is a pre-requisite to re-establish proper nuclear morphology during nuclear re-assembly.

### Mitotic slippage generates tetraploid cells with high histone acetylation levels and low nuclear stiffness

**Figure EV1:**
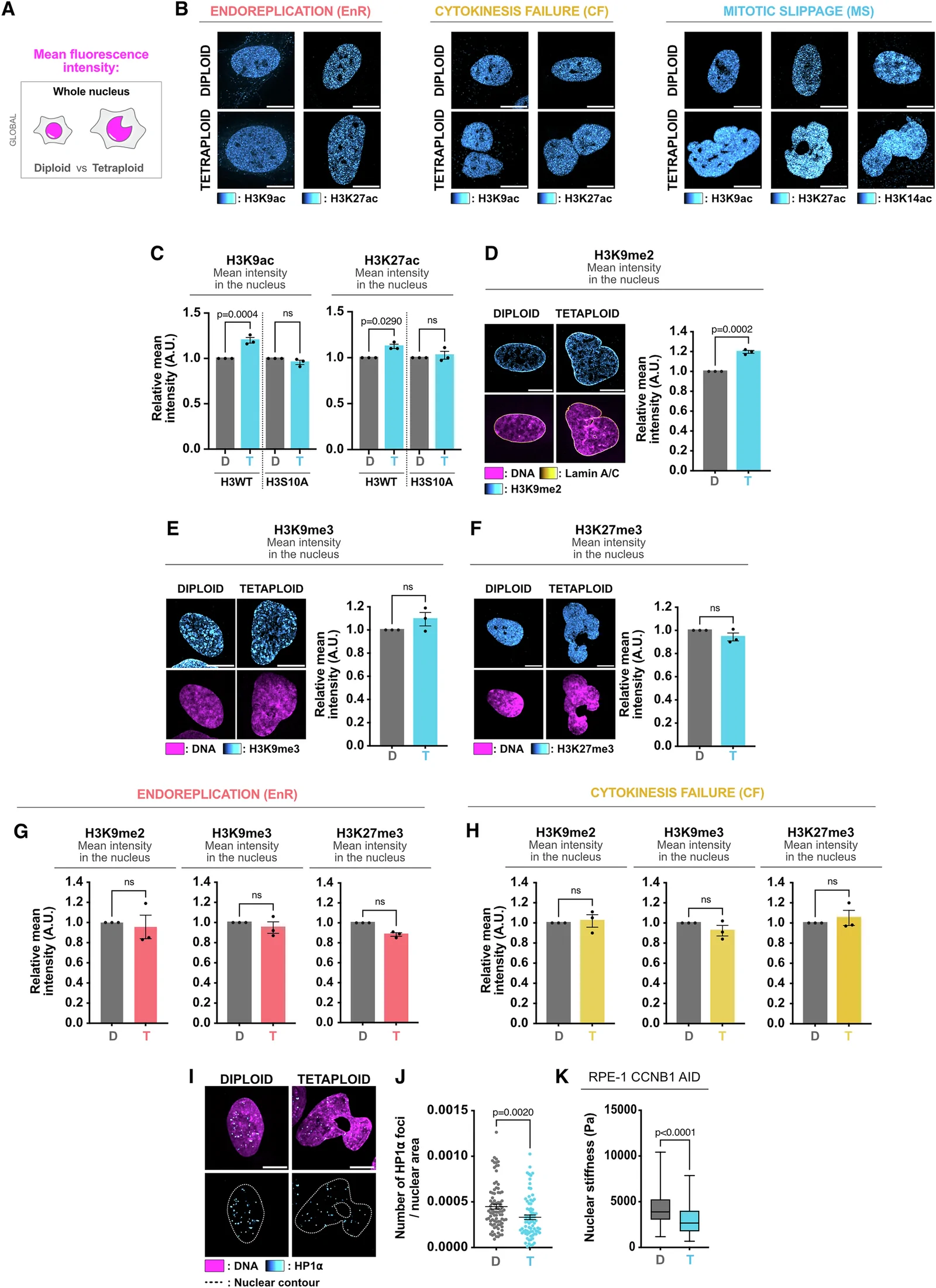
Mitotic slippage, but not cytokinesis failure and endoreplication, alters histone modification. (**A**) Schematic representation of the mean fluorescence measured at the level of the whole nucleus in diploid and tetraploid cells. (**B**) SIM representative images showing diploid and tetraploid G1 RPE-1 cells generated by EnR (left panel), by CF (central panel) or by MS (right panel). H3K9ac, H3K27ac and H3K14ac in cyan hot. (**C**) Graphs showing the relative H3K9ac (left panel) or H3K27ac (right panel) mean intensity in the nucleus in diploid (in grey) and in tetraploid (in blue) RPE-1 G1 cells stably expressing H3 WT or H3S10A. Mean ± SEM, >100 G1 cells analysed per condition, three independent experiments. (**D**) Left panel—SIM representative images showing diploid and tetraploid G1 RPE-1 cells generated by MS. Lamin A/C in yellow hot, H3K9me2 in cyan hot and DNA in magenta. Right panel—Graph presenting the relative H3K9me2 mean intensity in the nucleus in diploid (in grey) and in tetraploid (in blue) G1 RPE-1 cells. Mean ± SEM, >100 G1 cells analysed per condition, three independent experiments. (**E**) Left panel—SIM representative images showing diploid and tetraploid G1 RPE-1 cells generated by MS. Lamin A/C in yellow hot, H3K9me3 in cyan hot and DNA in magenta. Right panel—Graph presenting the relative H3K9me3 mean intensity in the nucleus in RPE-1 diploid (in grey) and in tetraploid (in blue) G1 cells. Mean ± SEM, >100 G1 cells analysed per condition, three independent experiments. (**F**) Left panel—SIM representative images showing diploid and tetraploid G1 RPE-1 cells generated by MS. Lamin A/C in yellow hot, H3K27me3 in cyan hot and DNA in magenta. Right panel—Graph presenting the Relative H3K27me2 mean intensity in the nucleus in diploid (in grey) and in tetraploid (in blue) G1 RPE-1 cells. Mean ± SEM, >100 G1 cells analysed per condition, three independent experiments. (**G**,** H**) Graphs showing the relative H3K9me2, H3K9me3 and H3K27me3 mean intensity in the nucleus in diploid (in grey) and in tetraploid G1 RPE-1 cells generated by (**G**) EnR (in red) or by (**H**) CF (in yellow). Mean ± SEM, >100 G1 cells analysed per condition, three independent experiments. (**I**) SIM representative images showing diploid and tetraploid G1 RPE-1 cells generated by MS. HP1α in cyan hot and DNA in magenta. (**J**) Graph showing the number of HP1 foci / nuclear area in diploid (in grey) and in tetraploid (in blue) RPE-1 G1 cells. Mean ± SEM, >90 G1 cells analysed per condition, at least three independent experiments. (**K**) Graph showing nuclear stiffness in diploid and in MS-generated tetraploid G1 RPE-1 CCNB1 cells (in grey and blue, respectively). MS cells were generated by depleting CCNB1. Box and whiskers ± Min to Max, >175 G1 cells analysed per condition, three independent experiments. Scale bars: 10 µm. D diploid, T tetraploid, MS mitotic slippage, EnR endoreplication, CF cytokinesis failure, AU arbitrary unit. (**C**–**H**,** J**) *t*-test (two-sided). (**K**) Kolmogorov–Smirnov test. ns not significant.

We then investigated how H3S10 phosphorylation in tetraploid G1 cells contributes to generate nuclear deformations upon MS. Previous work identified that H3S10 phosphorylation impacts other histone modifications (Chen et al, 2018; Peng et al, 2018; Komar and Juszczynski, 2020; Mateescu et al, 2004). We thus quantified histone acetylation and methylation levels using structural illumination microscopy. The fluorescence intensity levels were measured in diploid and tetraploid G1 cells at the level of the whole nucleus (Fig. EV1A).

**Figure 3:**
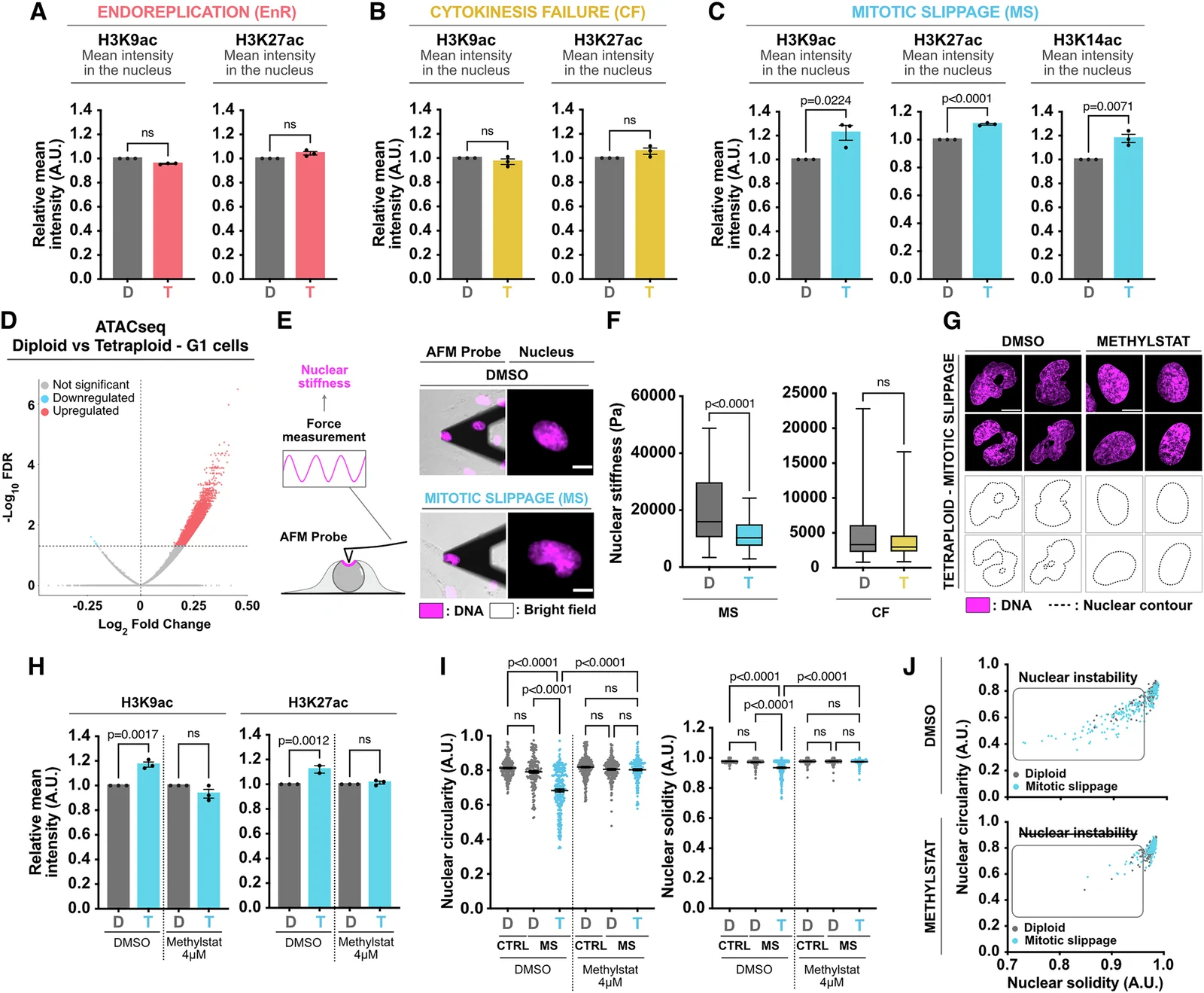
Mitotic slippage generates tetraploid cells with high histone acetylation levels and softer nuclei promoting nuclear deformations. (**A**–**C**) Graphs showing H3K9ac, H3K27ac and H3K14ac levels in diploid (in grey) and in tetraploid G1 RPE-1 cells generated by EnR ((**A)**, in red), by CF ((**B**), in yellow) or by MS ((**C**), in blue). Mean ± SEM, >100 cells analysed per condition, three independent experiments. (**D**) Volcano plot showing ATACseq data comparing diploid and tetraploid G1 RPE-1 cells generated by MS. 100 000 cells analysed per condition. Two independent experiments. (**E**) Left panel—Schematic representation showing the principle of atomic force microscopy (AFM). Right panel—Representative images showing diploid and MS-generated tetraploid G1 RPE-1 cells. DNA in magenta. Brightfield in grey. (**F**) Graph showing nuclear stiffness in diploid (in grey) and in tetraploid G1 RPE-1 cells generated by MS (in blue) or by CF (in yellow). Box and whiskers. Mean ± min to max, >90 cells (left panel) or >65 cells (right panel) analysed per condition, three independent experiments. (**G**) Representative images showing diploid and tetraploid G1 RPE-1 cells generated by MS treated with DMSO or 4 µM Methylstat. DNA in magenta. (**H**) Graph showing H3K9ac and H3K27ac levels in diploid (in grey) and in MS-generated tetraploid (in blue) G1 RPE-1 cells treated with DMSO or 4 µM Methylstat. Mean ± SEM, >100 cells analysed per condition, three independent experiments. (**I**) Graph showing the nuclear circularity and solidity indices in diploid (in grey) and in MS-generated tetraploid (in blue) G1 RPE-1 cells treated with DMSO or 4 µM Methylstat. Mean ± SEM, >125 cells analysed per condition, three independent experiments. (**J**) Graph showing the nuclear circularity and solidity indices from (**I**) in diploid (in grey) and tetraploid G1 RPE-1 cells generated by MS (in blue) treated with DMSO or 4 µM Methylstat. Scale bars: 10 µm. D diploid, T tetraploid, MS mitotic slippage, EnR endoreplication, CF cytokinesis failure, AU arbitrary unit. (**F**) Kolmogorov–Smirnov test. (**A**–**C**,** H**) *t*-test (two-sided). (**I**) ANOVA test (one-sided). ns not significant. Source data are available online for this figure.

We first noticed a global increase in H3K9ac, H3K27ac and H3K14ac levels in tetraploid G1 cells generated by MS (Figs. 3A–C and EV1B). In contrast, in CF- or EnR-induced tetraploid G1 cells, histone acetylation remained similar to diploid cells (Figs. 3A–C and EV1B). Importantly, reducing H3S10 phosphorylation by expressing H3S10A mutant was sufficient to prevent the increase in acetylation levels observed upon MS (Fig. EV1C). This suggests that high H3S10 phosphorylation levels upon MS induced a global increase in histone acetylation in tetraploid G1 cells. Since histone acetylation is commonly associated with open chromatin/euchromatin (Chen et al, 2022), we performed ATACseq and found that chromatin accessibility was higher in MS-induced tetraploid G1 cells compared to diploid cells (Fig. 3D).

When measuring histone methylation, usually associated with closed chromatin/heterochromatin (Maison and Almouzni, 2004; James et al, 1989), we found that only H3K9me2 levels were increased upon MS, while H3K9me3 and H3K27me3 levels remained similar to diploid cells (Fig. EV1D–F). In CF- or EnR-generated cells, no changes in histone methylation levels were found (Fig. EV1G,H). Interestingly, we also observed that MS led to a decrease in the number of Heterochromatin Protein 1α (HP1α) foci (Fig. EV1I,J), a key component known to promote nuclear compaction (Allshire and Madhani, 2018). Together, these findings demonstrate that MS impairs histone compaction in newly born tetraploid cells, favouring an overall open chromatin.

It is well established that several factors, including chromatin states, contribute to nuclear envelope deformability, counteracting cytoplasmic forces that act on the nucleus (Stephens et al, 2019; Miroshnikova and Wickström, 2022). It is therefore possible that the nuclear deformations of tetraploid cells induced through MS reflected a decrease in nuclear mechanical properties due to changes in chromatin compaction as described above. To test this possibility, we first tested whether MS alters nuclear stiffness using atomic force microscopy (AFM) (Fig. 3E). Using two different strategies to generate MS, we found that G1 tetraploid nuclei were significantly softer than their diploid counterparts (Figs. 3F and EV1K). In contrast, CF-induced tetraploid G1 cells exhibited nuclear stiffness similar to diploid cells (Fig. 3F).

**Figure EV2:**
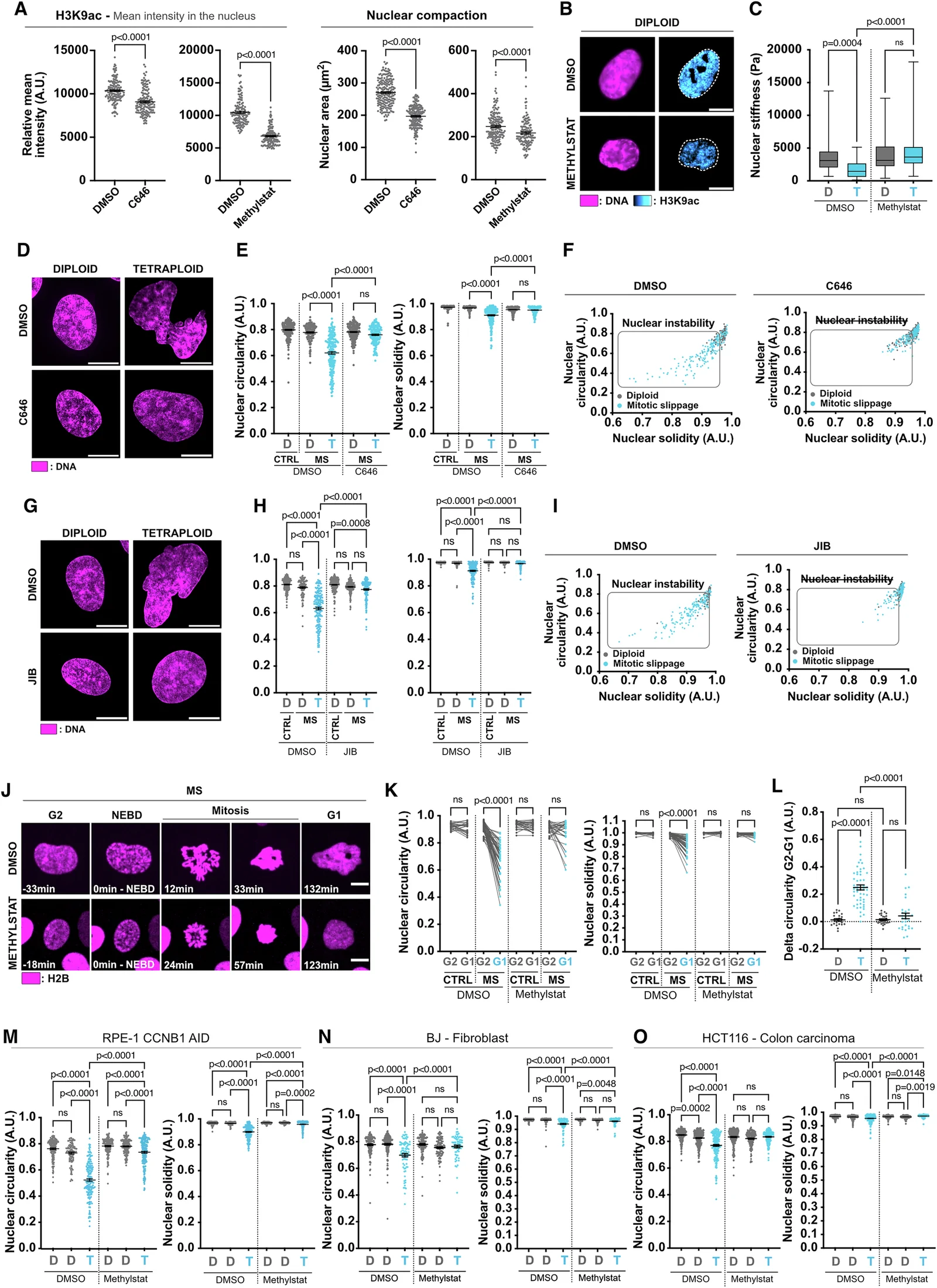
Promoting nuclear compaction is sufficient to prevent nuclear deformations upon mitotic slippage. (**A**) Graphs presenting the H3K9ac mean intensity (left panel) or the nuclear area (right panel) in diploid RPE-1 cells treated with DMSO or with Methylstat 4 µM or with C646 20 µM. Mean ± SEM, >125 cells analysed per condition, at least three independent experiments. (**B**) Representative images showing RPE-1 diploid cells treated with DMSO or 4 µM Methylstat. H3K9ac in cyan hot, DNA in magenta. (**C**) Graph showing the nuclear stiffness in diploid (in grey) and tetraploid (in blue) RPE-1 G1 cells generated by MS treated with DMSO or 4 µM Methylstat. (**D**) Representative images showing diploid and tetraploid RPE-1 G1 cells treated with DMSO or 20 µM C646. DNA in magenta. (**E**) Graphs showing the nuclear circularity (left panel) and solidity (right panel) indices in diploid (in grey) and tetraploid (in blue) RPE-1 G1 cells generated by MS treated with DMSO or 20 µM C646. Mean ± SEM, >125 G1 cells analysed per condition, at least three independent experiments. (**F**) Graphs presenting the nuclear circularity and solidity from (**E**) in RPE-1 diploid (in grey) and tetraploid (in blue) G1 cells treated with DMSO or 20 µM C646. (**G**) Representative images showing diploid and tetraploid RPE-1 cells treated with DMSO or 5 µM JIB. DNA in magenta. (**H**) Graphs presenting the nuclear circularity (left panel) and solidity (right panel) indices in diploid (in grey) and tetraploid (in blue) RPE-1 G1 cells generated by MS treated with DMSO or 5 µM JIB. Mean ± SEM, >100 G1 cells analysed per condition, at least three independent experiments. (**I**) Graphs presenting the nuclear circularity and solidity from (**H**) in diploid (in grey) and tetraploid (in blue) RPE-1 G1 cells treated with DMSO or 5 µM JIB. (**J**) Stills from time-lapse imaging of RPE-1 cells stably expressing mCherry-H2B (in magenta) performing MS and treated with DMSO (upper panel) or 2 µM Methylstat (lower panel). (**K**) Graphs showing the nuclear circularity (left panel) and solidity (right panel) measured at the single cell levels in G2 and in G1 upon mitotic division (in grey) or MS (in blue) in RPE-1 cells treated with DMSO or 2 µM Methylstat. >30 cells analysed per condition, three independent experiments. (**L**) Graph showing the delta circularity (defined as the difference between nuclear circularity in G2 vs in G1) upon mitotic division (in grey) or MS (in blue) in RPE-1 cells treated with DMSO or 2 µM Methylstat. Mean ± SEM, >25 cells analysed per condition, three independent experiments. (**M**–**O**) Graphs displaying nuclear circularity and solidity in (**M**) RPE-CCNB1, (**N**) BJ or (**O**) HCT116 cells treated with DMSO or 4 µM Methylstat. (**M**) Tetraploid cells were generated by depleting CCNB1. Mean ± SEM, >65 G1 cells analysed per condition, three independent experiments. Scale bars: 10 µm. D diploid, T tetraploid, MS mitotic slippage, NEBD nuclear envelope breakdown, AU arbitrary unit. (**A**,** K**,** L**) *t*-test (two-sided). (**E**,** H**,** M**–**O**) ANOVA test (one-sided). (**C**) Kolmogorov–Smirnov test. ns not significant.

We next investigated the functional relationship between higher acetylation levels, decreased nuclear stiffness and nuclear deformations upon MS. We used two complementary strategies to decrease acetylation levels in MS-induced tetraploid cells. We treated cells with (1) C646, a histone acetylase inhibitor (Bowers et al, 2010), which decreases histone acetylation levels (Fig. EV2A) or with (2) Methylstat, a histone demethylase inhibitor (Stephens et al, 2018), which promotes histone methylation, preventing histone acetylation on the same residues (Fig. EV2A,B). These treatments were sufficient to decrease histone acetylation and to promote nuclear compaction and thus nuclear stiffness, preventing nuclear deformations and nuclear instability in MS-induced tetraploid G1 cells (Figs. 3G–J and EV2A–F). These results were confirmed using JIB-04, another histone demethylase inhibitor (Wang et al, 2013), by live imaging, using another method to induce MS and in BJ and HCT116 cells (Fig. EV2G–O).

These data show that MS generates tetraploid cells with high levels of chromatin acetylation that contribute to decrease their nuclear stiffness.

### Microtubules generate nuclear invaginations in MS-induced tetraploid G1 cells

**Figure 4:**
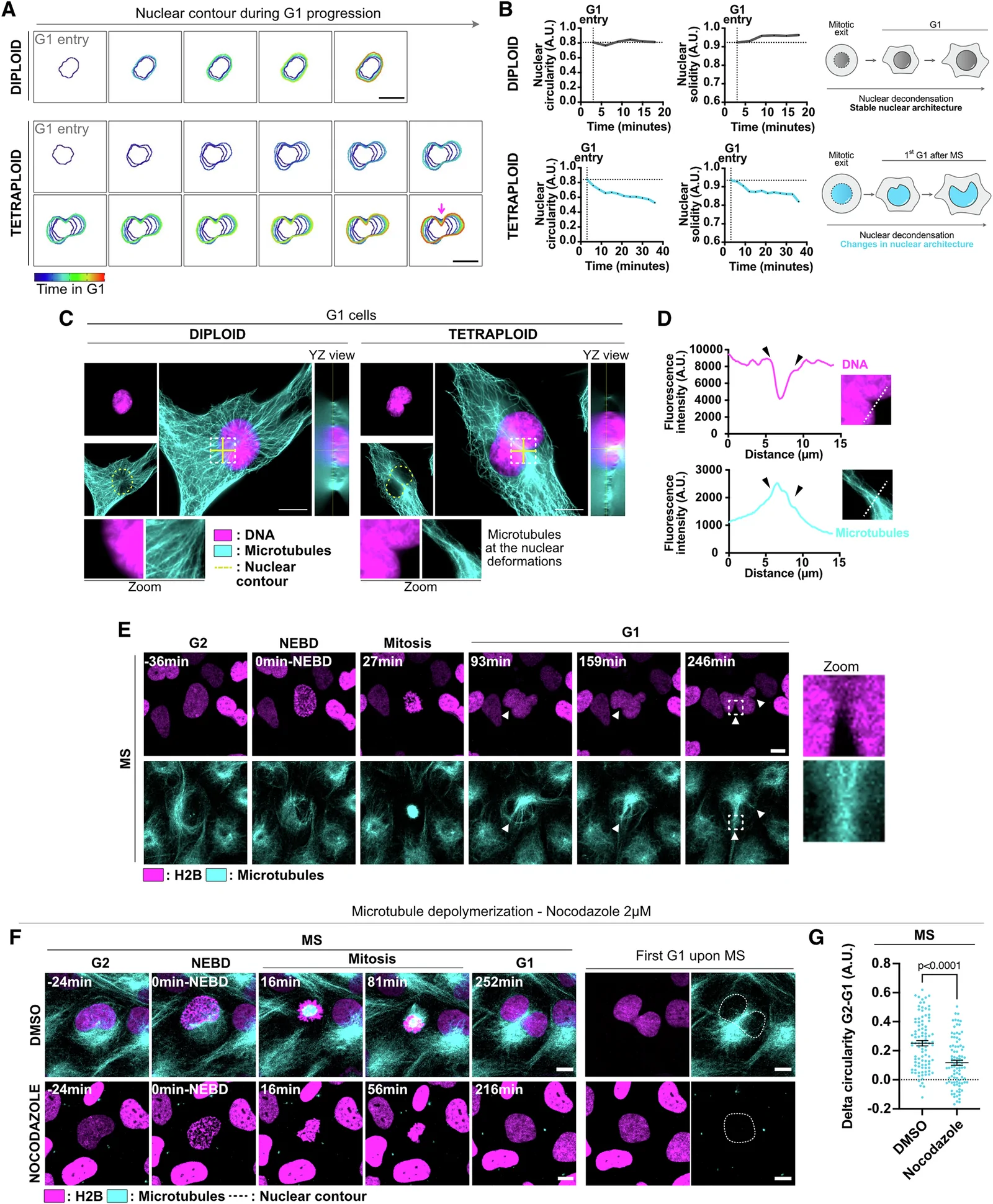
The microtubule cytoskeleton contributes to nuclear deformations in mitotic slippage-induced newly born tetraploid cells. (**A**) Stills from time-lapse imaging of RPE-1 cells stably expressing mCherry-H2B after mitotic division (upper panel) or MS (lower panel). The nuclear contour during G1 is indicated, and the colour code shows the time spent in G1 (see methods). The pink arrow points at a nuclear deformation. (**B**) Left panel—Graph showing the nuclear circularity and solidity in G1 upon mitotic division (in grey, upper panel) or MS (in blue, lower panel). Right panel—Schematic representations of nuclear decondensation upon mitotic division (in grey, upper panel) or MS (in blue, lower panel). The data were measured in the cells presented in (**A**). (**C**) Representative images of diploid and MS-generated tetraploid RPE-1 G1 cells. Microtubules in cyan, DNA in magenta. The nuclear contour is indicated (yellow dotted line). (**D**) Graphs showing line scans measurements from (**C**). (**E**) Stills from time-lapse imaging of RPE-1 cells stably expressing mCherry-H2B (in magenta) undergoing MS. Microtubules in cyan. (**F**) Stills from time-lapse imaging of RPE-1 cells stably expressing mCherry-H2B (in magenta) undergoing MS and treated with DMSO (upper panel) or 2 µM Nocodazole (lower panel). Microtubules in cyan. (**G**) Graph presenting the delta circularity (defined as the difference between nuclear circularity in G2 vs in G1) in MS-generated tetraploid cells treated with DMSO or 2 µM Nocodazole. Mean ± SEM, >90 cells analysed per condition, three independent experiments. Scale bars: 10 µm. D diploid, T tetraploid, MS mitotic slippage, NEBD nuclear envelope breakdown, AU arbitrary unit. (**G**) *t*-test (two-sided). ns not significant. Source data are available online for this figure.

Detailed analysis of mitotic exit in diploid and MS-induced tetraploid cells revealed an increase in nuclear area, most likely due to chromosome decondensation (Moreno-Andrés et al, 2023). However, in tetraploid cells, this was accompanied by the appearance of nuclear invaginations (Fig. 4A, pink arrow). Indeed, while quantifying nuclear circularity and solidity, we found that these indices were quite stable in diploid cells during early G1 (Fig. 4B, upper panel), whereas both parameters decreased in newly born tetraploid cells during mitotic exit (Fig. 4B, lower panel).

Cytoskeleton components interact with the nuclear envelope, a process that contributes to the establishment of nuclear architecture (Rothballer and Kutay, 2013). Characterisation of cytoskeleton proteins in G1 cells showed that microtubules (but not actin) accumulated at nuclear invaginations in MS-induced tetraploid cells (Fig. 4C,D; Appendix Fig. S2A), which was not seen in EnR-generated tetraploid cells (Appendix Fig. S2B). Using time-lapse microscopy in cells incubated with SPY-Tubulin and SPY-Actin, we followed microtubules and actin, respectively. We found that just after nuclear envelope reformation upon MS, microtubules accumulated at invagination sites, assembling in thick bundles, whereas actin did not (Fig. 4E; Appendix Fig. S2C; Expanded view Movies 3 and 4). These data suggest that microtubules, unlike actin, contribute to nuclear instability in newly born tetraploid cells.

To functionally test the contribution of microtubules in generating nuclear invaginations, we removed microtubules using nocodazole, a microtubule-depolymerising agent (De Brabander et al, 1977), just before MS. Strikingly, in the absence of microtubules, the nuclear shape in newly born tetraploid cells became more regular, presenting fewer deformations in G1, as also revealed by a decrease in the delta circularity (Fig. 4F,G; Expanded view Movies 5 and 6). Moreover, treating tetraploid cells with nocodazole was sufficient to enhance both nuclear circularity and solidity, thus reducing nuclear instability (Appendix Fig. S2D,E). These results were confirmed using another mean to induce MS (Appendix Fig. S2F–H; Expanded view Movies 7 and 8). We then tested whether stabilising microtubules could exacerbate nuclear deformations. To do so, we treated tetraploid cells with low doses of Taxol, a microtubule-stabilising agent (Manfredi and Horwitz, 1984), before MS. In these conditions, nuclear deformations were even more impressive, and a decrease in both nuclear circularity and solidity was noticed, associated with a higher nuclear instability (Appendix Fig. S2I,J).

We conclude that the microtubule cytoskeleton is the main contributor to nuclear deformations in newly born tetraploid cells induced by unscheduled MS.

### Nuclear deformations upon mitotic slippage leads to local nuclear reorganisation

**Figure 5:**
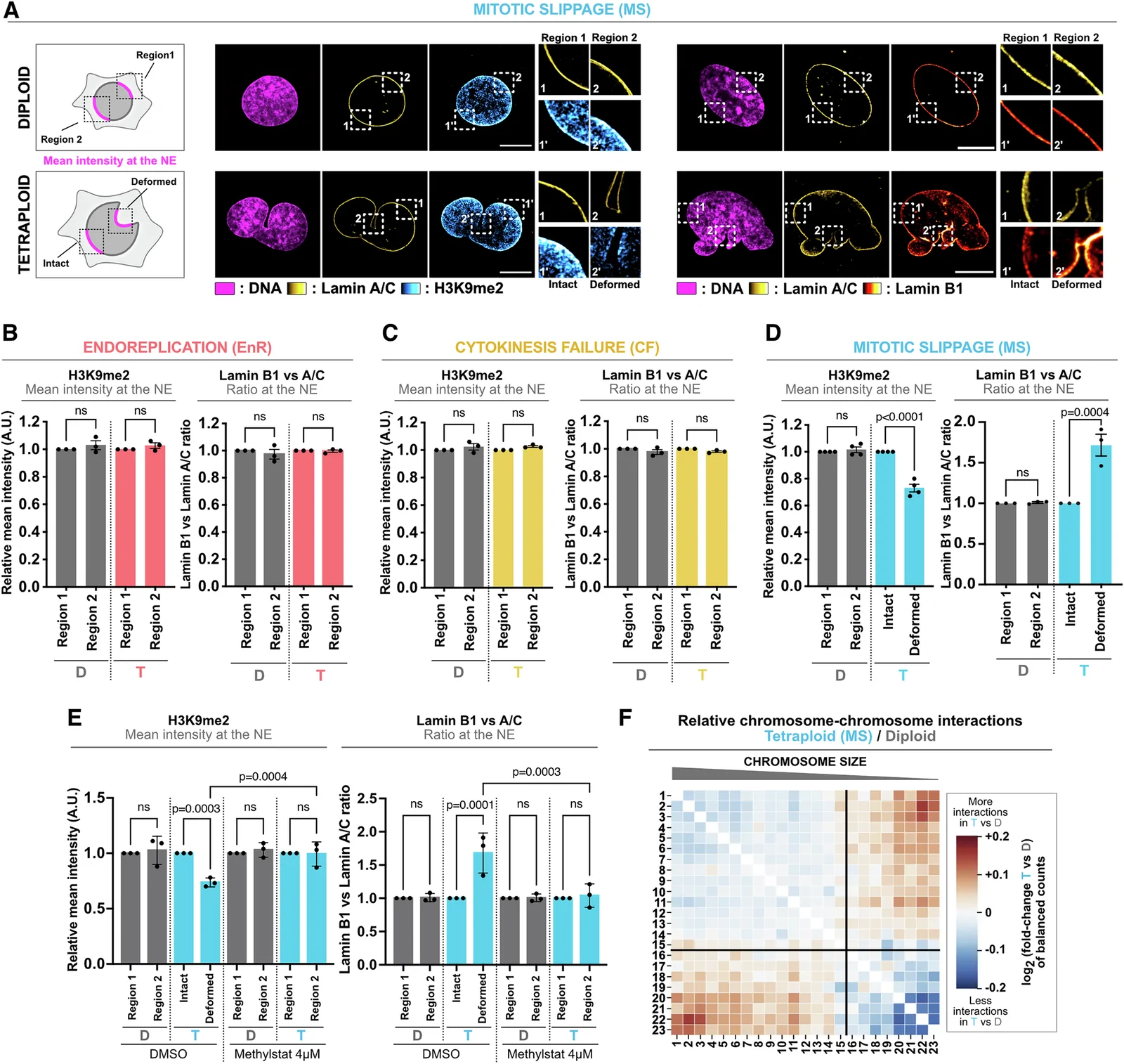
Mitotic slippage promotes local nuclear reorganisation and impairs 3D genome organisation. (**A**) Left, schematic representation of fluorescence intensity measured at the nuclear envelope or locally at two random regions (in diploid cells) and at intact and deformed regions (in tetraploid cells). Middle and right panels, SIM representative images showing diploid (top) and tetraploid (bottom) G1 RPE-1 cells generated by MS. Lamin A/C in yellow hot, H3K9me2 in cyan hot, Lamin B1 in red hot. DNA in magenta. (**B**–**D**) Graphs showing the relative H3K9me2 (left panel) or Lamin B1 vs A/C ratio (right panel) mean intensity at the nuclear envelope in diploid (in grey) and tetraploid G1 RPE-1 cells generated by EnR (**B**, in red), by CF (**C**, in yellow) or by MS (**D**, in blue). Mean ± SEM, >100 cells analysed per condition, three independent experiments (**E**). Graphs showing the relative H3K9me2 or Lamin B1 vs A/C ratio mean intensity at the nuclear envelope in diploid (in grey) and in tetraploid G1 RPE-1 cells generated by MS (in blue) treated with DMSO or 4 µM Methylstat. The same control was used in Fig. EV3M because the experiments were done together. Mean ± SEM, >100 cells analysed per condition, three independent experiments. (**F**) Heatmap of the ratios of inter-chromosomal contact enrichments (observed versus expected) between tetraploid (MS) and diploid G1 RPE-1 cells. Chromosomes were sorted by length. Scale bar: 10 µm. D diploid, T tetraploid, MS mitotic slippage, EnR endoreplication, CF cytokinesis failure, NE nuclear envelope, AU arbitrary unit. (**B**–**E**) *t*-test (two-sided). ns not significant. Source data are available online for this figure.

We then investigated whether the nuclear deformations upon MS have a local impact on nuclear architecture. To do so, we conducted a mini screen in which eight chromatin-related and six nuclear envelope-related candidates were visually examined using structural illumination microscopy. The candidate list was compiled based on their well-established roles in nuclear morphology (Stephens et al, 2019; Miroshnikova and Wickström, 2022; Dechat et al, 2010; Lammerding et al, 2006; Poleshko et al, 2019; Janin et al, 2017; Delaney et al, 2023), and the fluorescence intensity levels were measured in diploid and tetraploid G1 cells at intact and deformed nuclear envelope regions (Fig. 5A).

**Figure EV3:**
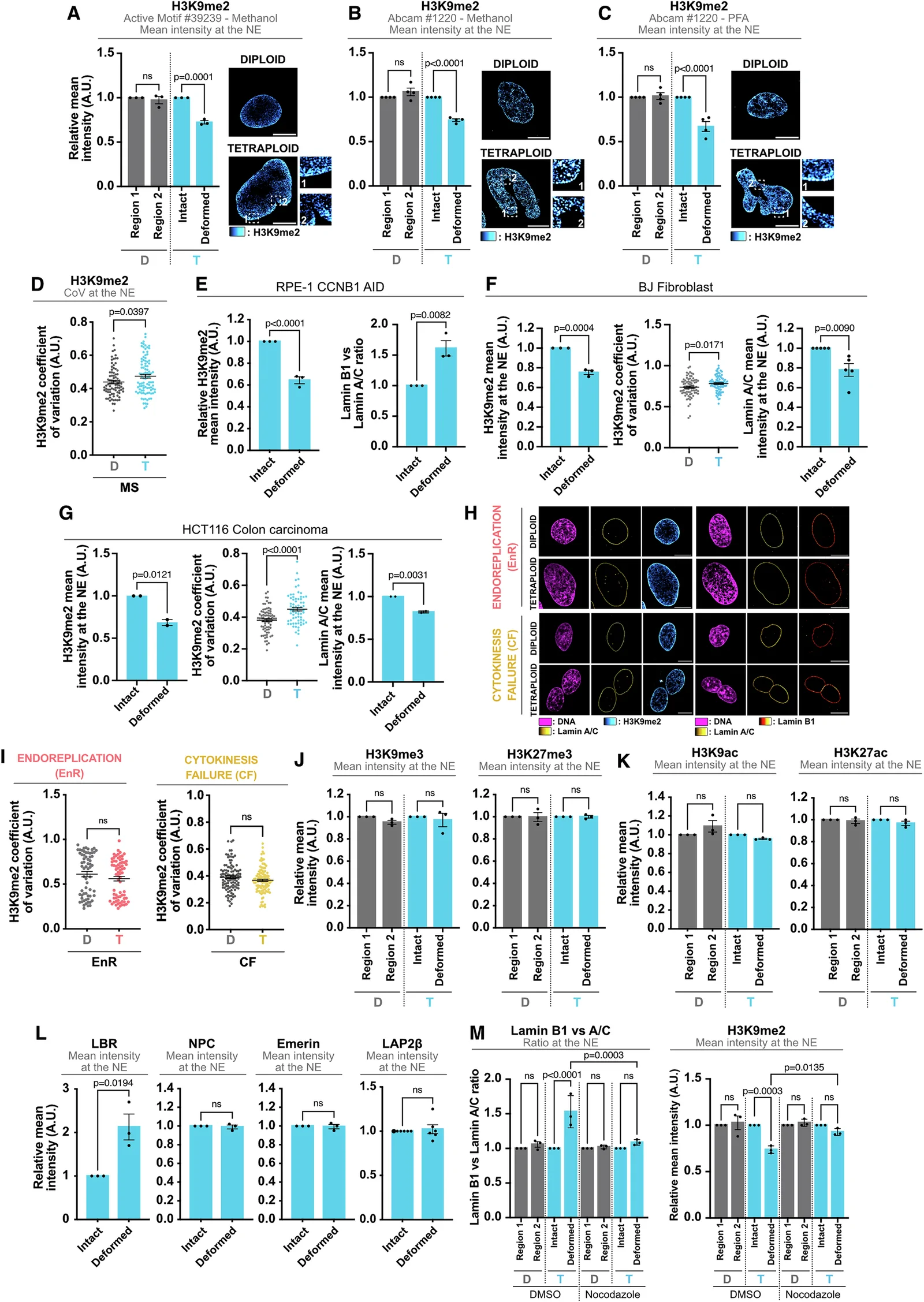
Mitotic slippage promotes local changes in the Lamina network and in H3K9me2 levels. (**A**) Left panel—Graph showing the relative H3K9me2 mean intensity at the nuclear envelope in diploid (in grey) and tetraploid (in blue) G1 RPE-1 cells. Mean ± SEM, >90 G1 cells analysed per condition, three independent experiments. Right panel—SIM representative images showing diploid and tetraploid G1 RPE-1 cells. H3K9me2 in cyan hot. (**B**) Left panel—Graph showing the relative H3K9me2 mean intensity at the nuclear envelope in diploid (in grey) and tetraploid (in blue) G1 RPE-1 cells. Mean ± SEM, >90 G1 cells analysed per condition, three independent experiments. Right panel—SIM representative images showing diploid and tetraploid G1 RPE-1 cells. H3K9me2 in cyan hot. (**C**) Left panel—Graph showing the relative H3K9me2 mean intensity at the nuclear envelope in diploid (in grey) and tetraploid (in blue) G1 RPE-1 cells. Mean ± SEM, >90 G1 cells SIM resolution representative images showing diploid and tetraploid G1 RPE-1 cells. H3K9me2 in cyan hot. (**D**) Graph presenting the H3K9me2 coefficient of variation at the nuclear envelope in MS-induced tetraploid G1 RPE-1 cells. Mean ± SEM, >90 G1 cells analysed per condition, three independent experiments. (**E**) Graphs showing the relative H3K9me2 mean intensity (left panel) or the Lamin B1 vs A/C ratio (right panel) at the nuclear envelope in MS-induced tetraploid RPE-1 CCNB1 cells. MS cells were generated by depleting CCNB1. Mean ± SEM, >90 G1 cells analysed per condition, three independent experiments. (**F**,** G**) Graphs of H3K9me2 mean intensity at the NE (left panel), H3K9me2 coefficient of variation at the NE (central panel) and Lamin A/C mean intensity at the NE (right panel) in diploid and in MS-generated tetraploid (**F**) BJ and (**G**) HCT116 cells. Mean ± SEM, >60 G1 cells analysed per condition, three independent experiments. (**H**) SIM representative images showing diploid and tetraploid cells generated by EnR or CF. H3K9me2, Lamin A/C and Lamin B1 in cyan hot, yellow hot and red hot, respectively. DNA in magenta. (**I**) Graphs showing the H3K9me2 coefficient of variation in diploid (in grey) and tetraploid G1 RPE-1 cells generated by EnR (in red) or CF (in yellow). Mean ± SEM, >90 G1 cells analysed per condition, three independent experiments. (**J**) Graphs showing the relative H3K9me3 (left panel) or H3K27me3 (right panel) mean intensity in diploid (in grey) and tetraploid (in blue) RPE-1 G1 cells. Mean ± SEM, >90 G1 cells analysed per condition, three independent experiments. (**K**) Graphs showing the relative H3K9ac (left panel) or H3K27ac (right panel) mean intensity in diploid (in grey) and tetraploid (in blue) RPE-1 G1 cells. Mean ± SEM, >90 G1 cells analysed per condition, three independent experiments. (**L**) Graphs presenting the relative mean intensity at the nuclear envelope of the indicated proteins in tetraploid RPE-1 G1 cells. Mean ± SEM, >90 G1 cells analysed per condition, at least three independent experiments. (**M**) Graphs showing the Lamin B1 vs A/C ratio (left panel) or the H3K9me2 mean intensity (right panel) at the nuclear envelope in diploid (in grey) and tetraploid (in blue) RPE-1 G1 cells treated with DMSO or with Nocodazole. The same control was used in Fig. 5E because the experiments were done together. Mean ± SEM, >90 G1 cells analysed per condition, three independent experiments. Scale bars: 10 µm. D diploid, T tetraploid, MS mitotic slippage, EnR endoreplication, CF cytokinesis failure, AU arbitrary unit. (**M**) ANOVA test (one-sided). (**A**–**G**,** I**–**L**) *t*-test (two-sided). ns not significant.

This mini screen identified that H3K9me2 levels were decreased in specific regions that coincide with nuclear deformations in MS-induced tetraploid cells (Fig. 5A–D). Because the tools used to characterise H3K9me2 have been the subject of controversy (PREPRINT: Chudzik et al, 2025), we additionally used another H3K9me2 antibody and two different protocols. Importantly, we obtained the same results (Fig. EV3A–C). H3K9me2 is normally enriched at the nuclear periphery and associated with chromatin regions that are less dynamic (Poleshko et al, 2019; Delaney et al, 2023). H3K9me2 coefficient of variation (CoV) at the nuclear envelope (defined as the ratio of the standard deviation to the mean) was increased in tetraploid cells generated by MS (Fig. EV3D), indicating uneven distribution of this modification, in sharp contrast with the uniform distribution of H3K9me2 at the nuclear envelope of diploid nuclei (Figs. 5A–D and EV3D). Similar defects in H3K9me2 localisation at invaginations were found in tetraploid cells where MS was induced by a different mean, and in BJ and HCT116 cell lines (Fig. EV3E–G). In contrast, we observed that both CF- and EnR-induced tetraploid cells displayed homogeneous distributions of H3K9me2 (Figs. 5A–C and EV3H,I). Moreover, no local changes in H3K9me3, H3K27me3, H3K9ac and H3K27ac levels, were noticed upon MS (Fig. EV3J,K).

The Lamina, composed of a specific ratio of Lamin A/C and Lamin B proteins, plays a crucial role in maintaining nuclear architecture (Miroshnikova and Wickström, 2022; Dechat et al, 2010; Lammerding et al, 2006; Ovsiannikova et al, 2021; Swift et al, 2013). The ratio between Lamin A/C and B is crucial in establishing nuclear morphology (Dechat et al, 2010; Lammerding et al, 2006; Ovsiannikova et al, 2021; Swift et al, 2013). Indeed, nuclear resistance is strongly impacted by Lamin A/C and a ratio dominated by Lamin B has thus been associated with soft tissues (Ovsiannikova et al, 2021; Swift et al, 2013). By measuring this ratio, we found differences in tetraploid cells at nuclear deformation regions. Frequently, Lamin B1 dominated the lamina network in invaginated domains, while the levels of Lamin A/C were lower than the ones found in intact regions (Fig. 5A–D). In line with this, we found that Lamin B Receptor (LBR) levels—a protein associated with soft tissues (Buxboim et al, 2017)—were also increased locally at the nuclear deformations upon MS, while we found no changes in Lap2**β**, Emerin and Nuclear pore complex levels (Fig. EV3L). Similar defects were observed in MS-generated tetraploid cells using an alternative method and in BJ and HCT116 cell lines (Fig. EV3E–G). In contrast, we observed that both EnR- and CF-induced tetraploid cells displayed homogeneous Lamin ratios (Figs. 5A–C and EV3H).

To define whether these local changes at the nuclear invaginations contribute to or are a consequence of the nuclear deformations, we prevented nuclear instability upon MS by reducing histone acetylation or by depolymerising microtubules. This was sufficient to restore homogeneous distributions of H3K9me2 and Lamin ratios in tetraploid cells (Figs. 5E and EV3M). These findings show that the local changes at the nuclear invaginations observed upon MS are not contributing to the nuclear deformations but are rather a secondary consequence of the local deformations by the microtubule cytoskeleton.

Together, our findings show that nuclear deformations upon MS promote local changes in nuclear organisation.

### Mitotic slippage impairs 3D genome organisation

We next investigated the functional consequences of nuclear deformations observed upon MS. We previously found that WGD was associated with high levels of DNA damage in the first S-phase upon tetraploidization, regardless of the method used to induce WGD (Gemble et al, 2022). Since only MS generates tetraploid cells with nuclear deformations, it is unlikely that such abnormal nuclear shapes contribute to DNA damage in newly born tetraploid cells. Consistent with this, we observed an inverse correlation between DNA damage and nuclear deformations upon MS (Appendix Fig. S3A,B). Nevertheless, these results suggest that the extent of nuclear deformations differentially affects genetic stability.

Recently, it has been shown that WGD in p53-deficient cells leads to loss of chromatin segregation, explained by an increased proportion of contacts between chromosomes, compartments, and chromatin domains (Lambuta et al, 2023). Insulation of topology-associated domains (TADs) requires a certain number of proteins that do not seem to scale with the tetraploid genome (Lambuta et al, 2023). We wanted to investigate whether such defects were already detectable in the first G1 after MS, in the presence of p53. To do so, we isolated diploid and tetraploid cells using flow cytometry, as previously described (Gemble et al, 2022) (Appendix Fig. S3C,D). Importantly, cell sorting did not affect nuclear deformations in MS-generated tetraploid cells (Appendix Fig. S3E). Using high-throughput chromatin capture conformation (HiC), we compared interactions at the chromosomal level and between chromatin domains in diploid and newly born MS-generated tetraploid G1 cells. Similarly to what has been previously observed, we found an increased number of contacts between short and long chromosomes (Fig. 5F) and between A and B compartments (Appendix Fig. S3F–I) in tetraploid cells compared to diploid cells.

Overall, our results identified that MS perturbs 3D genome organisation in newly born tetraploid cells.

### Megakaryocytes undergo mitotic slippage and display nuclear instability

**Figure 6:**
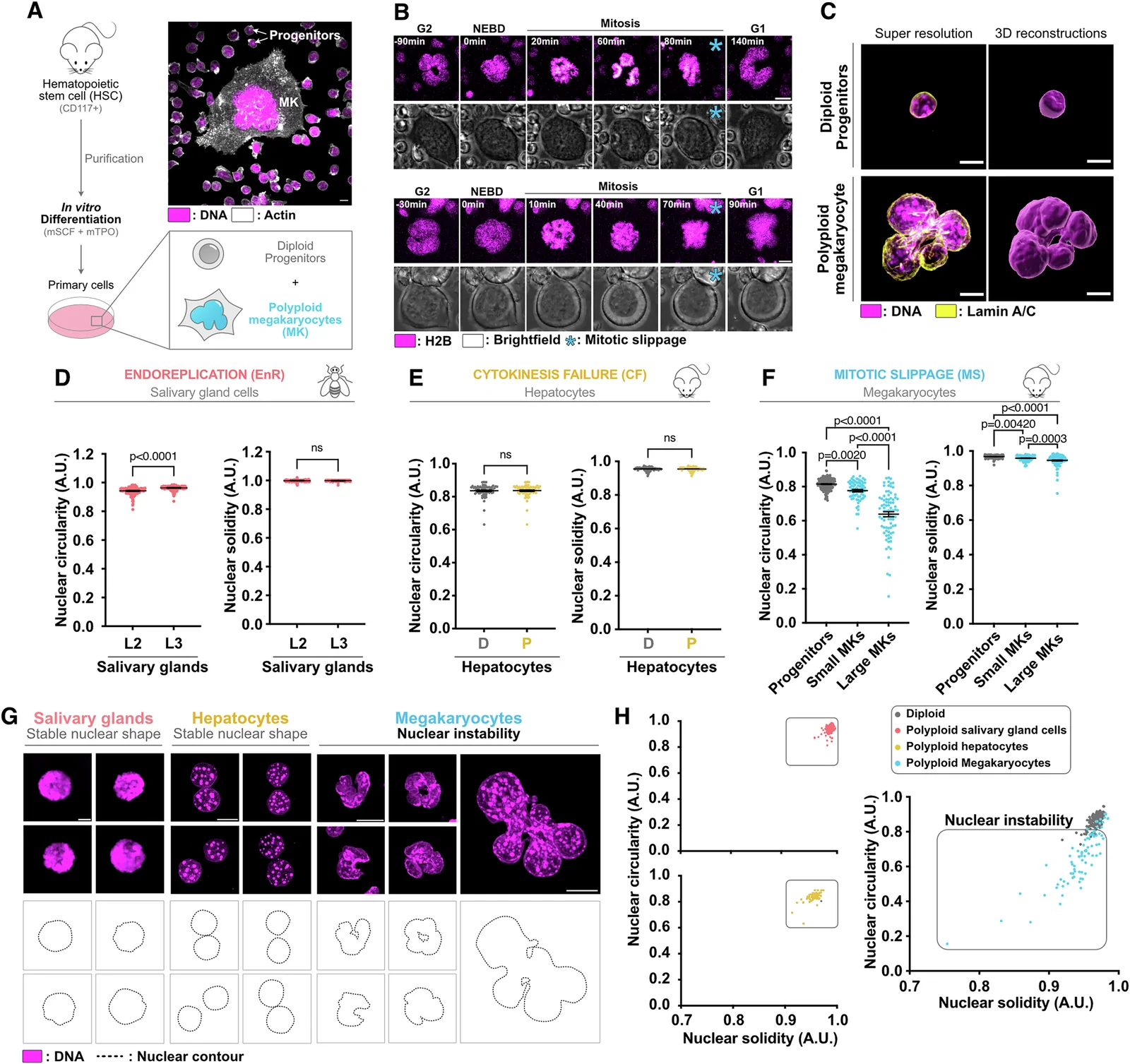
Megakaryocytes undergo mitotic slippage and display nuclear instability. (**A**) Left panel—schematic workflow showing the methods used to induce megakaryocyte (MK) differentiation in vitro. Right panel— representative image showing a large polyploid megakaryocyte surrounded by diploid progenitors. Actin in grey, DNA in magenta. (**B**) Stills from time-lapse imaging of MKs stably expressing mCherry-H2B (in magenta). Brightfield in grey. (**C**) SIM representative images and corresponding 3D reconstructions showing diploid progenitors and polyploid megakaryocytes. Lamin A/C in yellow, DNA in magenta. (**D**–**F**) Graphs showing nuclear circularity and solidity in (**D**) *Drosophila* salivary glands (in red), in (**E**) mouse diploid (in grey) or polyploid hepatocytes (in yellow) and in (**F**) diploid mouse progenitors (in grey) and in polyploid megakaryocytes (in blue). Mean ± SEM, (**D**) >200, (**E**) >65, (**F**) >65 interphase cells analysed per condition, three independent experiments. (**G**) Representative images of megakaryocyte, hepatocyte or salivary gland nuclei, the nuclear contour is indicated below. DNA in magenta. (**H**) Graph showing the nuclear circularity and solidity index from (**D**–**F**) in *Drosophila* salivary glands (in red), in mouse diploid (in grey) or polyploid hepatocytes (in yellow) and in diploid mouse progenitors (in grey) and in polyploid megakaryocytes (in blue). Scale bars: 10 µm. MS mitotic slippage, EnR endoreplication, CF cytokinesis failure, MK megakaryocytes, NEBD nuclear envelope breakdown, AU arbitrary unit. (**D**, **E**) *t*-test (two-sided). (**F**) ANOVA test (one-sided). ns not significant. Source data are available online for this figure.

**Figure EV4:**
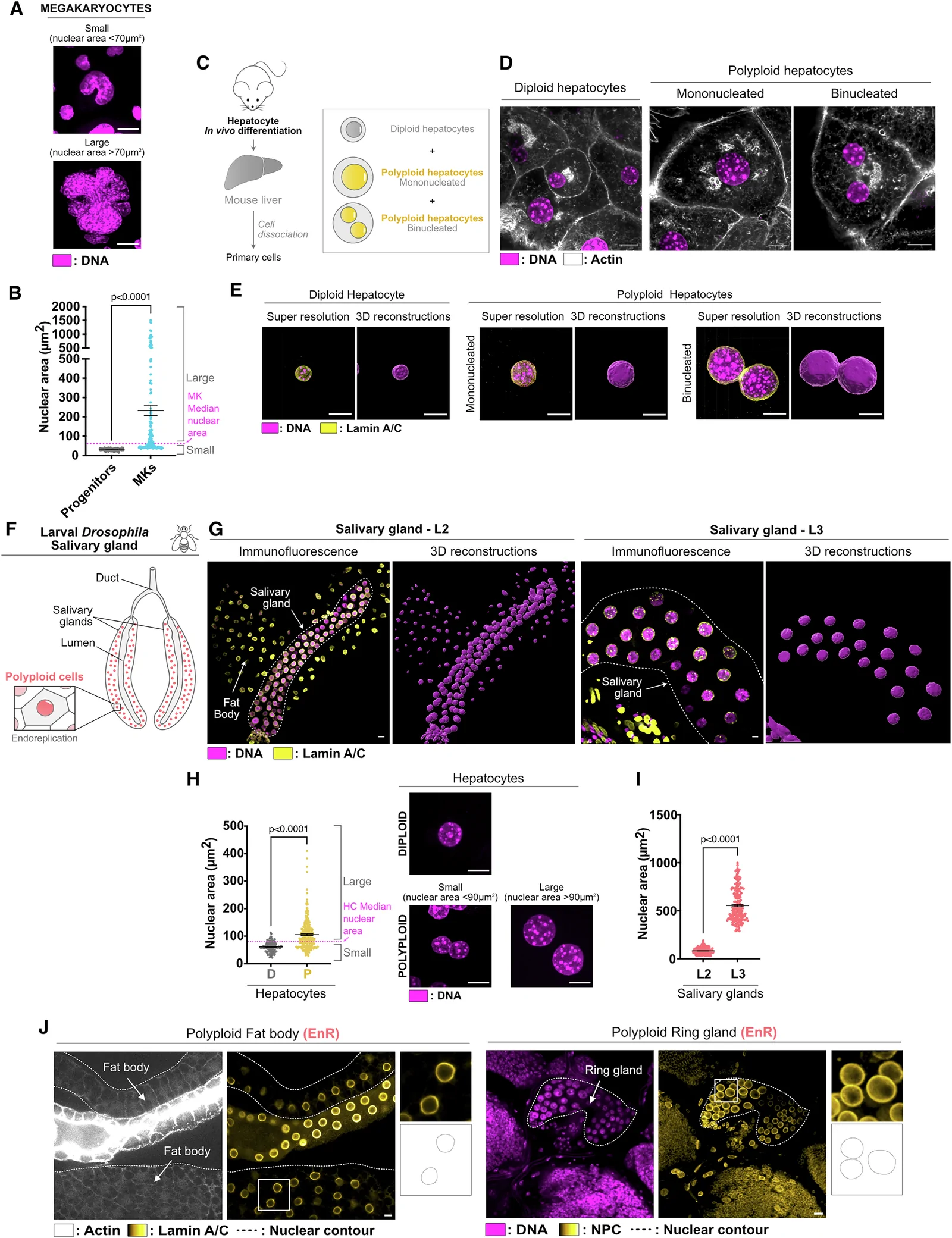
Physiological polyploid cells generated by cytokinesis failure and endoreplication show homogenous nuclear shape. (**A**) Representative images of small and large MKs. DNA in magenta. (**B**) Graph showing nuclear area in diploid progenitors and in polyploid MKs. The MK median nuclear area was used to distinguish small and large MKs. Mean ± SEM, >150 interphase cells analysed per condition, three independent experiments. (**C**) Schematic workflow showing the methods used to obtain mouse hepatocytes. (**D**) Representative images showing diploid and polyploid hepatocytes. Actin in grey, DNA in magenta. (**E**) SIM representative images and their corresponding 3D reconstructions showing diploid and polyploid hepatocytes. Lamin A/C in yellow, DNA in magenta. (**F**) Schematic representation of *Drosophila* salivary glands. (**G**) Representative images and their corresponding 3D reconstructions showing polyploid salivary glands. Lamin A/C in yellow, DNA in magenta. (**H**) Left panel—Graph showing the nuclear area in diploid and in polyploid hepatocytes (in grey and yellow, respectively). The hepatocyte median nuclear area was used to distinguish small and large hepatocytes. Mean ± SEM, >140 interphase cells analysed per condition, three independent experiments. Right panel—Representative images showing diploid and polyploid hepatocytes. DNA in magenta. (**I**) Graph showing the nuclear area in polyploid salivary glands at two different developmental stages (L2 and L3). Mean ± SEM, >100 cells analysed, three independent experiments. (**J**) Representative immunofluorescence images showing *Drosophila* fat body (left panel) and ring gland (right panel). Lamin A/C or Nuclear pore complex (NPC) in yellow hot, Actin in grey, DNA in magenta. Scale bars: 10 µm. D diploid. P polyploid, MK megakaryocyte, NPC nuclear pore complex. (**B**,** H**,** I**) *t*-test (two-sided). ns not significant.

We decided to determine whether physiological polyploid cells generated by MS also exhibit nuclear deformations. To do so, we analysed megakaryocytes, differentiated polyploid cells, derived from hematopoietic progenitors (Mazzi et al, 2018), and responsible for the production of RNAs and proteins that will be transported to the cell tips to generate anucleate platelets (Carminita et al, 2024). To obtain megakaryocytes, we isolated mouse diploid hematopoietic stem cells and induced their differentiation in vitro using a cocktail of cytokines (Fig. 6A). We obtained a population of primary cells composed of diploid hematopoietic stem cells/progenitors and polyploid megakaryocytes showing different cell and nuclear sizes. These differences in size reflect a ploidy continuum and allow us to identify polyploid cells (Fig. EV4A,B). In contrast to RPE-1 cells, we could not analyze the first cell cycle upon WGD due to technical reasons; instead, we analyzed megakaryocytes at different ploidy levels. Using live imaging approaches of megakaryocytes isolated from mice expressing H2B-mCherry, we observed that nuclei exit mitosis without chromosome segregation or following limited movement during anaphase A. These lack any sign of cytokinesis furrowing, confirming MS (Fig. 6B; Expanded view Movies 9 and 10), as previously suggested (Mazzi et al, 2018; Trakala et al, 2015).

In our hands, and in agreement with in vivo analysis (Mazzi et al, 2018; Penington and Streatfield, 1975), megakaryocytes displayed a single multilobulated, highly deformed nucleus (Fig. 6C). Quantification of circularity and solidity revealed a decrease in a ploidy-dependent manner (Fig. 6D–F). Similar to MS-induced RPE-1 cells, we observed that megakaryocyte nuclear shapes were highly heterogenous (Fig. 6G,H). This reflects that megakaryocyte polyploidization by MS also generates nuclear instability. When comparing with other physiological polyploid cells, we noticed that nuclear instability was specific to megakaryocytes. Indeed, analysis of polyploid hepatocytes isolated from mouse liver, that become polyploid by CF (Davoli and de Lange, 2011; Gentric and Desdouets, 2015) or *Drosophila* salivary glands, that become polyploid by EnR (Frawley and Orr-Weaver, 2015; Zielke et al, 2011), showed homogeneous circular nuclei lacking nuclear deformations (Figs. 6D–H and EV4C–I). Similar observations were made in vivo in two other polyploid *Drosophila* tissues—the fat body and the ring gland that become polyploid by EnR (Orr-Weaver, 2015) (Fig. EV4J).

Together, these findings show that MS, but not CF or EnR, impacts nuclear shape and stability in physiological polyploid settings.

### Mitotic slippage in megakaryocytes is associated with changes in chromatin compaction and nuclear envelope composition

Our results in RPE-1 cells show that MS-induced tetraploid cells exhibit changes in chromatin compaction and nuclear envelope composition, promoting nuclear softness and deformability, facilitating nuclear deformations by the microtubules. We therefore considered the possibility that megakaryocytes undergo a similar process.

**Figure 7:**
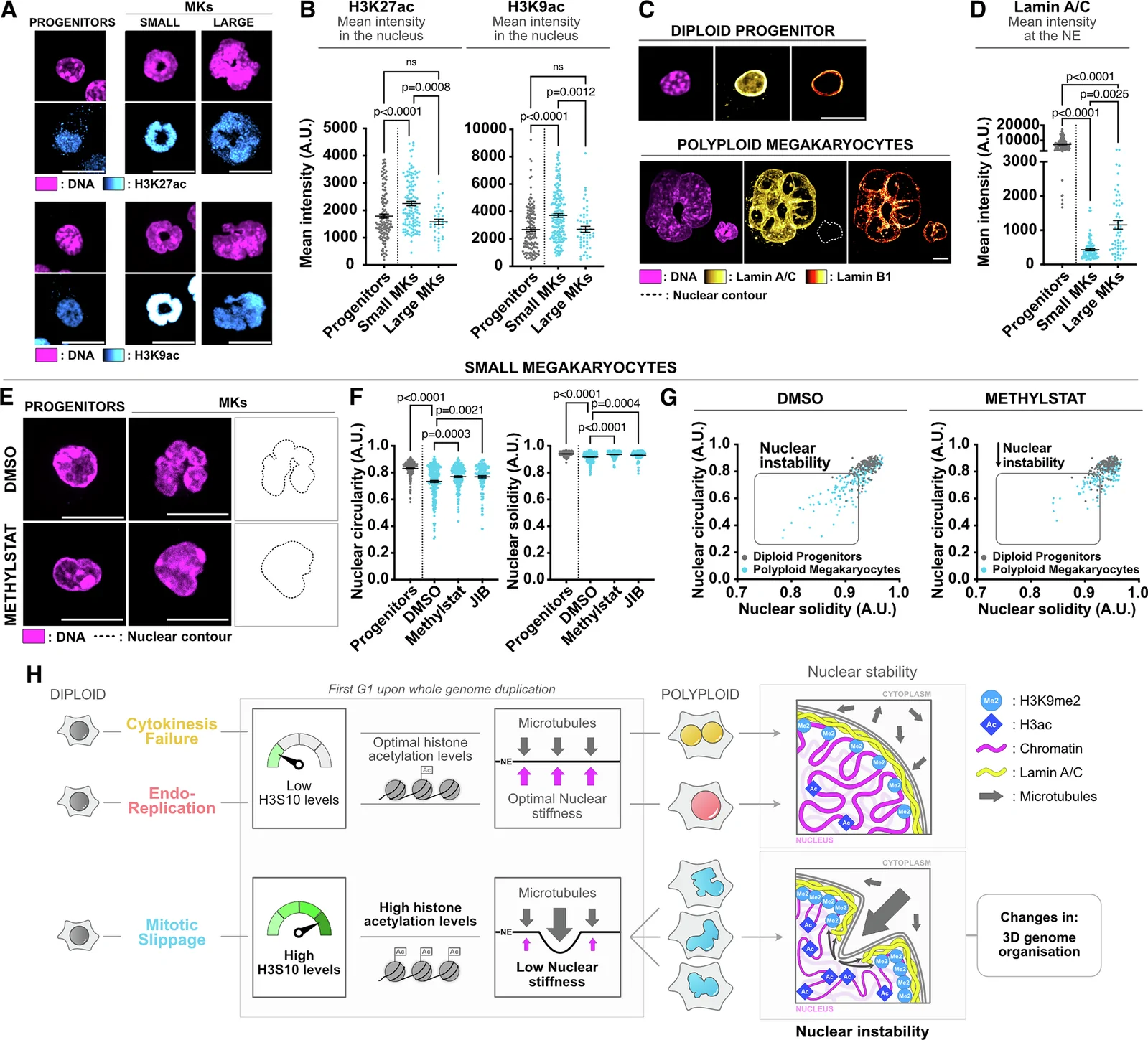
Mitotic slippage in megakaryocytes alters chromatin and nuclear envelope composition, promoting nuclear deformation. (**A**) Representative images of mouse diploid progenitors and polyploid megakaryocytes. DNA in magenta. H3K27ac (upper panel) and H3K9ac (lower panel) in cyan hot. (**B**) Graph showing the H3K27ac (left panel) and H3K9ac (right panel) levels in diploid (in grey) and in polyploid megakaryocytes (in blue). Mean ± SEM, >140 interphase cells analysed per condition, three independent experiments. (**C**) Representative images of diploid progenitor and polyploid megakaryocytes. Lamin A/C in yellow hot, Lamin B1 in red hot and DNA in magenta. The dotted line shows the nuclear contour. (**D**) Lamin A/C mean intensity at the nuclear envelope in diploid (in grey) and in polyploid megakaryocytes (in blue). Mean ± SEM, >150 interphase cells analysed per condition, three independent experiments. (**E**) Representative images of diploid progenitors and polyploid megakaryocytes treated with DMSO or Methylstat. DNA in magenta. The dotted line shows the nuclear contour. (**F**) Graph showing the nuclear circularity and solidity indices in diploid progenitors (in grey) and polyploid megakaryocytes (in blue) treated with DMSO or 2 µM Methylstat or 2.5 µM JIB. Mean ± SEM, >90 interphase cells analysed per condition, three independent experiments. (**G**) Graph showing the nuclear circularity and solidity index from (**F**) in diploid progenitors (in grey) and polyploid megakaryocytes (in blue) treated with DMSO or 2 µM Methylstat. The same control (diploid progenitors) was used for the two graphs. (**H**) MS, unlike CF and EnR, generated tetraploid cells displaying abnormal nuclear configurations. These defects include maintenance of high H3S10 phosphorylation levels in G1, which promotes histone acetylation and thus decreases nuclear stiffness. Scale bars: 10 µm. MK megakaryocyte, NE nuclear envelope, AU arbitrary unit. (**B**,** D**,** F**) ANOVA test (one-sided). ns not significant. Source data are available online for this figure.

**Figure EV5:**
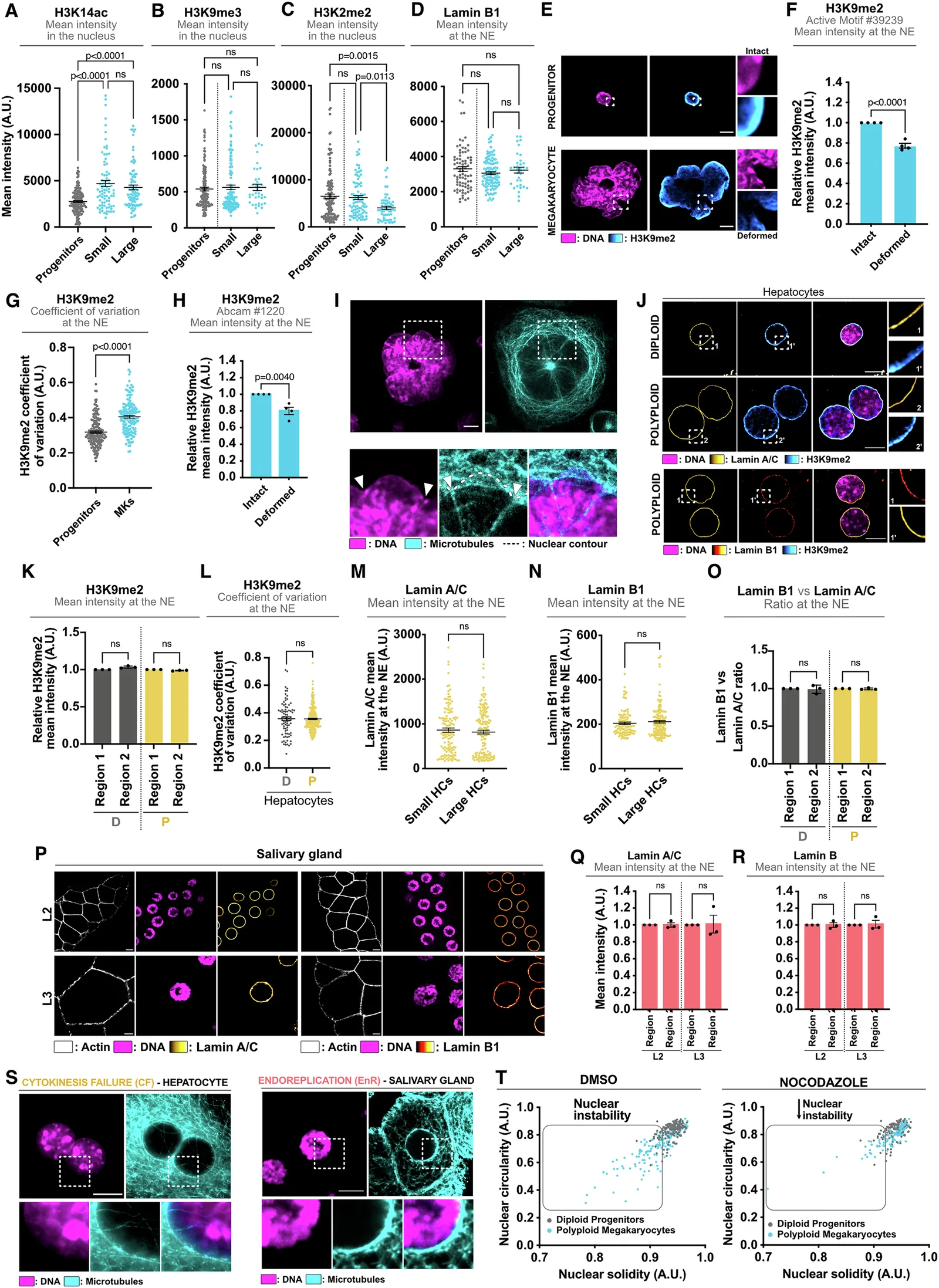
Polyploid hepatocytes and salivary gland cells do not show defects in H3K9me2 and Lamin A/C distribution. (**A**–**D**) Graphs showing (**A**) H3K27ac, (**B**) H3K9me3 and (**C**) H3K9me2 mean intensity in the nucleus in diploid progenitors (in grey) and in polyploid megakaryocytes (in blue). Mean ± SEM, >120 cells analysed, three independent experiments and Lamin B1 mean intensity at the nuclear envelope (**D**) in diploid progenitors (in grey) and in polyploid megakaryocytes (in blue). (**E**) SIM representative images of diploid progenitors and polyploid megakaryocytes. H3K9me2 in cyan hot, DNA in magenta. (**F**,** G**) Graphs showing H3K9me2 (**F**) mean intensity and (**G**) coefficient of variation (defined as the ratio of the standard deviation to the mean) in progenitors (in grey) and in MKs (in blue). Mean ± SEM, >150 interphase cells analysed per condition, four independent experiments. (**H**) Graph showing H3K9me2 mean intensity in progenitors (in grey) and in MKs (in blue). Mean ± SEM, >65 interphase cells analysed per condition, four independent experiments. (**I**) Representative images of microtubules (in cyan) in MKs, DNA in magenta. (**J**) SIM representative images showing diploid and polyploid hepatocytes. Lamin A/C, Lamin B1 and H3K9me2 in yellow hot, red hot and cyan hot, respectively, DNA in magenta. (**K**, **L**) Graphs showing (**K**) the relative H3K9me2 mean intensity at the NE and (**L**) the H3K9me2 coefficient of variation (defined as the ratio of the standard deviation to the mean) at the NE in diploid (in grey) and in polyploid (in yellow) hepatocytes. Mean ± SEM, >90 interphase cells analysed per condition, three independent experiments. (**M**–**O**) Graphs presenting (**M**) Lamin A/C, (**N**) and Lamin B1 mean intensity at the NE and (**O**) the Lamin B1 vs Lamin A/C ratio at the NE in hepatocytes. Mean ± SEM, >90 interphase cells analysed per condition, three independent experiments. (**P**) Representative images showing *Drosophila* salivary glands at two different developmental stages (L2 and L3). Lamin A/C and Lamin B1 in yellow hot and red hot, respectively, Actin in grey, DNA in magenta. (**Q**, **R**) Graphs showing (**Q**) Lamin A/C and (**R**) Lamin B1 mean intensity at the NE in *Drosophila* salivary glands. Mean ± SEM, >150 cells analysed per condition, three independent experiments. (**S**) Representative images showing (left panel) polyploid hepatocytes and (right panel) *Drosophila* salivary gland cells. Microtubules in cyan. DNA in magenta. (**T**) Graph presenting the nuclear circularity and solidity index in progenitors (in grey) or megakaryocytes (in blue) treated with DMSO or Nocodazole. Scale bars: 10 µm. D diploid, P polyploid, HC hepatocytes, MK megakaryocytes, NE nuclear envelope. (**D**) ANOVA test (one-sided). (**F**,** G**,** J**–**O**,** Q**,** R**) *t*-test (two-sided). ns not significant.

We first tested whether histone compaction was altered upon MS in megakaryocytes. We found an increase in H3K27ac and H3K9ac levels in small megakaryocytes (Fig. 7A,B) while H3K14ac levels were increased in both small and large megakaryocytes (Fig. EV5A). We also observed a global decrease in H3K9me2 levels with megakaryocyte ploidy while H3K9me3 levels remained comparable between progenitors and megakaryocytes (Fig. EV5B,C). Interestingly, a local decrease in H3K9me2 levels in deformed regions was also found in megakaryocytes using two different antibodies (Fig. EV5E–H). Concerning the nuclear envelope composition, a striking switch in Lamina composition during megakaryocyte differentiation was observed. Small and large megakaryocytes displayed lower levels of Lamin A/C than diploid progenitors, while Lamin B1 levels remained largely unchanged (Figs. 7C,D and EV5D). In contrast, defects in H3K9me2 distribution and Lamina composition in hepatocytes or in the Lamina network in salivary gland cells were not detected (Fig. EV5K–R). The high levels of histone acetylation and the decrease in Lamin A/C levels in megakaryocytes—two phenotypes associated with softer nuclei (Miroshnikova and Wickström, 2022)—are consistent with previous data showing that nuclear stiffness decreased during megakaryocyte differentiation (Shin et al, 2013). To confirm that low nuclear stiffness contributes to nuclear deformations in megakaryocytes, we promoted nuclear compaction by treating cells with Methylstat or JIB-04 using a similar strategy as described above for RPE-1 cells. This was sufficient to alter nuclear shape and to reduce nuclear instability in megakaryocytes (Fig. 7E–G). Together, these findings suggest that during differentiation, megakaryocytes are in a state in which nuclear resistance is low—due to changes in chromatin compaction and nuclear envelope composition—favouring nuclear deformability. We next investigated whether the microtubule cytoskeleton also contributes to the nuclear deformations in megakaryocytes. We found thick microtubule bundles positioned around nuclear invaginations (Fig. EV5I), which were absent in hepatocytes or salivary glands. In these former examples, the microtubule cytoskeleton was organised around the nucleus, forming a microtubule ring (Fig. EV5S). Importantly, removing microtubules was sufficient to decrease the nuclear instability in megakaryocytes (Fig. EV5T), similarly to what was observed in RPE-1 MS-induced tetraploid cells.

These results suggest that highly specialised and differentiated cells, such as polyploid megakaryocytes, rely on low nuclear stiffness to generate cells with irregular nuclear architecture in vivo. Importantly, our findings also identified the molecular mechanism underlying the irregular nuclear shape in megakaryocytes, initially described 50 years ago (Penington and Streatfield, 1975).

## Discussion

In this study, we show that different routes to WGD lead to different outcomes. Indeed, MS, unlike CF and EnR, generated tetraploid cells displaying abnormal nuclear configurations. These defects include maintenance of high H3S10 phosphorylation levels in G1, which seem to promote histone acetylation and thus decreased nuclear stiffness. Due to the increased nuclear deformability, cytoplasmic microtubules generate nuclear invaginations that induce local changes in the chromatin/nuclear envelope composition. These findings are supported by other studies, which have shown that microtubules, Lamins and peripheral heterochromatin also contribute to nuclear morphology in diploid cells (Stephens et al, 2019; Biedzinski et al, 2020; Tariq et al, 2017; Nava et al, 2020). Together, these defects produce a heterogenous population of polyploid nuclei with various shapes—that we defined as nuclear instability—culminating with changes in 3D genome organisation (Fig. 7H).

Importantly, we observed these defects in the first G1 upon MS, contributing to a better understanding of the early consequences of WGD. However, while the extent of the nuclear deformations is visually impressive, the effects on chromatin accessibility and on 3D genome organisation remain mild, even if significant, in the first G1 upon MS. This suggests that tetraploid cells can, at least in part, deal with these defects. Nevertheless, we cannot exclude that these early changes may be amplified after subsequent cell cycles.

MS, CF and EnR have been proposed to be at the origin of WGD in tumours (Davoli and de Lange, 2011; Ganem et al, 2007; Zeng et al, 2023), but little is known about their proportion and respective roles in cancer. Nuclear deformations were observed in tumours since the mid-19th century and are even used for diagnosis in certain situations (de Las Heras and Schirmer, 2014), but a potential link with WGD and MS was never established. It remains to be understood whether nuclear deformations and instability, typical of MS, contribute to the tumorigenic process. MS, CF and EnR are also involved in developmental programmes generating physiological polyploid cells (Frawley and Orr-Weaver, 2015; Mazzi et al, 2018; Gentric and Desdouets, 2015; Girdler and Röper, 2014). Whether these different strategies reflect a particular advantage or are just byproducts of selective pressure remains unknown.

Interestingly, to our knowledge, megakaryocytes are the only example of MS-generated physiological polyploid cells, making them quite unique. By showing that megakaryocytes display changes in chromatin/ nuclear envelope composition that contribute to nuclear deformations, we exposed a molecular mechanism underlying the generation of megakaryocytes with irregular nuclear architecture. It has been shown that megakaryocyte reprogramming to induce polyploidy through EnR instead of MS contributes to prevent nuclear deformations (Trakala et al, 2015), confirming the predominant role of MS in generating megakaryocytes with irregular architecture. The global decrease in chromatin compaction observed upon MS may promote transcription during megakaryocyte differentiation, thereby sustaining the massive production of RNAs that are ultimately encapsulated in platelets (De Wispelaere and Freson, 2022). Alternatively, the MS-induced decrease in nuclear stiffness may facilitate megakaryocyte migration through the bone marrow toward blood vessels (Stegner et al, 2017). These hypotheses should be further investigated to better understand how nuclear deformations contribute to megakaryocyte function.

Overall, our findings show that the cell cycle route chosen to generate polyploid cells has different outcomes. More importantly, our results highlight the fact that in physiological and pathological conditions, different methods to induce WGD have the potential to generate polyploid cells with specific features contributing to their inherent function.

## Methods

**Table:** Reagents and tools table

Reagent/resource	Reference or source	Identifier or catalogue number
**Experimental models**		
BJ cell line/male	ATCC	Cat# CRL-4001, RRID:CVCL_6573
HCT116 cell line/male	ATCC	Cat# CCL-247, RRID:CVCL_0291
HEK293T cell line/female	ATCC	Cat# CRL-1573, RRID:CVCL_0045
*Mus musculus* C57Bl6/N	Charles River (France)	N/A
*Mus musculus* C57Bl6/N R26-H2B-mCherry transgenic line	Curie Institute SPF inbred from Abe et al, Genesis, 2011	N/A
RPE-1 cell line/female	ATCC	Cat# CRL-4000, RRID:CVCL_4388
w[1118] flies	Bloomington	#3605
**Recombinant DNA**		
GFP-H3 WT	From Poleshko et al, 2019	N/A
GFP-H3S10A	From Poleshko et al, 2019	N/A
**Antibodies**		
Alexa Fluor™ Plus 647 Phalloidin	Thermo Fisher	A30107
Goat anti-Mouse IgG1 Cross-Adsorbed Secondary Antibody, Alexa Fluor™ 488	Thermo Fisher	A-21121, RRID:AB_2535764
Goat anti-Mouse IgG2a Cross-Adsorbed Secondary Antibody, Alexa Fluor™ 546	Thermo Fisher	A-21133, RRID:AB_2535772
Goat anti-Mouse IgG2b Cross-Adsorbed Secondary Antibody, Alexa Fluor™ 488	Thermo Fisher	A-21141, RRID:AB_2535778
goat anti-Rabbit IgG (H + L) Highly Cross-Adsorbed Secondary Antibody, Alexa Fluor 647	Thermo Fisher	A21245, RRID:AB_2535813
Goat anti-Rabbit IgG (H + L) Highly Cross-Adsorbed Secondary Antibody, Alexa Fluor 546	Thermo Fisher	A-11035, RRID:AB_2534093
Mouse αTubulin (DM1α)	Sigma-Aldrich	T9026, RRID:AB_477593
Mouse H3K27ac	Active Motif	39685, RRID:AB_2793305
Mouse H3K9me2	Abcam	ab1220; RRID:AB_449854
Mouse H3S10	Cell Signalling	9706, RRID:AB_331748
Mouse Lamin A/C	Sigma-Aldrich	SAB4200236, RRID:AB_10743057
Mouse Lamin A/C	DHSB	ADL101, RRID:AB_528332
Mouse Lamin B	DHSB	ADL67.10, RRID:AB_528336
Mouse Lap2β	BD Bioscience	611000, RRID:AB_398313
Mouse LBR	Abcam	ab232731, RRID:AB_3246426
Mouse Nuclear pore complex	Abcam	ab24609, RRID:AB_448181
Rabbit Emerin	ProteinTech	10351-1-AP, RRID:AB_2100056
Rabbit H3K14ac	Abcam	ab52946, RRID:AB_880442
Rabbit H3K27me3	Active motif	39155, RRID:AB_2561020
Rabbit H3K9ac	Ozyme	9649S; RRID:AB_823528
Rabbit H3K9me2	Active Motif	39239, RRID:AB_2793199
Rabbit H3K9me3	Abcam	ab8898, RRID:AB_306848
Rabbit HP1α	Abcam	ab109028, RRID:AB_10858495
Rabbit Lamin B1	Abcam	ab16048, RRID:AB_443298
Rabbit β-catenin	Sigma-Aldrich	C2206, RRID:AB_476831
**Oligonucleotides and other sequence-based reagents**		
**Chemicals, enzymes and other reagents**		
Accutase	Sigma	A6964
Anti-Anti	Gibco	11570486
Antibiotic-antimycotic (100X)	Gibco	15240-062
Asunaprevir	Selleckchem	S4935
Auxin	Sigma-Aldrich	I5148
Blebbistatin	Sigma-Aldrich	B0560
BSA	Euromedex	04-100-812-C
C646	MedChem	HY-13823
Calyculin A	MedChem	HY-18983
DAPI (4’,6-diamidino-2-phenylindole)	Sigma-Aldrich	D8417
DMSO anhydrous	Sigma	276855
Doxycycline	Sigma-Aldrich	D3447
Dulbecco’s modified medium (DMEM) F12	Gibco	11320-033
EDTA	Invitrogen	10135423
Fibronectin	Sigma	F1141
G418	Roche	4727878001
Glycerol	24388-295	VWR
Hoechst 33342	Sigma-Aldrich	94403
Isoflurane	Baxter	DDG9621
JIB-04	MedChem	HY-13953
Methylstat	MedChem	HY-15221
MetOH	Honeywell	32213
Monastrol	Selleckchem	S8439
MPI-0479605	Selleckchem	S7488
mSCF	Peprotech	250-03
mTPO	Peprotech	315-14
n-propyl gallate	Sigma-Aldrich	P3130
Nocodazole	MedChem	HY-13520
Normal goat serum	Gibco	10098792
Palbociclib	Selleckchem	S1579
Paraformaldehyde	Electron Microscopy Sciences	15710
Penicillin-streptomycin	Gibco	15070-063
Poly-D-lysine hydrobromide	Sigma	P6407
Rapiclear 1.49	Sunjin Lab	RC149002
RO-3306	MedChem	HY-12529
RPMI 1640	Gibco	11530586
SP600125	MedChem	HY-12041
Taxol	Sigma-Aldrich	T1912
Triton X-100	Euromedex	2000-C
**Software**		
GraphPad Prism	www.graphpad.com	Version 10 for Mac RRID:SCR_002798
ImageJ	https://imagej.net/software/fiji/downloads	V2.1.0/1.53c
Metamorph	https://www.moleculardevices.com/products/cellular-imaging-systems/high-content-analysis/metamorph-microscopy	7.10.1 Molecular Devices RRID:SCR_002368
BD FACS DIVA	https://www.bdbiosciences.com/en-us/products/software/instrument-software/bd-facsdiva-software	Version 8.0.1
**Other**		
Illumina HiSeq X platform, PE150 configuration	Illumina	N/A
NovaSeq X Plus instrument	Illumina	N/A

### Human cell culture and generation of cell lines

#### Cell culture

Cells were maintained at 37 °C in a 5% CO_2_ atmosphere. hTERT RPE-1 cells (female, ATCC Cat# CRL-4000, RRID:CVCL_4388), HEK293T cells (female, ATCC Cat# CRL-1573, RRID:CVCL_0045), BJ cells (male, ATCC Cat# CRL-4001, RRID:CVCL_6573) and HCT116 cells (male, ATCC Cat# CCL-247, RRID:CVCL_0291) were grown in Dulbecco’s modified medium (DMEM) F12 (11320-033 from Gibco) containing 10% foetal bovine serum (GE Healthcare), 100 U/ml penicillin, 100 U/ml streptomycin (15140-122 from Gibco).

All cells were routinely checked and tested negative for mycoplasma infection. Identity and purity of the human cell lines used in this study were tested and confirmed using STR authentication.

#### Generation of RPE-1 GFP-H3 WT and GFP-H3S10A stable cell lines

RPE-1 cells were transfected with 10 µg GFP-H3 WT and GFP-H3S10A (Poleshko et al, 2019) using the JET PRIME kit (Polyplus Transfection, 114-07) according to the manufacturer’s protocol. After 24 h, 500 µg/ml G418 (4727878001 from Roche) was added to the cell culture medium. After 72 h of antibiotic selection, GFP-positive cells were then collected using Sony SH800 FACS (BD FACSDiva Software Version 8.0.1).

GFP-H3 WT and GFP-H3S10A plasmids were a gift from Epstein JA (Poleshko et al, 2019).

### Transgenic animals

For animal care, we followed the European and French National Regulation for the Protection of Vertebrate Animals used for Experimental and other Scientific Purposes (Directive 2010/63; French Decree 2013-118). The project was authorised and benefited from the guidance of the Animal Welfare Body, Research Centre, Curie Institute. All mice were kept in the Curie Institute Specific Pathogen Free (SPF) animal facility for breeding. The transgenic R26-H2B-mCherry mouse line (Abe et al, 2011) was back-crossed in our animal facility on a C57Bl6/N genetic background (Charles River, France). Only heterozygous R26-H2B-mCherry +/− and control littermate males between 3 and 6 months of age were included in the analysis. Animals were anaesthetised by inhalation of isoflurane (Baxter, DDG9621) before being sacrificed by cervical dislocation for bone tissue collection.

### Mouse megakaryocyte in vitro differentiation and cell culture

#### Hematopoietic progenitor enrichment

Tibias and femurs from 2- to 6-month-old male mice were dissected and excess tissue was removed using scalpels. One epiphyse of each bone was cut ant the opened bone was placed into a 1.5 mL centrifugation tube containing a 200-uL tip. The bones were then centrifuged at high speed for 10 s in an Eppendorf tube to eject the bone marrow cells. The cells were resuspended in 1 ml of cold RPMI 1640 (11530586 from Gibco) containing 10% foetal bovine serum (GE Healthcare), 100 U/ml penicillin, 100 U/ml streptomycin (15140-122 from Gibco), Anti-Anti (11570486 from Gibco)—hereafter called complete RPMI medium—and centrifuged for 5 min at 1700 rpm, 4 °C.

About 125 µl of cold complete RPMI medium were used to resuspend the cells, then 25 µl of anti-CD117 magnetic beads (130-097-146 from Miltenyi Biotec) were added and mix to the cells. Cells were incubated on ice for 30 min with the beads. Then, 1 ml of cold complete RPMI medium was added to the cells, and they were filtered through a 100-µm cell strainer (431752 from Coning) into 50 ml tubes. The tubes and the cell strainers were washed with an additional 1 ml of cold complete RPMI medium. Cells were centrifuged for 5 min at 1700 rpm, 4 °C. During the centrifugation, an MS column (130-042-201 from Miltenyi Biotec) was prewet with 2 ml of cold complete RPMI medium and installed on MACS magnet (130-042-501 from Miltenyi Biotec). The cells were then resuspended in 1 ml of cold complete RPMI medium and transferred to a 2 ml tube. Using an insulin syringe fitted with a short 25 G needle (300400 from BD Bioscience), cells were transferred to the column. After, the tubes and the columns were washed using 2 ml of cold complete RPMI medium. The CD117-positive fraction was then collected in a tube by pouring the cells using a plunger. The CD117-positive cells were centrifuged for 5 min at 1700 rpm, 4 °C and resuspended in 500 µl of cold complete RPMI medium.

#### Cell culture and in vitro differentiation

Purified CD117-positive cells were cultured on coverslips pre-treated with PolyLysine (0.02 mg/ml during 5’, 37 °C; HY-126437B from MedChemExpress) and Fibronectin (20 µL in 500 µl PBS, ON 37 °C; F1141-5MG from Sigma-Aldrich) in DMEM-F12 + 10% foetal bovine serum (GE Healthcare) + 100 U/ml penicillin, 100 U/ml streptomycin (15140-122 from Gibco). Cells were maintained at 37 °C in a 5% CO_2_ atmosphere. Megakaryocyte differentiation was induced by adding mSCF (50 ng/mL; 250-03 from Peprotech) + mTPO (50 ng/mL; 315-14 from Peprotech) for at least 3 days.

### Isolation of mouse Hepatocytes

Two old C57BL/6J mice were anesthetized with an intra-peritoneal injection of 10 mg/kg xylazine and 100 mg/kg ketamine. Primary hepatocytes were isolated by the two-step liver collagenase perfusion method and seeded in complete medium, as described previously (Donne et al, 2022). Briefly, after caudal vena cava catheterisation and portal vein opening, livers were washed with 50 mL of perfusion solution containing 10 mM 4-(2-hydroxyethyl)-1-piperazineethanesulfonic acid (HEPES, Merck #H6147), 137 mM NaCl (Merck #5886), 2.68 mM KCl (Merck #P5405), 232 µM Na_2_HPO_4_ (Merck #S5136), 0.5 mM ethylenediaminetetraacetic acid (EDTA, Thermo Fisher Scientific #15575020) dissolved in deionized sterile water. Then, livers were digested with a collagenase solution containing 0.5 mg/mL of collagenase type IV (from *Clostridium histolyticum*, Merck #C5138) diluted in perfusion solution, and 10 mM of CaCl_2_ (Merck #C7902). After isolation, hepatocytes were collected, washed, and resuspended in complete medium: William’s E medium GlutaMAX (Thermo Fisher Scientific #32551020) supplemented with bovine serum albumin 0,1% (Merck #A8412), penicillin-streptomycin 100 U/mL (Thermo Fisher Scientific #15140122), fungizone B 100 µg/mL (Thermo Fisher Scientific #15290026), dexamethasone 25 nM (Merck #D2915) and supplemented with 10% decomplemented foetal bovine serum (FBS, Thermo Fisher Scientific #10270106). The cell viability was equivalent in all groups (≥85%) as assessed by the Trypan blue exclusion method. Hepatocytes were seeded at a density of 6.0e + 5 in 6-cm dishes and incubated at 37 °C under an atmosphere containing 5% (v/v) CO_2_, for 4 h, to allow the cells to adhere. The medium was then replaced with fresh FBS-free complete medium, which remained unchanged for the 24 h of primary culture.

### Fly husbandry and fly stocks

Flies were raised on cornmeal medium (0.75% agar, 3.5% organic wheat flour, 5.0% yeast, 5.5% sugar, 2.5% nipagin, 1.0% penicillin-streptomycin and 0.4% propionic acid). Fly stocks were maintained at 18 °C. Stocks used in this study: w[1118] (3605 from Bloomington).

In all experiments, larvae were staged to obtain comparable stages of development. Egg collection was performed at 25 °C for 24 h. After development at 25 °C, third instar larvae were used for dissection.

### Treatments

#### Chemicals

The drugs were used at the following concentrations: Auxin (I5148 from Sigma-Aldrich), 500 µM; Doxycycline (D3447 from Sigma-Aldrich), 2 µg/ml; Asunaprevir (S4935 from Selleckchem), 3 µM; Monastrol (S8439 from Selleckchem), 50 µM; MPI-0479605 (S7488 from Selleckchem), 1 µM; Blebbistatin (B0560 from Sigma- Aldrich), 20 µM; Palbociclib (S1579 from Selleckchem), 1 µM; Methylstat (HY-15221 from Medchem), 2 (for live imaging or megakaryocytes) or 4 µM (for fixed cells); JIB-04 (HY-13953 from MedChemExpress), 2,5 (for megakaryocytes) or 5 µM (for human cell lines), Nocodazole (HY-13520 from Medchem), 2 µM; Taxol (T1912 from Sigma-Aldrich), 1 nM; SP600125 (HY-12041 from Medchem), 20 µM; RO-3306 (HY-12529 from Medchem), 10 µM; Calyculin A (HY-18983 from Medchem), 5 nM; C646 (HY-13823 from Medchem), 30 µM.

Methylstat, JIB and C646 were added 6 h before the induction of whole genome duplication. Nocodazole and Taxol were added 2 h before the induction of whole genome duplication.

Cells were treated with Calyculin A for 2:5 h before fixation.

Megakaryocytes were incubated with Methylstat and JIB for 6 h, 3 days after the addition of the cytokines. To analyze megakaryocytes that performed mitotic slippage in the presence of the drugs, only the small megakaryocytes were considered.

#### Induction of WGD in human cell lines

To induce mitotic slippage, cells were incubated with DMSO (276855 from Sigma-Aldrich) or with a combination of 50 µM Monastrol (S8439 from Selleckchem) + 1 µM MPI-0479605 (S7488 from Selleckchem) for at least 4 h. After 24 h of treatment, we got a mixed population composed of 40 ± 3% of diploid cells and 60 ± 3% of tetraploid cells.

Alternatively, to induce mitotic slippage, CCNB1 depletion in RPE CCNB1^AID^ cells was induced as described before (Hégarat et al, 2020). Briefly, cells were treated with 2 µg/ml Doxycycline (D3447 from Sigma-Aldrich) + 3 µM Asunaprevir (S4935 from Selleckchem) for 2 h. Then, 500 µM Auxin (I5148 from Sigma-Aldrich) was added to the cell culture medium for at least 4 h.

Mitotic slippage was induced by using the combination of Monastrol + MPI-0479605, except for the following figures in which mitotic slippage was induced by depleting CCNB1: Figs. EV1K, EV2M and EV3E; Appendix Fig. S1C.

To induce cytokinesis failure, cells were incubated with DMSO (276855 from Sigma-Aldrich) or with 20 µM Blebbistatin (B0560 from Sigma-Aldrich) or with 20 µM Paprotrain (HY-101298 from Medchem) for at least 4 h. Actin staining using Phaloidin (A30107 from Thermo Fisher) or β-Catenin (1/250; C2206 from Sigma-Aldrich) staining were used to ascertain the binucleation of the CF-generated tetraploid cells. After 24 h of treatment with Blebbistatin, we got a mixed population composed of 49 ± 5 diploid cells and 51 ± 5% of tetraploid cells. This efficiency was significantly higher than that with the MKLP2 inhibitor—Genistein—used in our previous work—around 20% of tetraploid cells (Gemble et al, 2022), justifying our choice to use Blebbistatin in this study.

Cytokinesis failure was induced using Blebbistatin except for the following figures in which cytokinesis failure was induced by treating cells with Paprotrain: Appendix Fig. S1B.

To induce endoreplication, cells were incubated with DMSO (276855 from Sigma-Aldrich) or with 20 µM SP600125 (HY-12041 from Medchem) for at least 4 h. After 24 h of treatment, we got a mixed population composed of 74 ± 6% of diploid cells and 26 ± 6% of tetraploid cells.

Alternatively, to induce endoreplication, CCNA2 depletion in RPE CCNA2^AID^ cells was induced as described before (Hégarat et al, 2020). Briefly, cells were treated with 2 µg/ml Doxycycline (D3447 from Sigma-Aldrich) + 3 µM Asunaprevir (S4935 from Selleckchem) for 2 h. Then, 500 µM Auxin (I5148 from Sigma-Aldrich) was added to the cell culture medium for at least 4 h.

Endoreplication was induced using SP600125 except for the following figures, in which endoreplication was induced by depleting CCNA2: Appendix Fig. S1D.

Each treatment generates a mixed population of diploid and tetraploid cells. After synchronising cells in G1 (see the 'Cell cycle synchronisation' section), diploid (2N) and tetraploid (4 N) cells are easily distinguished based on their nuclear size (tetraploid nuclei in G1 are twice bigger compared to diploid nuclei in G1), measured using Image J software V2.1.0/1.53c, https://imagej.net/software/fiji/downloads, as previously described (Gemble et al, 2022).

#### Cell cycle synchronisation

To obtain a synchronous population of G1 cells, after 4 h of WGD induction (see “Induction of WGD in human cell lines” section) 1 µM Palbociclib (Cdk4/6 inhibitor, S1579 from Selleckchem) was added to the cell culture media for 16 h (while keeping the drug to induce WGD) to accumulate/synchronise cells at the G1/S transition.

To analyze the first G2 upon WGD, Palbociclib was washed five times using PBS 1X and cells were released in the presence of 10 µM RO-3306 (HY-12529 from Medchem) to block them in G2.

To analyze asynchronous cells, WGD induction was done for 4 h before fixing the cells.

### Immunofluorescence

#### Preparation and imaging of human cells

Cells were plated on cover slips in 12-well plates and treated with the indicated drugs. To label cells, they were fixed using 4% of paraformaldehyde (15710 from Electron Microscopy Sciences) + Triton X-100 (2000-C from Euromedex) 0.1% in PBS (20 min at 4 °C) or for alternative H3K9me2 staining with 100% cold Methanol (32213 from Honeywell) (10 min at −20 °C). Then, cells were washed three times using PBS-T (PBS 1X + 0.1% Triton X-100 + 0.02% Sodium Azide) and incubated with PBS-T + BSA (04-100-812-C from Euromedex) 1% for 30 min at RT. After three washes with PBS-T + BSA, primary and secondary antibodies were incubated in PBS-T + BSA 1% for 1 h and 30 min at RT, respectively. After two washes with PBS, cells were incubated with 3 μg/ml DAPI (4’,6-diamidino-2-phenylindole; D8417 from Sigma-Aldrich) for 15 min at RT. After two washes with PBS, slides were mounted using 1.25% n-propyl gallate (Sigma, P3130), 75% glycerol (bidistilled, 99.5%, VWR, 24388-295), 23.75% H_2_O.

Images were acquired on an upright widefield microscope (DM6B, Leica Systems, Germany) equipped with a motorised XY and a 40X objective (HCX PL APO 40X/1,40-0,70 Oil from Leica). Acquisitions were performed using Metamorph 7.10.1 software (Molecular Devices, USA) and a sCMOS camera (Flash 4V2, Hamamatsu, Japan). Stacks of conventional fluorescence images were collected automatically at a Z-distance of 0.5 µm (Metamorph 7.10.1 software; Molecular Devices, RRID:SCR_002368).

#### Preparation and imaging of mouse megakaryocytes and mouse hepatocytes

For megakaryocytes, cells were plated on cover slips in 24-well plates and fixed using 4% of paraformaldehyde (15710 from Electron Microscopy Sciences) + Triton X-100 (2000-C from Euromedex) 0.1% in PBS (20 min at RT) or for microtubule staining with 100% cold MetOH (32213 from Honeywell) (10 min −20 °C). For hepatocytes, cells were plated on cover slips in six-well plates and fixed using 4% of paraformaldehyde (15710 from Electron Microscopy Sciences) or for microtubule staining with 100% MetOH (32213 from Honeywell) (10 min −20 °C).

Then, cells were washed three times using PBS-T (PBS 1X + 0.1% Triton X-100 + 0.02% sodium azide) and incubated with PBS-T + 1% BSA (04-100-812-C from Euromedex) 1% for 30 min at RT. After three washes with PBS-T + BSA, primary and secondary antibodies were incubated in PBS-T + BSA 1% for 3 and 2 h at RT, respectively. After two washes with PBS, cells were incubated with 3 μg/ml DAPI (4’,6-diamidino-2-phenylindole; D8417 from Sigma-Aldrich) for 15 min at RT. After two washes with PBS, slides were mounted using 1.25% n-propyl gallate (Sigma, P3130), 75% glycerol (bidistilled, 99.5%, VWR, 24388-295), 23.75% H_2_O.

Images were acquired on a spinning disk microscope (Gataca Systems, France). Based on a CSU-W1 (Yokogawa, Japan), the spinning head was mounted on an inverted Eclipse Ti2 microscope equipped with a motorised XY Stage (Nikon, Japan). Images were acquired through a 100×1.4NA Plan-Apo objective with a sCMOS camera (Prime95B, Photometrics, USA). Optical sectioning was achieved using a piezo stage (Nano-z series, Mad City Lab, USA). Gataca Systems’ laser bench was equipped with 405, 491 and 561 nm laser diodes, delivering 150 mW each, coupled to the spinning disk head through a single-mode fibre. Multi-dimensional acquisitions were performed using Metamorph 7.10.1 software (Molecular Devices, USA).

#### Preparation and imaging of *Drosophila* salivary glands

*Drosophila* salivary glands from third instar larvae were dissected in PBS and fixed for 30 min in 4% paraformaldehyde in PBS. They were washed three times in PBS-T 0.3% (PBS, 0.3% Triton X-100, T9284 from Sigma), 15 min for each wash, and incubated for 1 h with blocking solution (PBS, 0.3% Triton X-100 + 5% normal goat serum, 10098792 from Gibco). Then, the salivary glands were washed three times in PBS, 15 min for each wash and incubated overnight at 4 °C with primary antibodies at the appropriate dilution in the blocking solution. Tissues were washed three times in PBS-T 0.3%, 15 min for each wash, and incubated overnight at 4 °C with secondary antibodies diluted in the blocking solution. Salivary glands were then washed three times in PBS-T 0.3%, 15 min for each wash, rinsed in PBS and incubated with 3 μg ml^−1^ DAPI (4′,6-diamidino-2-phenylindole; Sigma-Aldrich D8417) at room temperature for 30 min. Salivary glands were then washed in PBS at room temperature for 30 min and mounted in clearing solution (Rapiclear 1.49 Sunjin Lab, RC149002).

Images were acquired as previously described in the section 'Preparation and imaging of mouse megakaryocytes and mouse hepatocytes'.

#### Structural illumination microscopy

Images were acquired as previously described in the section 'Preparation and imaging of mouse megakaryocytes and mouse hepatocytes'.

Super resolution was achieved on the CSU-W1 spinning disk equipped with a super-resolution module (Live-SR, Gataca systems). This module is based on structured illumination with the optical reassignment technique and online processing, leading to a two-time resolution improvement. The method called multifocal SIM (MSIM) allows combining the doubling resolution together with the physical optical sectioning of confocal microscopy.

#### Primary and secondary antibodies

Primary and secondary antibodies were used at the following concentrations: β-catenin (1/250; C2206 from Sigma-Aldrich, RRID:AB_476831), Lamin A/C (1/500; SAB4200236 from Sigma-Aldrich, RRID:AB_10743057), Lamin B1 (1/500; ab16048 from Abcam, RRID:AB_443298), H3S10 (1/500, 9706 from Cell Signaling, RRID:AB_331748), Lamin A/C (1:50, ADL101 from DHSB, RRID:AB_528332), Lamin B (1/50, ADL67.10 from DHSB, RRID:AB_528336), H3K9ac (1/500, 9649S from Ozyme; RRID:AB_823528), H3K14ac (1/500, ab52946 from Abcam, RRID:AB_880442); H3K27ac (1/500, 39685 from Active Motif, RRID:AB_2793305), DM1A (1/1000, T9026 from Sigma-Aldrich, RRID:AB_477593), H3K9me2 (1/500, 39239 from Active Motif, RRID:AB_2793199), H3K9me2 (1/500, ab1220 from Abcam; RRID:AB_449854), H3K9me3 (1/500, ab8898 from Abcam, RRID:AB_306848), H3K27me3 (1/500, 39155 from Active motif, RRID:AB_2561020), HP1α (1/500, ab109028 from Abcam, RRID:AB_10858495), Lap2β (1/250, 611000 from BD Bioscience, RRID:AB_398313), LBR (1/500, ab232731 from Abcam, RRID:AB_3246426), Nuclear Pore Complex (1/250, ab24609 from Abcam, RRID:AB_448181), Emerin (1/150, 10351-1-AP from ProteinTech, RRID:AB_2100056), goat anti-Rabbit IgG (H + L) Highly Cross-Adsorbed Secondary Antibody, Alexa Fluor 647 (1/250; A21245 from ThermoFisher, RRID:AB_2535813), Goat anti-Rabbit IgG (H + L) Highly Cross-Adsorbed Secondary Antibody, Alexa Fluor 546 (1/250; A-11035 from Thermo Fisher, RRID:AB_2534093), Goat anti-Mouse IgG1 Cross-Adsorbed Secondary Antibody, Alexa Fluor™ 488 (1/250, A-21121 from Thermo Fisher, RRID:AB_2535764), Goat anti-Mouse IgG2b Cross-Adsorbed Secondary Antibody, Alexa Fluor™ 488 (1/250, A-21141 from Thermo Fisher, RRID:AB_2535778), Goat anti-Mouse IgG2a Cross-Adsorbed Secondary Antibody, Alexa Fluor™ 546 (1/250, A-21133 from Thermo Fisher, RRID:AB_2535772), Alexa Fluor™ Plus 647 Phalloidin (1/2000, A30107 from ThermoFisher).

### 3D cultures

#### Mitotic slippage on 3D cultures

To generate spheroids, 500 cells per well were seeded into 96 ultra-low attachment well plates (7007 from Corning) in the presence of DMSO (276855 from Sigma- Aldrich) or with 50 µM monastrol (S8439 from Selleckchem) and 1 µM MPI-0479605 (S7488 from Selleckchem). Plates were spun down at 200×*g* for 3 min, to allow spheroid formation, and incubated for 24 h at 37 °C.

#### Immunostaining

Spheroids were collected and washed quickly with PBS prior fixation using 4% of paraformaldehyde (15710 from Electron Microscopy Sciences) in PBS for 40 min. Then, spheroids were permeabilised for 5 min using Triton X-100 (2000-C from Euromedex) 0.3% in PBS and blocked for 30 min using Blocking Buffer (BB = PBS 1X + 0.3% Triton X-100 + 0.02% sodium azide + 3% BSA). Aggregates were incubated with primary antibodies diluted into BB O/N. After three washes using BB, spheroids were incubated with secondary antibodies in BB for 3 h. Cells were then washed several times for 2 h in BB and mounted on glass with EverBrite (Biotium). For primary and secondary antibodies see 'Immunofluorescence microscopy and antibodies' section.

### Time-lapse microscopy

Cells were plated on a four-well dish (627870 from Greiner). Methylstat (2 µM) or Nocodazole (2 µM) were added 2 h before to start the movies. To follow microtubules or actin, SPY650-Tubulin (SC503 from Tebubio) or SPY650-FastAct (SC505 from Tebubio), respectively, were added one hour before to start the movie.

Images were acquired on a spinning disk microscope (Gataca Systems, France). Based on a CSU-W1 (Yokogawa, Japan), the spinning head was mounted on an inverted Eclipse Ti2 microscope equipped with a motorised XY Stage (Nikon, Japan). Images were acquired through a 40X NA 1.3 oil objective with a sCMOS camera (Prime95B, Photometrics, USA). Optical sectioning was achieved using a piezo stage (Nano-z series, Mad City Lab, USA). Gataca Systems’ laser bench was equipped with 405, 491 and 561 nm laser diodes, delivering 150 mW each, coupled to the spinning disk head through a single-mode fibre. Laser power was chosen to obtain the best ratio of signal/background while avoiding phototoxicity. Multi-dimensional acquisitions were performed using Metamorph 7.10.1 software (Molecular Devices, USA). Stacks of conventional fluorescence images were collected automatically at a Z-distance of 0.5 µm (Metamorph 7.10.1 software; Molecular Devices, RRID:SCR_002368). Images are presented as maximum intensity projections generated with ImageJ software (RRID:SCR_002285), from stacks deconvolved with an extension of Metamorph 7.10.1 software.

### FACS of diploid and tetraploid cells

A mix of diploid and tetraploid cells synchronised in G1 or in G2/M (see 'Cell cycle synchronisation' section) were incubated with 2 µg ml^−1^ Hoechst 33342 (Sigma-Aldrich 94403) for 1 h at 37 °C, 5% CO_2_. Then, a single cell suspension was generated. Cells were washed using PBS, the supernatant was removed, and cells were resuspended in cell culture medium containing 1 µM EDTA (10135423 from Invitrogen). Fluorescence-activated cell sorting (FACS) was performed using Sony SH800 FACS (BD FACSDiva Software Version 8.0.1), as previously described (Gemble et al, 2022). Compensations were performed using the appropriate negative control samples.

### Hi-C experiment

Diploid and tetraploid cells were isolated using flow cytometry (see “FACS of diploid and tetraploid cells” section). Library preparation for Hi-C was conducted as previously described (Lambuta et al, 2023). Briefly, the cells were fixed with formaldehyde, and the fixed nuclei were extracted for further processing. Chromatin was digested with MboI restriction enzyme (New England Biolabs, R0147), and the digested ends were biotinylated. Next, a proximity ligation was carried out, followed by chromatin crosslink reversal. DNA was purified, sheared via sonication, and size-selected for library preparation. Biotin-marked DNA fragments were pulled down with Dynabeads MyOne Streptavidin C1 (Thermo Fisher Scientific, 65001).

Hi-C library preparation continued with end polishing, A-tailing, and Illumina TruSeq unique dual-index ligation reactions. Lastly, libraries were amplified via PCR, and sequenced on an Illumina HiSeq X platform, PE150 configuration.

### Atomic force microscopy

Atomic force microscopy measurements were performed on glass-bottom dishes (ibidi) with a JPK NanoWizard 4 (Bruker Nano) microscope mounted on an Eclipse Ti2 inverted fluorescent microscope (Nikon) and operated via JPK SPM Control Software v.6. MLCT triangular silicon nitride cantilevers (Bruker) were used to access the mechanical properties of nuclei. Forces of up to 2 nN were applied at 10 micron per second constant cantilever velocity, also ensuring an indentation depth of at ~500 nm.

### ATACseq

#### Sample preparation

The samples were prepared using the following kit 53150 from Active Motif and according to the manufacturer’s protocol. We isolated diploid and tetraploid G1 cells using flow cytometry (see 'FACS of diploid and tetraploid cells' section) and used 1 × 10^5^ cells per condition. Spike in (53154 from Active Motif) was added according to the manufacturer’s protocol.

#### Sequencing

The pool was quantified by qPCR using the KAPA library quantification kit (Roche). Sequencing was carried out on the NovaSeq X Plus instrument from Illumina based on a 2 × 100 cycle mode (paired-end reads, 100 bases) targeting around 50 million clusters per sample.

### Quantification and statistical analysis

#### Immunofluorescence

Image analysis and quantifications were performed using Image J software V2.1.0/1.53c, https://imagej.net/software/fiji/downloads (Schindelin et al, 2012). Images are presented as maximum intensity projections generated with ImageJ software (RRID:SCR_002285).

For the 3D reconstruction, images were converted in Imaris files using ImarisFileConverter 9.9.1 (Bitplane, RRID:SCR_007370). Then, images were imported into Imaris software v.9.6.0 (Bitplane, RRID:SCR_007370). Nuclear shapes were reconstructed based on the DNA staining. 3D views were generated using Imaris software and saved as TIFF.

To quantitatively measure nuclear shapes, the 'wand' tool from Image J V2.1.0/1.53c software was used to select the nuclei based on the DAPI staining. Based on this segmentation, the circularity and solidity indexes were then measured using the ''shape descriptors' tool from Image J V2.1.0/1.53c software.

To quantify fluorescence intensity, images were converted into maximum intensity projections. The fluorescence intensity across the entire nucleus (e.g., histone acetylation levels) was measured using the 'Polygon Sections' tool in ImageJ (v2.1.0/1.53c). Then the mean intensity (i.e. the fluorescence intensity divided by the nuclear area) was measured to normalise for nuclear size, thereby excluding potential effects due to the larger size of tetraploid nuclei. For binucleated cells generated by CF, the overall mean intensity of both nuclei was measured.

The mean fluorescence intensity at the nuclear envelope or locally at the intact/deformed regions (e.g. LBR or H3K9me2 levels) were measured using the 'segmented lines' tool from ImageJ V2.1.0/1.53c software. When it was possible, Lamin A/C or Lamin B1 staining were used to visualise and segment the nuclear envelope. If not, the nuclear envelope measurement was performed through segmentation of the nuclear periphery in the DAPI channel, since in these cells, the DNA is closely associated with the Lamina network. Since diploid cells were not deformed, the mean fluorescence intensity was compared at two random regions (i.e. region 1 and region 2) to visualise the distribution of the signal, while in newly born tetraploid cells, intact and deformed areas were compared.

The coefficient of variation (CoV) was measured by dividing the standard deviation of the signal by the mean fluorescence intensity for each cell.

To define small and large megakaryocytes or hepatocytes, the median nuclear size was used as a threshold.

For the figures, images were processed on ImageJ V2.1.0/1.53c software, and mounted using Affinity Designer 2, https://affinity.serif.com/fr/designer/.

#### Time-lapse microscopy

Image analysis and quantifications were performed using Image J software V2.1.0/1.53c, https://imagej.net/software/fiji/downloads. Videos were corrected using '3D correct drift' plugin with Image J V2.1.0/1.53c software to keep the cell of interest at the centre of the region of interest.

For Fig. 4A, nuclei masks were generated using the Fiji Plugin as described in (Almonacid et al, 2019). Briefly, this process involves denoising (using the Pure Denoise Fiji Plugin (Luisier et al, 2011)) and thresholding the image over time, to get one ROI (the nucleus) on each frame. A custom macro was then used to project the contours over time, with each contour coloured according to its corresponding frame. The Analyze Particles function was applied to the masks generated by the Plugin, and the contours’ ROIs were colour-coded according to their frame using the BAR Plugin.

To analyze nuclear shapes by live imaging, single cells were followed, and nuclear circularity and solidity indexes were measured in G2 before the NEBD and in G1 after complete DNA decondensation. The results were plotted using the 'before-after' representation from GraphPad Prism (RRID:SCR_002798) version 10 for Mac, GraphPad Software, La Jolla, California, USA, www.graphpad.com. The delta circularity was measured by subtracting the nuclear circularity index in G2 by the one in G1 for each single cell.

For the figures, images were processed on ImageJ V2.1.0/1.53c software, and mounted using Affinity Designer 2, https://affinity.serif.com/fr/designer/.

#### ATACseq

Quality control was performed using FastQC and MultiQC. Sequencing reads were aligned to the human reference genome (hg38) using the BWA-MEM2 algorithm. Duplicate reads were marked using the Picard MarkDuplicates tool. Only reads with a mapping quality score ≥40 were retained, using Samtools. Spike-in normalisation was conducted following the guidelines provided by ActiveMotif. Briefly, the number of reads uniquely mapped to the Drosophila (dm6) genome was quantified per sample. A normalisation ratio was then calculated based on the lowest number of Drosophila-mapped reads among all samples. This ratio was subsequently applied to downsample the uniquely mapped reads to the human genome in each sample with Samtools. Samtools was also used to shift BAM files. Peak calling was performed with MACS3 callpeak. Peaks overlapping ENCODE blacklist regions were removed using subtractBed. Peaks with log2 fold change (log2FC) <3 and −log10(FDR) <2 were filtered out. Differential peak analysis was carried out using DiffBind in a replicate mode. Finally, peak annotation was performed using HOMER.

#### Atomic force microscopy

Analyses were performed with JPK Data Processing Software v.6 (Bruker Nano) by (1) removing the offset from the baseline of raw force curves, (2) identifying the contact point and subtracting cantilever bending then and (3) fitting the Hertz model with correct tip geometry to quantify the reported Young’s Modulus of nuclei.

#### Hi-C data

Generation of Hi-C contact maps and further analyses, including inter-chromosomal interactions, intercompartmental interactions and conservation of insulating boundaries, were performed as previously described (Lambuta et al, 2023). Briefly, reads were mapped to the human hg19 reference genome using bwa mem and processed with the Juicer pipeline (Rao et al, 2014). Hi-C maps of the same condition were then merged and normalised using the Knight–Ruiz method (Knight and Ruiz, 2013).

To study inter-chromosomal interaction changes, Hi-C interactions were aggregated between each pair of chromosomes. The matrix was balanced using iterative correction, obtaining IICE. Chromosomes from 1 to 14 and X were categorised as long, whereas chromosomes from 15 to 22 were categorised as short. To compare control and WGD inter-chromosomal contact change, the Log2 fold change of IICE(WGD) and IICE(Control) was done. Chromosome interactions were then split into long–long, long–short and short–short. These categories were compared by computing a Mann–Whitney test *P* value between *R* values of each pair of categories.

Compartments were called using Calder (Liu et al, 2021) at 50 kb resolution. The intra-chromosomal distance-normalised contacts of each genomic bin were extracted by considering the 1-Mb window around the bin to smooth the signal and ensure enough coverage at long distances. Then the proportion of interactions of each bin to bins of the eight compartment levels was computed. This value was then normalised by the percentage of bins belonging to each compartment level. Finally, this number was normalised by its sum for each bin. This value represents the segregation level of a bin to the compartment it belongs to. Given WGD and control conditions, we computed the ratio between the number of bins in comp1, which lose compartment segregation with comp2, and the number of bins in comp1, which gain compartment segregation with comp2. We then computed the -log2 average of these two quantities, representing the average segregation changes between compartment levels comp1 and comp2. To assess the loss of segregation for a specific compartment level comp, the ratio between the number of bins in comp losing segregation and the number of bins in comp gaining segregation was calculated. A *p* value was finally calculated with a Wilcoxon rank-sum test between A vs A, B vs B and A vs B values.

TAD boundaries were calculated by identifying local minima of insulation scores using the FANC package, using a 400 kb window around each bin. We then considered the top 300 insulating boundaries in control and WGD samples and took the shared ones between the two conditions. A *p* value was calculated between the two conditions with a Wilcoxon signed-rank test.

#### Statistical analysis

The statistical significance of differences was calculated using GraphPad Prism (RRID:SCR_002798) version 10 for Mac, GraphPad Software, La Jolla California USA, www.graphpad.com. The statistical test used for each experiment is indicated in the figure legends. Each representative image originates from the analyzed dataset and are representative of the associated quantifications. ns not significant.

## Supplementary information

Appendix

Peer Review File

Movie EV1

Movie EV2

Movie EV3

Movie EV4

Movie EV5

Movie EV6

Movie EV7

Movie EV8

Movie EV9

Movie EV10

Source data Fig. 1

Source data Fig. 2

Source data Fig. 3

Source data Fig. 4

Source data Fig. 5

Source data Fig. 6

Source data Fig. 7

Expanded View Figures

## Data Availability

The datasets generated during and/or analysed during the current study are available here: https://figshare.com/s/5e2a55869830ad68aebe. The ATACseq data from this publication have been deposited in the GEO database https://www.ncbi.nlm.nih.gov/geo/query/acc.cgi?acc=GSE344975 and assigned the identifier GSE344975. The HiC data from this publication have been deposited in the GEO database https://www.ncbi.nlm.nih.gov/geo/query/acc.cgi?acc=GSE345433 and assigned the identifier GSE345433. The source data of this paper are collected in the following database record: biostudies:S-SCDT-10_1038-S44318-026-00912-y.
