# Supplementary material for: Whole genome duplication through mitotic slippage causes nuclear instability
Source: EMBO J. 2026 Sep 19;45(20):7103–35. doi: 10.1038/s44318-026-00912-y (PMC13672370; doi:10.1038/s44318-026-00912-y)
Supplement: Supplementary file 1 [file 44318_2026_912_MOESM1_ESM.pdf]

APPENDIX FOR

Whole genome duplication through mitotic slippage causes nuclear instability

APPENDIX FIGURES

Page 2: Appendix Figure S1: Mitotic slippage unlike cytokinesis failure or endoreplication, generates polyploid cells with abnormal nuclear architecture

Page 4: Appendix Figure S2: The microtubule cytoskeleton is the main contributor to nuclear deformations in tetraploid cells generated by mitotic slippage

Page 6: Appendix Figure S2: The microtubule cytoskeleton is the main contributor to nuclear deformations in tetraploid cells generated by mitotic slippage

APPENDIX FIGURES

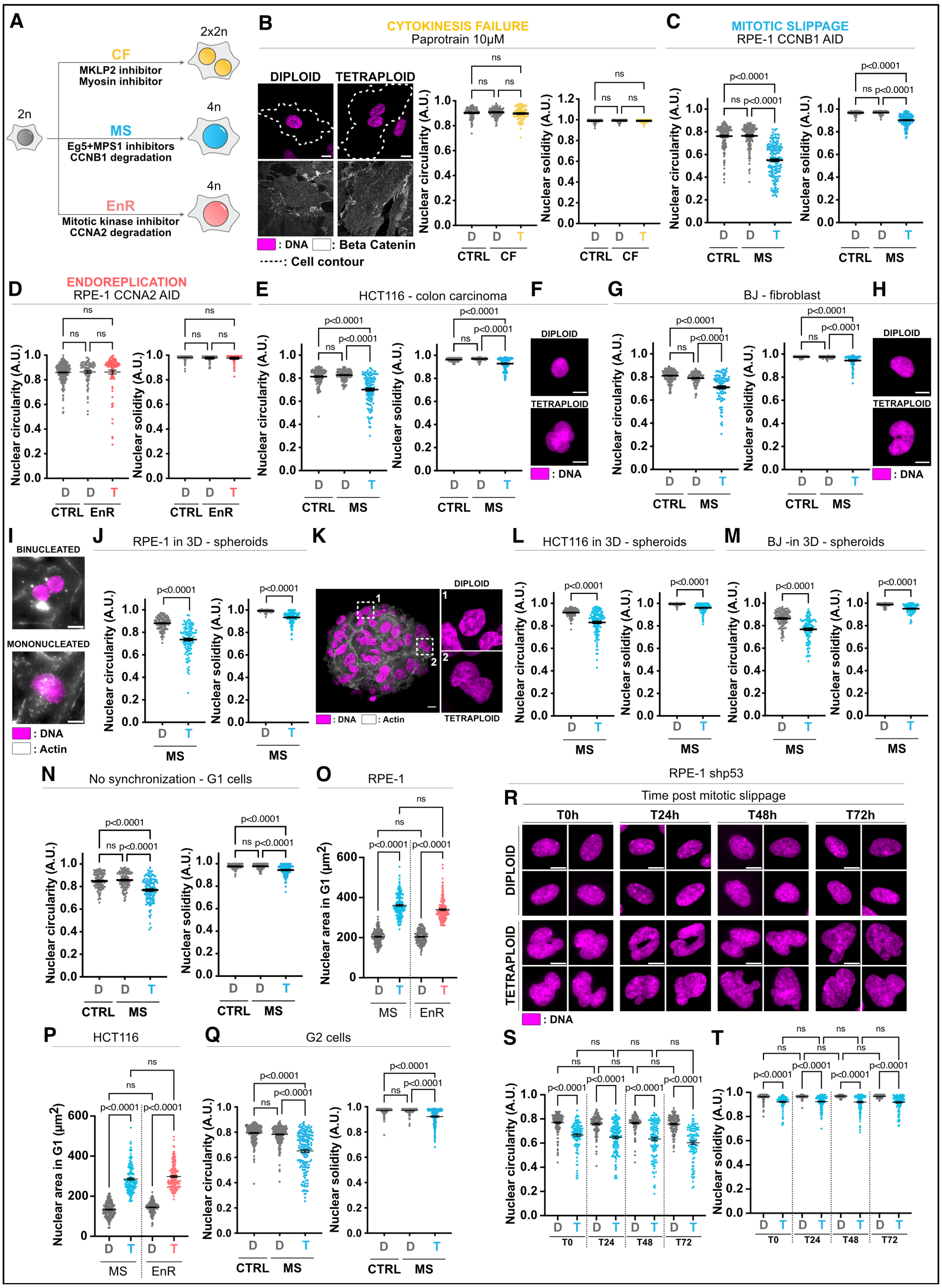

Appendix Figure S1: Mitotic slippage unlike cytokinesis failure or endoreplication, generates polyploid cells with abnormal nuclear architecture
(A) Schematic representation showing the methods used to induce whole genome duplication. **(B)** Left panel – representative images showing diploid and CF-generated

tetraploid G1 RPE-1 cells. Tetraploid cells were generated by using 10μM Paprotrain. β-Catenin in grey, DNA in magenta. Right panel – Graph showing nuclear circularity and solidity in diploid and in CF-generated tetraploid G1 RPE-1 cells (in grey and yellow, respectively). Mean ± SEM, >100 G1 cells analysed per condition, three independent experiments. **(C-D)** Graphs showing nuclear circularity and solidity in diploid and in **(C)** MS- and **(D)** EnR-generated tetraploid G1 RPE-1 cells (in blue and red, respectively). Tetraploid cells were generated by depleting CCNB1 (MS) or CCNA2 (EnR). Mean ± SEM, >150 G1 cells analysed per condition, three independent experiments. **(E)** Graphs presenting nuclear circularity and solidity in diploid and in MS-generated tetraploid HCT116 cells in G1 (in grey and blue, respectively). Mean ± SEM, >100 G1 cells analysed per condition, three independent experiments. **(F)** Representative images showing diploid and MS-generated tetraploid HCT116 nuclei. DNA in magenta. **(G)** Graphs showing nuclear circularity and solidity in diploid and in MS-generated tetraploid BJ cells in G1 (in grey and blue, respectively). Mean ± SEM, >100 G1 cells analysed per condition, three independent experiments. **(H)** Representative images presenting diploid and MS-generated tetraploid BJ nuclei. DNA in magenta. **(I)** Representative images showing mononucleated and binucleated tetraploids G1 cells generated by CF. DNA in magenta. Actin in grey. **(J)** Graph showing nuclear circularity and solidity in diploid and in MS-generated tetraploid RPE-1 cells cultured in 3D (in grey and blue, respectively). Mean ± SEM, >100 G1 cells analysed per condition, three independent experiments. **(K)** Representative images showing an RPE-1 spheroid containing diploid and MS-generated tetraploid cells. Actin in grey, DNA in magenta. **(L-M)** Graphs showing nuclear circularity and solidity in diploid and in MS-generated tetraploid **(L)** HCT116 and **(M)** BJ cells cultured in 3D (in grey and blue, respectively). Mean ± SEM, >110 G1 cells analysed per condition, three independent experiments. **(N)** Graph showing nuclear circularity and solidity in diploid and in MS-generated tetraploid asynchronous RPE-1 cells (in grey and blue, respectively). Mean ± SEM, >110 interphase cells analysed per condition, three independent experiments. **(O-P)** Graphs presenting nuclear area in diploid and in MS- and EnR-generated tetraploid **(O)** G1 RPE-1 and **(P)** HCT116 cells (in grey, blue and red, respectively). Mean ± SEM, >150 G1 cells analysed per condition, three independent experiments. **(Q)** Graph showing nuclear circularity and solidity in diploid and in MS-generated tetraploid G2 RPE-1 cells (in grey and blue, respectively). Mean ± SEM, >170 G2 cells analysed per condition, three independent experiments. **(R)** Representative images showing diploid and MS-generated tetraploid RPE-1 shP53 cells. DNA in magenta. **(S-T)** Graphs showing nuclear **(S)** circularity and **(T)** solidity in diploid and in MS-generated tetraploid RPE-1 shp53 cells (in grey and blue, respectively). Mean ± SEM, >100 interphase cells analysed per condition, three independent experiments. Scale bars: 10μm. D=diploid. T=tetraploid. MS=mitotic slippage. CF=cytokinesis failure. EnR= endoreplication. A.U.=arbitrary unit. **(B,C,D,E,G,N,O,P,R,S,T)** ANOVA-test (one sided). **(J,L,M)** t-test (two-sided). ns=not significant.

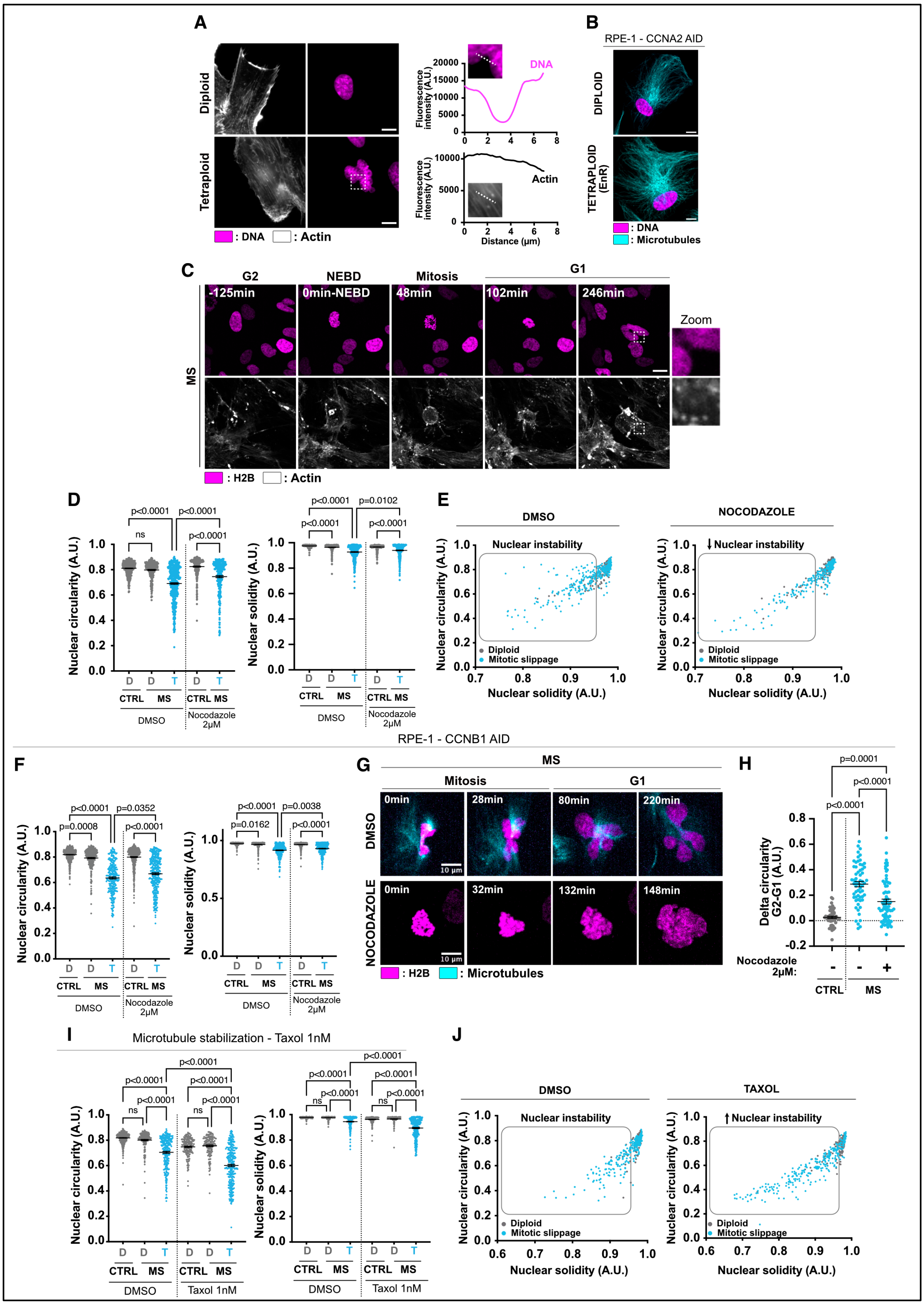

Appendix Figure S2: The microtubule cytoskeleton is the main contributor to nuclear deformations in tetraploid cells generated by mitotic slippage

(A) Left panel - Representative images showing diploid and MS-generated RPE-1 cells. Actin in grey, DNA in magenta. Right panel - Graphs showing line scan

measurements from the left panel. **(B)** Representative images showing microtubules (in cyan) in diploid and in EnR-generated RPE-1 cells. DNA in magenta. **(C)** Stills from time-lapse imaging of RPE-1 cells stably expressing mCherry-H2B (in magenta) performing MS. Actin was labelled using SPY650-FastAct (in grey). **(D)** Graphs showing nuclear circularity (left panel) and solidity (right panel) in diploid and in MS-generated tetraploid RPE-1 cells (in grey and blue, respectively) treated with DMSO or with 2μM Nocodazole. Mean ± SEM, >105 G1 cells analysed per condition, three independent experiments. **(E)** Graphs presenting the nuclear circularity and solidity from **(D)** in diploid and tetraploid G1 RPE-1 cells treated with DMSO or with 2μM Nocodazole. **(F)** Graphs showing nuclear circularity and solidity in diploid and in MS-generated tetraploid RPE-1 CCNB1 cells (in grey and blue, respectively) treated with DMSO or with 2μM Nocodazole. Tetraploid cells were generated by depleting CCNB1. Mean ± SEM, >80 G1 cells analysed per condition, three independent experiments. **(G)** Stills from time-lapse imaging of RPE-1 CCNB1 cells stably expressing mCherry-H2B (in magenta) treated with DMSO or 2μM Nocodazole and performing MS. **(H)** Graph showing the delta circularity (defined as the difference between nuclear circularity in G2 vs in G1) upon mitotic division (in grey) or MS (in blue) treated with DMSO (-) or 2μM Nocodazole. Tetraploid cells were generated by depleting CCNB1. Mean ± SEM, >50 cells analysed per condition, three independent experiments. **(I)** Graphs displaying nuclear circularity and solidity indices in diploid and in MS-generated tetraploid RPE-1 cells (in grey and blue, respectively) treated with DMSO or 1nM Taxol. Mean ± SEM, >140 G1 cells analysed per condition, three independent experiments. **(J)** Graph presenting the nuclear circularity and solidity from **(I)** in diploid and tetraploid G1 RPE-1 cells treated with DMSO or with 1nM Taxol. Scale bars: 10μm. D=diploid. T=tetraploid. MS=mitotic slippage. CF=cytokinesis failure. EnR= endoreplication. A.U.=arbitrary unit. **(D,F,H,I)** ANOVA-test (one-sided). ns=not significant.

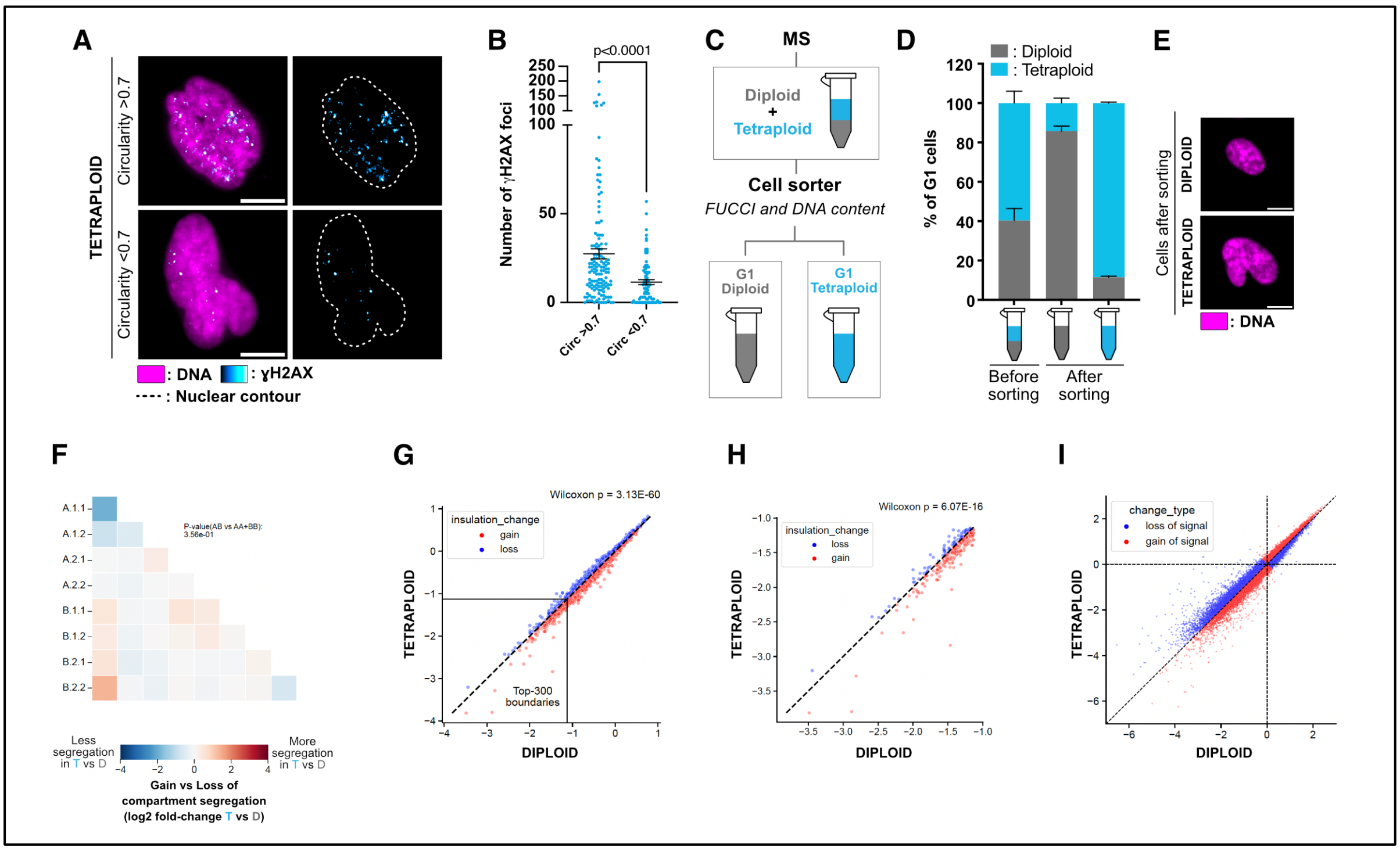

Appendix Figure S3: Comparison of chromosome interactions between tetraploid and diploid RPE1 cells

(A) Representative images of RPE-1 tetraploid cells with low nuclear deformations (circularity index >0.7) or highly deformed (circularity index <0.7). DNA in magenta. ɣH2AX in cyan. The nuclear contour is indicated. **(B)** Graph showing the number of ɣH2AX foci per tetraploid cells. Mean ± SEM, >100 interphase cells analysed per condition, three independent experiments. **(C)** Scheme presenting the experimental workflow used to isolate diploid and tetraploid cells. **(D)** Graph showing the percentage of G1 diploid (in grey) and MS-generated tetraploid (in blue) RPE-1 cells before and after cell sorting. Mean ± SEM, >900 cells analysed, five independent experiments. **(E)** Representative images showing diploid and MS-generated tetraploid cells after cell sorting. DNA in magenta. **(F-G)** Boundary insulation scores for **(F)** all and **(G)** top 300 most strongly insulating boundaries between diploid (x-axis) and tetraploid (y-axis) conditions. **(H)** Relationship between observed and expected inter-chromosomal values in tetraploid versus diploid. Each dot represents an inter-chromosomal interaction, red signifies higher and blue lower interaction signal in tetraploid compared to control. **(I)** Remodeling of interactions between chromatin sub-compartments in tetraploid and diploid cells. Blue suggests a loss of interaction, red a gain of interactions. D=diploid. T=tetraploid. MS=mitotic slippage. ns=not significant. **(B)** t-test (two-sided). **(F,G,H,I)** two-tailed Wilcoxon test.
