# Supplementary material for: Whole genome duplication through mitotic slippage causes nuclear instability
Source: EMBO J. 2026 Sep 19;45(20):7103–35. doi: 10.1038/s44318-026-00912-y (PMC13672370; doi:10.1038/s44318-026-00912-y)
Supplement: Supplementary file 2 [file 44318_2026_912_MOESM20_ESM.pdf]

Expanded View Figures

Figure EV1. Mitotic slippage, but not cytokinesis failure and endoreplication, alters histone modification.

(**A**) Schematic representation of the mean fluorescence measured at the level of the whole nucleus in diploid and tetraploid cells. (**B**) SIM representative images showing diploid and tetraploid G1 RPE-1 cells generated by EnR (left panel), by CF (central panel) or by MS (right panel). H3K9ac, H3K27ac and H3K14ac in cyan hot. (**C**) Graphs showing the relative H3K9ac (left panel) or H3K27ac (right panel) mean intensity in the nucleus in diploid (in grey) and in tetraploid (in blue) RPE-1 G1 cells stably expressing H3 WT or H3S10A. Mean ± SEM, >100 G1 cells analysed per condition, three independent experiments. (**D**) Left panel—SIM representative images showing diploid and tetraploid G1 RPE-1 cells generated by MS. Lamin A/C in yellow hot, H3K9me2 in cyan hot and DNA in magenta. Right panel—Graph presenting the relative H3K9me2 mean intensity in the nucleus in diploid (in grey) and in tetraploid (in blue) G1 RPE-1 cells. Mean ± SEM, >100 G1 cells analysed per condition, three independent experiments. (**E**) Left panel—SIM representative images showing diploid and tetraploid G1 RPE-1 cells generated by MS. Lamin A/C in yellow hot, H3K9me3 in cyan hot and DNA in magenta. Right panel—Graph presenting the relative H3K9me3 mean intensity in the nucleus in RPE-1 diploid (in grey) and in tetraploid (in blue) G1 cells. Mean ± SEM, >100 G1 cells analysed per condition, three independent experiments. (**F**) Left panel—SIM representative images showing diploid and tetraploid G1 RPE-1 cells generated by MS. Lamin A/C in yellow hot, H3K27me3 in cyan hot and DNA in magenta. Right panel—Graph presenting the Relative H3K27me2 mean intensity in the nucleus in diploid (in grey) and in tetraploid (in blue) G1 RPE-1 cells. Mean ± SEM, >100 G1 cells analysed per condition, three independent experiments. (**G**, **H**) Graphs showing the relative H3K9me2, H3K9me3 and H3K27me3 mean intensity in the nucleus in diploid (in grey) and in tetraploid G1 RPE-1 cells generated by (**G**) EnR (in red) or by (**H**) CF (in yellow). Mean ± SEM, >100 G1 cells analysed per condition, three independent experiments. (**I**) SIM representative images showing diploid and tetraploid G1 RPE-1 cells generated by MS. HP1α in cyan hot and DNA in magenta. (**J**) Graph showing the number of HP1 foci / nuclear area in diploid (in grey) and in tetraploid (in blue) RPE-1 G1 cells. Mean ± SEM, >90 G1 cells analysed per condition, at least three independent experiments. (**K**) Graph showing nuclear stiffness in diploid and in MS-generated tetraploid G1 RPE-1 CCNB1 cells (in grey and blue, respectively). MS cells were generated by depleting CCNB1. Box and whiskers ± Min to Max, >175 G1 cells analysed per condition, three independent experiments. Scale bars: 10 µm. D diploid, T tetraploid, MS mitotic slippage, EnR endoreplication, CF cytokinesis failure, AU arbitrary unit. (**C**–**H**, **J**) *t*-test (two-sided). (**K**) Kolmogorov–Smirnov test. ns not significant.

 © The Author(s)

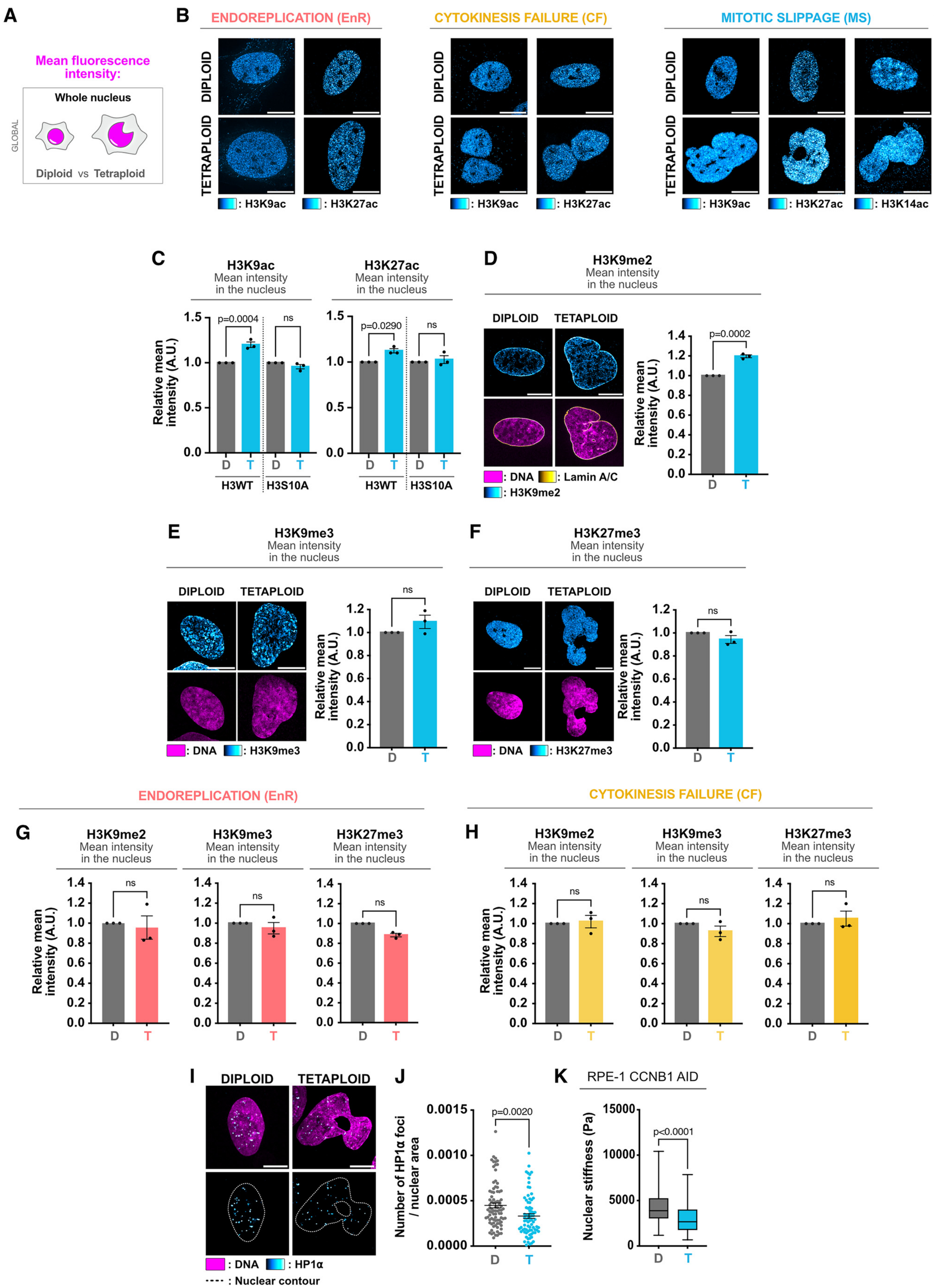
A
Mean fluorescence intensity:
Whole nucleus
GLOBAL
Diploid vs Tetraploid
B
ENDOREPLICATION (EnR)
CYTOKINESIS FAILURE (CF)
MITOTIC SLIPPAGE (MS)
DIPLOID
TETRAPLOID
: H3K9ac
: H3K27ac
: H3K14ac
C
H3K9ac
Mean intensity in the nucleus
H3K27ac
Mean intensity in the nucleus
Relative mean intensity (A.U.)
p=0.0004
p=0.0290
ns
D T D T
H3WT H3S10A
D
H3K9me2
Mean intensity in the nucleus
DIPLOID TETAPLOID
: DNA : Lamin A/C
: H3K9me2
p=0.0002
E
H3K9me3
Mean intensity in the nucleus
DIPLOID TETAPLOID
: DNA : H3K9me3
F
H3K27me3
Mean intensity in the nucleus
DIPLOID TETAPLOID
: DNA : H3K27me3
ENDOREPLICATION (EnR)
CYTOKINESIS FAILURE (CF)
G
H
H3K9me2
H3K9me3
H3K27me3
Mean intensity in the nucleus
I
DIPLOID TETRAPLOID
: DNA : HP1α
: Nuclear contour
J
Number of HP1α foci / nuclear area
p=0.0020
K
RPE-1 CCNB1 AID
Nuclear stiffness (Pa)
p<0.0001
D T

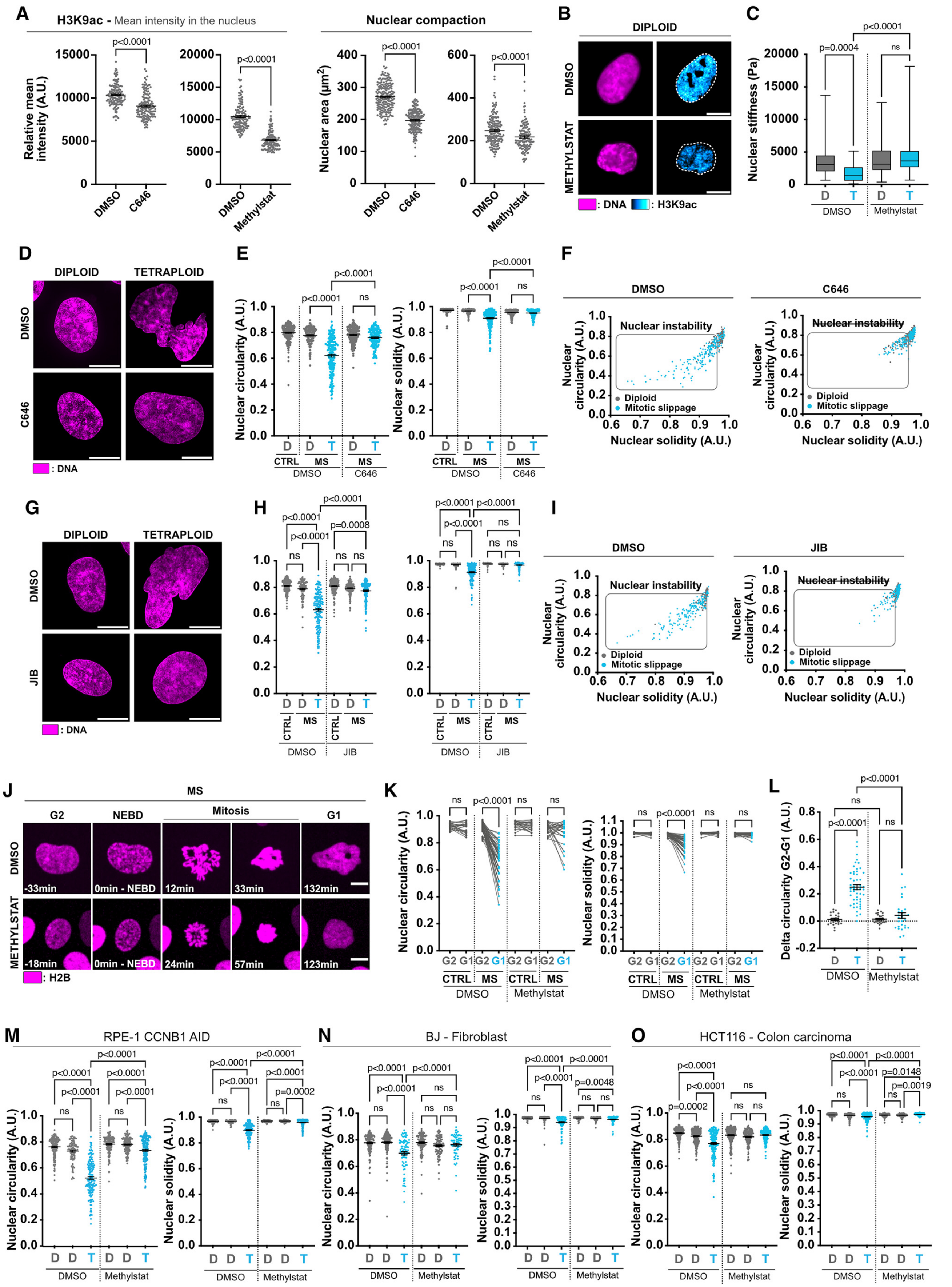

 © The Author(s)

Figure EV2. Promoting nuclear compaction is sufficient to prevent nuclear deformations upon mitotic slippage.

(**A**) Graphs presenting the H3K9ac mean intensity (left panel) or the nuclear area (right panel) in diploid RPE-1 cells. Mean ± SEM, >125 cells analysed per condition, at least three independent experiments. (**B**) Representative images showing RPE-1 diploid cells treated with DMSO or 4 µM Methylstat. H3K9ac in cyan hot, DNA in magenta. (**C**) Graph showing the nuclear stiffness in diploid (in grey) and tetraploid (in blue) RPE-1 G1 cells generated by MS treated with DMSO or 4 µM Methylstat. (**D**) Representative images showing diploid and tetraploid RPE-1 G1 cells treated with DMSO or 20 µM C646. DNA in magenta. (**E**) Graphs showing the nuclear circularity (left panel) and solidity (right panel) indices in diploid (in grey) and tetraploid (in blue) RPE-1 G1 cells generated by MS treated with DMSO or 20 µM C646. Mean ± SEM, >125 G1 cells analysed per condition, at least three independent experiments. (**F**) Graphs presenting the nuclear circularity and solidity from (**E**) in RPE-1 diploid (in grey) and tetraploid (in blue) G1 cells treated with DMSO or 20 µM C646. (**G**) Representative images showing diploid and tetraploid RPE-1 cells treated with DMSO or 5 µM JIB. DNA in magenta. (**H**) Graphs presenting the nuclear circularity (left panel) and solidity (right panel) indices in diploid (in grey) and tetraploid (in blue) RPE-1 G1 cells generated by MS treated with DMSO or 5 µM JIB. Mean ± SEM, >100 G1 cells analysed per condition, at least three independent experiments. (**I**) Graphs presenting the nuclear circularity and solidity from (**H**) in diploid (in grey) and tetraploid (in blue) RPE-1 G1 cells treated with DMSO or 5 µM JIB. (**J**) Stills from time-lapse imaging of RPE-1 cells stably expressing mCherry-H2B (in magenta) performing MS and treated with DMSO (upper panel) or 2 µM Methylstat (lower panel). (**K**) Graphs showing the nuclear circularity (left panel) and solidity (right panel) measured at the single cell levels in G2 and in G1 upon mitotic division (in grey) or MS (in blue) in RPE-1 cells treated with DMSO or 2 µM Methylstat. >30 cells analysed per condition, three independent experiments. (**L**) Graph showing the delta circularity (defined as the difference between nuclear circularity in G2 vs in G1) upon mitotic division (in grey) or MS (in blue) in RPE-1 cells treated with DMSO or 2 µM Methylstat. Mean ± SEM, >25 cells analysed per condition, three independent experiments. (**M–O**) Graphs displaying nuclear circularity and solidity in (**M**) RPE-CCNB1, (**N**) BJ or (**O**) HCT116 cells treated with DMSO or 4 µM Methylstat. (**M**) Tetraploid cells were generated by depleting CCNB1. Mean ± SEM, >65 G1 cells analysed per condition, three independent experiments. Scale bars: 10 µm. D diploid, T tetraploid, MS mitotic slippage, NEBD nuclear envelope breakdown, AU arbitrary unit. (**A**, **K**, **L**) *t*-test (two-sided). (**E**, **H**, **M–O**) ANOVA test (one-sided). (**C**) Kolmogorov–Smirnov test. ns not significant.

© The Author(s)

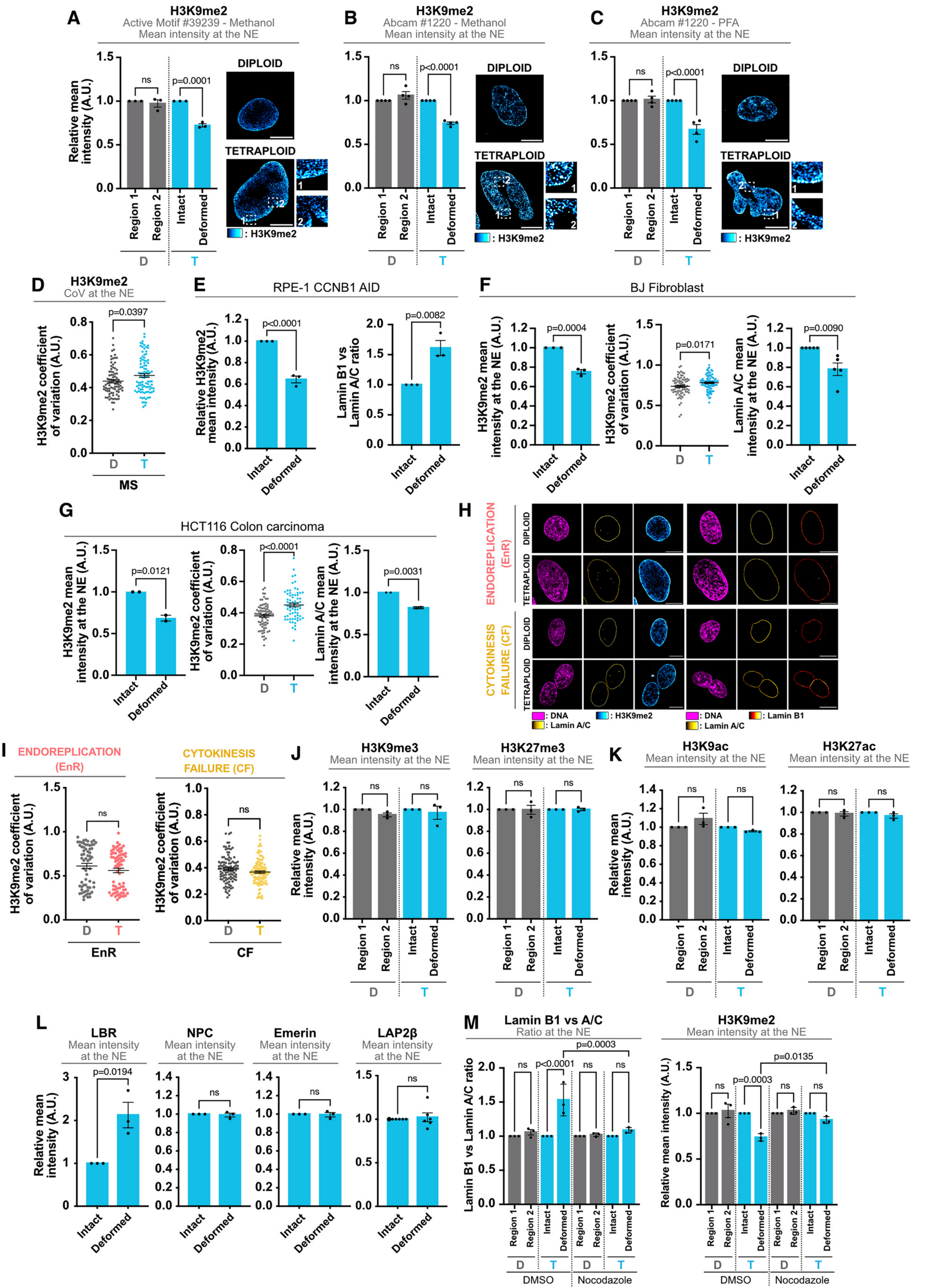
A
H3K9me2
Active Motif #39239 - Methanol
Mean intensity at the NE
B
H3K9me2
Abcam #1220 - Methanol
Mean intensity at the NE
C
H3K9me2
Abcam #1220 - PFA
Mean intensity at the NE
Relative mean intensity (A.U.)
DIPLOID
TETRAPLOID
: H3K9me2
Region 1
Region 2
Intact
Deformed
D
T
ns
p=0.0001
p<0.0001
D
H3K9me2
CoV at the NE
H3K9me2 coefficient of variation (A.U.)
p=0.0397
MS
E
RPE-1 CCNB1 AID
Relative H3K9me2 mean intensity (A.U.)
Lamin B1 vs Lamin A/C ratio
p=0.0082
F
BJ Fibroblast
H3K9me2 mean intensity at the NE (A.U.)
p=0.0004
p=0.0171
Lamin A/C mean intensity at the NE (A.U.)
p=0.0090
G
HCT116 Colon carcinoma
p=0.0121
p=0.0031
H
ENDOREPLICATION (EnR)
CYTOKINESIS FAILURE (CF)
DIPLOID
TETRAPLOID
: DNA
: Lamin A/C
: H3K9me2
: Lamin B1
I
ENDOREPLICATION (EnR)
CYTOKINESIS FAILURE (CF)
EnR
CF
J
H3K9me3
Mean intensity at the NE
H3K27me3
Mean intensity at the NE
K
H3K9ac
Mean intensity at the NE
H3K27ac
Mean intensity at the NE
L
LBR
Mean intensity at the NE
NPC
Mean intensity at the NE
Emerin
Mean intensity at the NE
LAP2β
Mean intensity at the NE
p=0.0194
M
Lamin B1 vs A/C
Ratio at the NE
Lamin B1 vs Lamin A/C ratio
p=0.0003
H3K9me2
Mean intensity at the NE
p=0.0135
DMSO
Nocodazole

 © The Author(s)

Figure EV3. Mitotic slippage promotes local changes in the Lamina network and in H3K9me2 levels.

(**A**) Left panel—Graph showing the relative H3K9me2 mean intensity at the nuclear envelope in diploid (in grey) and tetraploid (in blue) G1 RPE-1 cells. Mean ± SEM, >90 G1 cells analysed per condition, three independent experiments. Right panel—SIM representative images showing diploid and tetraploid G1 RPE-1 cells. H3K9me2 in cyan hot. (**B**) Left panel—Graph showing the relative H3K9me2 mean intensity at the nuclear envelope in diploid (in grey) and tetraploid (in blue) G1 RPE-1 cells. Mean ± SEM, >90 G1 cells analysed per condition, three independent experiments. Right panel—SIM representative images showing diploid and tetraploid G1 RPE-1 cells. H3K9me2 in cyan hot. (**C**) Left panel—Graph showing the relative H3K9me2 mean intensity at the nuclear envelope in diploid (in grey) and tetraploid (in blue) G1 RPE-1 cells. Mean ± SEM, >90 G1 cells SIM resolution representative images showing diploid and tetraploid G1 RPE-1 cells. H3K9me2 in cyan hot. (**D**) Graph presenting the H3K9me2 coefficient of variation at the nuclear envelope in MS-induced tetraploid G1 RPE-1 cells. Mean ± SEM, >90 G1 cells analysed per condition, three independent experiments. (**E**) Graphs showing the relative H3K9me2 mean intensity (left panel) or the Lamin B1 vs A/C ratio (right panel) at the nuclear envelope in MS-induced tetraploid RPE-1 CCNB1 cells. MS cells were generated by depleting CCNB1. Mean ± SEM, >90 G1 cells analysed per condition, three independent experiments. (**F**, **G**) Graphs of H3K9me2 mean intensity at the NE (left panel), H3K9me2 coefficient of variation at the NE (central panel) and Lamin A/C mean intensity at the NE (right panel) in diploid and in MS-generated tetraploid (**F**) BJ and (**G**) HCT116 cells. Mean ± SEM, >60 G1 cells analysed per condition, three independent experiments. (**H**) SIM representative images showing diploid and tetraploid cells generated by EnR or CF. H3K9me2, Lamin A/C and Lamin B1 in cyan hot, yellow hot and red hot, respectively. DNA in magenta. (**I**) Graphs showing the H3K9me2 coefficient of variation in diploid (in grey) and tetraploid G1 RPE-1 cells generated by EnR (in red) or CF (in yellow). Mean ± SEM, >90 G1 cells analysed per condition, three independent experiments. (**J**) Graphs showing the relative H3K9me3 (left panel) or H3K27me3 (right panel) mean intensity in diploid (in grey) and tetraploid (in blue) RPE-1 G1 cells. Mean ± SEM, >90 G1 cells analysed per condition, three independent experiments. (**K**) Graphs showing the relative H3K9ac (left panel) or H3K27ac (right panel) mean intensity in diploid (in grey) and tetraploid (in blue) RPE-1 G1 cells. Mean ± SEM, >90 G1 cells analysed per condition, three independent experiments. (**L**) Graphs presenting the relative mean intensity at the nuclear envelope of the indicated proteins in tetraploid RPE-1 G1 cells. Mean ± SEM, >90 G1 cells analysed per condition, at least three independent experiments. (**M**) Graphs showing the Lamin B1 vs A/C ratio (left panel) or the H3K9me2 mean intensity (right panel) at the nuclear envelope in diploid (in grey) and tetraploid (in blue) RPE-1 G1 cells treated with DMSO or with Nocodazole. The same control was used in Fig. 5E because the experiments were done together. Mean ± SEM, >90 G1 cells analysed per condition, three independent experiments. Scale bars: 10 µm. D diploid, T tetraploid, MS mitotic slippage, EnR endoreplication, CF cytokinesis failure, AU arbitrary unit. (**M**) ANOVA test (one-sided). (**A–G**, **I–L**) *t*-test (two-sided). ns not significant.

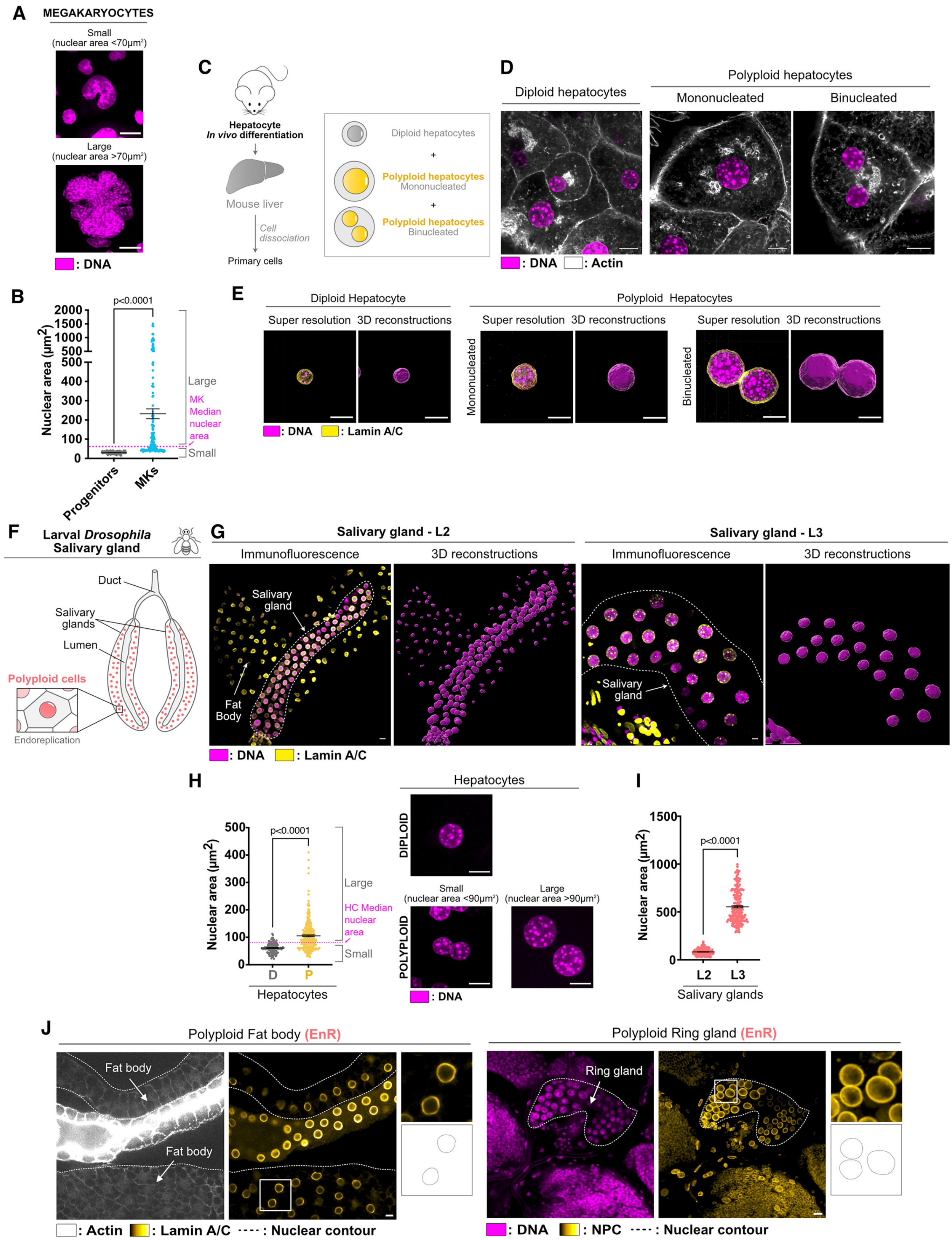
A
MEGAKARYOCYTES
Small
(nuclear area <70µm²)
Large
(nuclear area >70µm²)
: DNA
B
p<0.0001
Nuclear area (µm²)
Large
MK Median nuclear area
Small
Progenitors
MKs
C
Hepatocyte
In vivo differentiation
Mouse liver
Cell dissociation
Primary cells
Diploid hepatocytes
Polyploid hepatocytes Mononucleated
Polyploid hepatocytes Binucleated
D
Diploid hepatocytes
Polyploid hepatocytes
Mononucleated
Binucleated
: DNA : Actin
E
Diploid Hepatocyte
Polyploid Hepatocytes
Super resolution 3D reconstructions
Mononucleated
Binucleated
: DNA : Lamin A/C
F
Larval Drosophila Salivary gland
Duct
Salivary glands
Lumen
Polyploid cells
Endoreplication
G
Salivary gland - L2
Salivary gland - L3
Immunofluorescence
3D reconstructions
Salivary gland
Fat Body
: DNA : Lamin A/C
H
Hepatocytes
p<0.0001
Large
HC Median nuclear area
Small
D
P
DIPLOID
POLYPLOID
Small
(nuclear area <90µm²)
Large
(nuclear area >90µm²)
: DNA
I
p<0.0001
Nuclear area (µm²)
L2
L3
Salivary glands
J
Polyploid Fat body (EnR)
Polyploid Ring gland (EnR)
Fat body
Ring gland
: Actin : Lamin A/C ----: Nuclear contour
: DNA : NPC ----: Nuclear contour

 © The Author(s)

Figure EV4. Physiological polyploid cells generated by cytokinesis failure and endoreplication show homogenous nuclear shape.

(**A**) Representative images of small and large MKs. DNA in magenta. (**B**) Graph showing nuclear area in diploid progenitors and in polyploid MKs. The MK median nuclear area was used to distinguish small and large MKs. Mean ± SEM, >150 interphase cells analysed per condition, three independent experiments. (**C**) Schematic workflow showing the methods used to obtain mouse hepatocytes. (**D**) Representative images showing diploid and polyploid hepatocytes. Actin in grey, DNA in magenta. (**E**) SIM representative images and their corresponding 3D reconstructions showing diploid and polyploid hepatocytes. Lamin A/C in yellow, DNA in magenta. (**F**) Schematic representation of *Drosophila* salivary glands. (**G**) Representative images and their corresponding 3D reconstructions showing polyploid salivary glands. Lamin A/C in yellow, DNA in magenta. (**H**) Left panel—Graph showing the nuclear area in diploid and in polyploid hepatocytes (in grey and yellow, respectively). The hepatocyte median nuclear area was used to distinguish small and large hepatocytes. Mean ± SEM, >140 interphase cells analysed per condition, three independent experiments. Right panel—Representative images showing diploid and polyploid hepatocytes. DNA in magenta. (**I**) Graph showing the nuclear area in polyploid salivary glands at two different developmental stages (L2 and L3). Mean ± SEM, >100 cells analysed, three independent experiments. (**J**) Representative immunofluorescence images showing *Drosophila* fat body (left panel) and ring gland (right panel). Lamin A/C or Nuclear pore complex (NPC) in yellow hot, Actin in grey, DNA in magenta. Scale bars: 10 µm. D diploid. P polyploid, MK megakaryocyte, NPC nuclear pore complex. (**B**, **H**, **I**) *t*-test (two-sided). ns not significant.

© The Author(s)

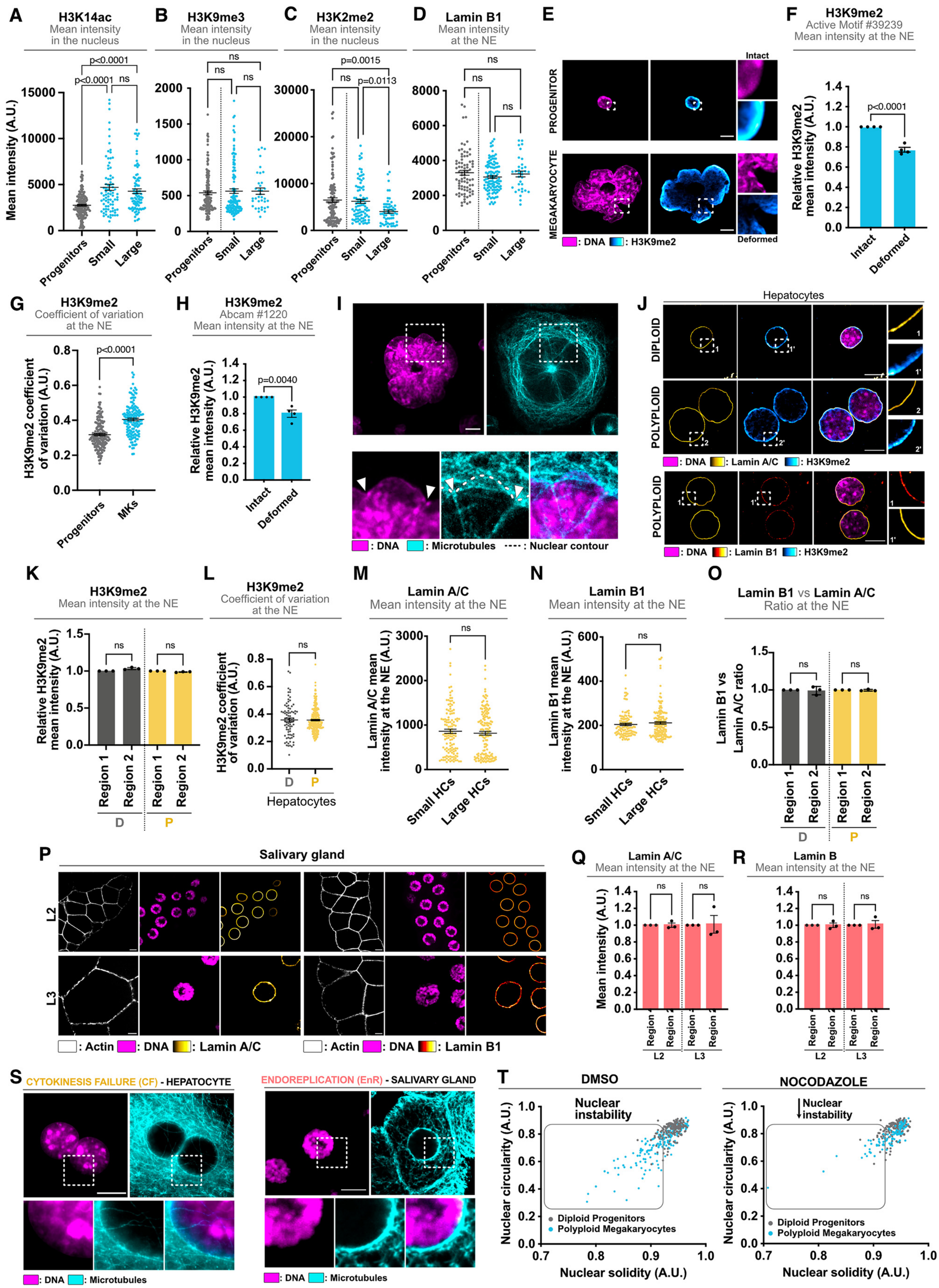
A
H3K14ac
Mean intensity in the nucleus
Mean intensity (A.U.)
p<0.0001
p<0.0001
ns
Progenitors
Small
Large
B
H3K9me3
Mean intensity in the nucleus
C
H3K2me2
Mean intensity in the nucleus
p=0.0015
p=0.0113
D
Lamin B1
Mean intensity at the NE
E
PROGENITOR
MEGAKARYOCYTE
Intact
Deformed
: DNA : H3K9me2
F
H3K9me2
Active Motif #39239
Mean intensity at the NE
Relative H3K9me2 mean intensity (A.U.)
Intact
Deformed
G
H3K9me2
Coefficient of variation at the NE
H3K9me2 coefficient of variation (A.U.)
Progenitors
MKs
H
H3K9me2
Abcam #1220
Mean intensity at the NE
p=0.0040
I
: DNA : Microtubules ----: Nuclear contour
J
Hepatocytes
DIPLOID
POLYPLOID
: DNA : Lamin A/C : H3K9me2
: DNA : Lamin B1 : H3K9me2
K
H3K9me2
Mean intensity at the NE
Region 1
Region 2
D
P
L
H3K9me2
Coefficient of variation at the NE
Hepatocytes
M
Lamin A/C
Mean intensity at the NE
Lamin A/C mean intensity at the NE (A.U.)
Small HCs
Large HCs
N
Lamin B1
Mean intensity at the NE
Lamin B1 mean intensity at the NE (A.U.)
O
Lamin B1 vs Lamin A/C
Ratio at the NE
Lamin B1 vs Lamin A/C ratio
P
Salivary gland
L2
L3
: Actin : DNA : Lamin A/C
: Actin : DNA : Lamin B1
Q
Lamin A/C
Mean intensity at the NE
Mean intensity (A.U.)
R
Lamin B
Mean intensity at the NE
Region
L2
L3
S
CYTOKINESIS FAILURE (CF) - HEPATOCYTE
ENDOREPLICATION (EnR) - SALIVARY GLAND
: DNA : Microtubules
T
DMSO
NOCODAZOLE
Nuclear instability
Nuclear circularity (A.U.)
Nuclear solidity (A.U.)
Diploid Progenitors
Polyploid Megakaryocytes

 © The Author(s)

Figure EV5. Polyploid hepatocytes and salivary gland cells do not show defects in H3K9me2 and Lamin A/C distribution.

(**A–D**) Graphs showing (**A**) H3K27ac, (**B**) H3K9me3 and (**C**) H3K9me2 mean intensity in the nucleus in diploid progenitors (in grey) and in polyploid megakaryocytes (in blue). Mean ± SEM, >120 cells analysed, three independent experiments and Lamin B1 mean intensity at the nuclear envelope (**D**) in diploid progenitors (in grey) and in polyploid megakaryocytes (in blue). (**E**) SIM representative images of diploid progenitors and polyploid megakaryocytes. H3K9me2 in cyan hot, DNA in magenta. (**F**, **G**) Graphs showing H3K9me2 (**F**) mean intensity and (**G**) coefficient of variation (defined as the ratio of the standard deviation to the mean) in progenitors (in grey) and in MKs (in blue). Mean ± SEM, >150 interphase cells analysed per condition, four independent experiments. (**H**) Graph showing H3K9me2 mean intensity in progenitors (in grey) and in MKs (in blue). Mean ± SEM, >65 interphase cells analysed per condition, four independent experiments. (**I**) Representative images of microtubules (in cyan) in MKs, DNA in magenta. (**J**) SIM representative images showing diploid and polyploid hepatocytes. Lamin A/C, Lamin B1 and H3K9me2 in yellow hot, red hot and cyan hot, respectively, DNA in magenta. (**K**, **L**) Graphs showing (**K**) the relative H3K9me2 mean intensity at the NE and (**L**) the H3K9me2 coefficient of variation (defined as the ratio of the standard deviation to the mean) at the NE in diploid (in grey) and in polyploid (in yellow) hepatocytes. Mean ± SEM, >90 interphase cells analysed per condition, three independent experiments. (**M–O**) Graphs presenting (**M**) Lamin A/C, (**N**) and Lamin B1 mean intensity at the NE and (**O**) the Lamin B1 vs Lamin A/C ratio at the NE in hepatocytes. Mean ± SEM, >90 interphase cells analysed per condition, three independent experiments. (**P**) Representative images showing *Drosophila* salivary glands at two different developmental stages (L2 and L3). Lamin A/C and Lamin B1 in yellow hot and red hot, respectively, Actin in grey, DNA in magenta. (**Q**, **R**) Graphs showing (**Q**) Lamin A/C and (**R**) Lamin B1 mean intensity at the NE in *Drosophila* salivary glands. Mean ± SEM, >150 cells analysed per condition, three independent experiments. (**S**) Representative images showing (left panel) polyploid hepatocytes and (right panel) *Drosophila* salivary gland cells. Microtubules in cyan. DNA in magenta. (**T**) Graph presenting the nuclear circularity and solidity index in progenitors (in grey) or megakaryocytes (in blue) treated with DMSO or Nocodazole. Scale bars: 10 µm. D diploid, P polyploid, HC hepatocytes, MK megakaryocytes, NE nuclear envelope. (**D**) ANOVA test (one-sided). (**F**, **G**, **J–O**, **Q**, **R**) *t*-test (two-sided). ns not significant.

© The Author(s)
