# Supplementary material for: Whole genome duplication through mitotic slippage causes nuclear instability
Source: EMBO J. 2026 Sep 19;45(20):7103–35. doi: 10.1038/s44318-026-00912-y (PMC13672370; doi:10.1038/s44318-026-00912-y)
Supplement: Supplementary file 3 [file 44318_2026_912_MOESM2_ESM.pdf]

THE EMBO JOURNAL

Whole genome duplication through mitotic slippage causes nuclear instability

Simon Gemble, Margot Budzyk, Anthony Simon, Ruxandra Lambuta, Luca Nanni, Nicole Weiss, Audrey Forest, Yekaterina Miroshnikova, Federica Scotto Di Carlo, Veronique Marthiens, Corentin Verdel, Jing Fang, Chantal Desdouets, Sara Wickström, Giovanni Ciriello, Elisa Oricchio, Genevieve Almouzni, and Renata Basto

Corresponding author(s): Renata Basto (renata.basto@curie.fr) , Simon Gemble (simon.gemble@curie.fr)

Review Timeline:

Submission Date:	9th Jun 26
Editorial Decision:	31st Jul 26
Revision Received:	28th Aug 26
Accepted:	2nd Sep 26

Editor: Hartmut Vodermaier

Transaction Report:

(Note: With the exception of the correction of typographical or spelling errors that could be a source of ambiguity, letters and reports are not edited. Depending on transfer agreements, referee reports obtained elsewhere may or may not be included in this compilation. Referee reports are anonymous unless the Referee chooses to sign their reports.)

Please note that the manuscript was transferred from another journal where it was originally reviewed. Since the original reviews are not subject to EMBO's transparent review process policy, they cannot be published.

Response to reviewers

In the revised version of our manuscript, which has now been submitted to EMBO Journal and posted on bioRxiv, we have focused on defects in nuclear stiffness as the primary explanation for the observed nuclear deformations. This represents a significant departure from the previous version, in which we considered the phospho-methyl switch as a potential mechanism, without being fully aware of the ongoing controversy surrounding this model in the field.

Our revised interpretation is now supported by robust experimental evidence that was not available at the time of the earlier submission and is presented in the current manuscript. In addition, we have removed the ChIP-seq data and instead provide multiple independent lines of evidence demonstrating that the mislocalization of nuclear envelope components is a consequence of nuclear deformation rather than its cause.

Reviewers' comments:

Reviewer #1 (Remarks to the Author):

Summary

This manuscript from Gemble and colleagues demonstrates that whole genome duplication due to mitotic slippage, where mitosis exit occurs before chromosome segregation, uniquely causes nuclear deformations. Interestingly, the authors demonstrate that the generation of polyploid megakaryocytes also occurs by mitotic slippage, implying that the characteristically irregular shape of megakaryocyte nuclei is due to the abnormalities in nuclear reformation that these authors have uncovered. The authors identify abnormal features of tetraploid nuclei generated by mitotic slippage, including abnormally high phosphorylation of histone H3 at serine 10, altered chromatin modifications, and nuclear softening. This work defines unique features of tetraploid nuclei induced by mitotic slippage and opens doors for future exploration into the potential unique properties of these nuclei, and how these properties may enable the specialized functions of megakaryocytes.

In this reviewer's opinion, the core biology explored here and the major finding - that polyploidy induced by mitotic slippage induces specific nuclear alterations - is of significant interest. I hope that these questions and critiques on both technical and conceptual aspects will help to strengthen this interesting story.

Major Critiques

1. The model and proposed order of events are a bit confusing. From the data presented, it seems clear that 1) mitotic slippage allows mitotic exit with high levels of H3S10 phosphorylation and possible alterations to chromatin state. The relative

timing of the following two observations is less clear: mitotic slippage 2) makes tetraploid nuclei softer and more deformable by microtubules shortly after mitosis and 3) alters lamina and chromatin organization at nuclear deformations. It seems possible to this reviewer that (2) drives (3) rather than the inverse. The statement that "a local decrease in H3K9me2 and Lamin A/C levels that reduces nuclear stiffness facilitating the deformation of the tetraploid nuclei by the microtubule cytoskeleton" (Discussion, line 455) should be re-examined.

We understand this reviewer's comment very well, we have discussed the order of events several times. To address this point in more detail we rescued the nuclear deformations by treating cells with nocodazole or methylstat (Fig 3E + Supp Fig. 7M) and found that Lamin A/C and H3K9me2 levels at the nuclear envelope were comparable to controls. This shows that the local decrease in Lamin A/C and H3K9me2 levels at the nuclear invaginations should be interpreted as a consequence of nuclear deformations rather than a cause.

2. Do all tetraploid cells have softer nuclei, or is this phenomenon specific to those induced by mitotic slippage? AFM analyses are only shown for diploid versus mitotic slippage-induced tetraploid nuclei in Figure 2A-C, but should be repeated on tetraploid nuclei induced by other means to determine whether this effect is specific to mitotic slippage. For many other analyses shown in the paper (e.g. H3S10A expression, methylstat treatment), it is in the opinion of this reviewer reasonable to infer that decreased nuclear deformations imply increased nuclear robustness/stiffness; however, directly testing via AFM whether mitotic slippage specifically induces nuclear softening seems central to the claims of this manuscript.

It is true that we focus our analysis on mitotic slippage induce tetraploidy. Please bear in mind that there are already a huge number of experiments in this paper. Still, to directly address this point, we have performed AFM experiments in tetraploid cells induced by cytokinesis failure and in mitotic slippage cells plus methylstat treatment (Fig. 2J + Supp Fig. 4C). We found that cytokinesis failure does not exhibit changes in nuclear stiffness while methylstat treatment restored nuclear stiffness in mitotic slippage- induced tetraploid cells.

3. The cell cycle dependence of the methylstat rescue phenotype indicates that increasing nuclear stiffness at mitotic exit prevents nuclear deformation, while increasing nuclear stiffness in G1 cannot revert nuclear deformations that have already formed. To confirm that nuclear stiffness is important to prevent deformation from microtubules shortly after mitosis, it would be ideal to measure nuclear stiffness during chromatin decondensation at mitotic exit (if possible), rather than in G1-arrested cells as currently done.

We understand this reviewer's suggestion, but this is technically not possible with AFM. It is worth mentioning that we tried very hard to use magnetic tweezers and

beads without success, as the beads moved very fast away from the nucleus. So, as the reviewer wrote (if possible), we would like just to state that this is not possible.

4. The authors uncover a significant increase in LBR expression in deformed tetraploid nuclei (Supp. Fig. 2C) but do not follow up on this finding. Given the known relationships between LBR expression and nuclear deformability, for instance, in neutrophils (PMID 18621876) and in cancer cells (Baird et al., BioRXiV 2023), LBR may play a major role in deformability of tetraploid nuclei. Have the authors explored this?

We have not attempt to decrease Lamin B receptor (LBR) because of the findings described in point 1 related with local changes at the nuclear deformations. If absolutely required, we can characterize LBR behavior in conditions where nuclear deformations will be prevented, but we consider that this may be redundant to the analysis of H3K9me2 and Lamin already included in this last version of the paper.

The relationships between abnormally elevated H3S10 phosphorylation, diminished H3K9 methylation, and chromatin-lamina interactions are inconclusive. A few related points:

5. While the authors correlate the stage of mitosis where mitotic slippage occurs (prometaphase or metaphase) with high levels of H3S10 phosphorylation (Figure 3A-B), they do not directly show that H3S10 phosphorylation remains on chromatin in newly born tetraploid nuclei after nuclear resealing. A direct demonstration of this is necessary to support their model that persistent H3S10 phosphorylation antagonizes the normal orchestration of nuclear envelope reformation.

We have now included data showing H3S10 positive patches in G1 tetraploid cells generated by mitotic slippage but not by cytokinesis failure (Fig. 2A-B).

6. The authors assert that elevated H3S10 phosphorylation impairs methylation of the nearby H3K9 site, which they refer to as a “phospho-methyl switch”. This phenomenon is described at line 300 by the statement that “high H3S10 phosphorylation levels are accompanied by low H3K9 methylation levels and vice versa”. This statement is a misunderstanding of the literature, which has concluded that H3S10 phosphorylation impairs the recognition of H3K9me2 by antibodies, but has no effect on the levels of H3K9me2 itself (see PMIDs 31573510 and 18835819, both cited by the authors in this manuscript). Instead, H3K9me2 levels remain consistent through mitosis, while H3S10 phosphorylation may alter the ability of H3K9me2 to be bound by endogenous reader and/or tether proteins as well as by recombinant antibodies. This established epitope masking artifact may confound interpretation of the methylstat data shown in Figure 3 F-I as well.

We think that there was a mis-interpretation of our statement in relation with the behaviour of H3K9me2 during mitosis. To facilitate comprehension and to avoid any mis-understanding, we have avoid mentioning the phospho-methyl switch – which, as the reviewer righteously points out remains quite debated in the field. We have now

focused the paper on nuclear stiffness because, as we have explained above, it appears as the main driver of the nuclear deformations. Also, as explained above our data are consistent with the abnormal behavior of H3K9me2 at the nuclear envelope to be explained as a consequence of nuclear deformations rather than a cause (Fig 3E + Supp Fig. 7M).

The authors do clearly show that H3K9me2 levels are locally decreased at invaginations within G1 tetraploid nuclei (Fig. 2D,F), as well as being generally less abundant at the NE in most G1 tetraploid nuclei (Fig. 2E; Supp. Fig. 5E,F). However, it is essential to determine whether this phenotype is also confounded by epitope accessibility artifacts. That is: it is possible that persistent H3S10 phosphorylation impairs recognition of H3K9me2 by antibodies without actually affecting the levels of H3K9me2. Alternatively, if H3S10 phosphorylation has been removed from the chromatin of tetraploid nuclei by the time that H3K9me2 levels are assessed (in G1), epitope masking artifacts are unlikely. This ambiguity could be resolved by costaining or parallel staining for H3S10 phosphorylation and H3K9me2 at the same timepoints in tetraploid nuclei.

Based on the H3S10 patterns observed in G1, it is plausible to conclude that in G1 H3S10 signals are more restricted (Fig. 2A-B) than H3K9me2 signals, which are detected along the nuclear envelope (Last version of the paper: Fig. 3D). Also, we performed co-staining of H3S10 and H3K9me2 in mitosis and we were able to detect both signals.

7. Given the data shown, it is a significant overstatement to claim that “mitotic slippage impairs the interaction between chromatin and nuclear lamins” (heading at line 246). This claim is based on decreased Lamin A and H3K9me2 levels at deformations of tetraploid nuclei (Figure 2). These data do clearly show that lamin A is displaced from regions of high NE curvature while lamin B1 is not. However, immunofluorescence alone cannot determine whether the interactions between chromatin and the nuclear lamina are impaired, as it does not show that chromatin (not just H3K9me2) is detectably displaced from the lamina or that lamin B1 does not tether chromatin in places where lamin A is depleted. To directly assess chromatin:lamina interactions, lamin genome-binding analyses are required. Indeed, the authors have generated H3K9me2/3 ChIP-seq data already (Figure 4); the addition of lamin B1 ChIP-seq would make it possible to determine whether lamin:chromatin interactions are altered at H3K9me2/3 marked domains by mitotic slippage.

We have changed the interpretation related with H3K9me2 and lamin interactions. For these reasons, we consider that Lamin B1 Chip-seq is not essential at this point.

The genomic and transcriptomic analyses could be clarified, strengthened, and more effectively synthesized. Some related points:

8. The authors use spike-in-controlled ChIP-seq to assess H3K9me2/3 levels in diploid versus G1-phase tetraploid cells. According to the Methods, consistent numbers of diploid or tetraploid cells were used for all conditions, as well as a consistent volume of spike-in control. However, because diploid cells are 2n while tetraploid cells are 4n, this means that if a mark is unaffected by tetraploidy, the ratio of ChIPped DNA to spike-in DNA should be approximately 2-fold higher in tetraploid cells than in diploid cells. Did the authors account for this 2-fold factor in their analysis?

We understand this reviewer's request. We have removed the ChIPseq in this new version to simplify the message. In future work Chip-Seq analysis of several histone modifications should be done, but in light of the current results, we think that this is not required to explain the nuclear observations described here.

9. It is not clear whether the "enriched" ChIP-seq peaks shown in Figure 4 were identified by statistically significant enrichment determined by a peak-calling method as is the standard in the field, or alternatively whether the peaks shown are the raw ChIP-seq data. To define insignificant versus significant differential enrichment in diploid versus tetraploid chromatin, biostatistical analyses are also required. Related to this, it is unclear how many replicates were performed for Hi-C and ChIP-seq analyses shown in Figure 4; a minimum of 3 replicates would be included to perform sufficiently powered statistical analyses and make rigorous claims about differential chromatin organization / enrichment of chromatin modifications.

As stated above, we have removed the chip-seq data to facilitate simplicity and comprehension of the model.

10. Without statistically rigorous identification of differential Hi-C and ChIP-seq regions, the authors have missed the opportunity to ask whether their differentially expressed genes in G1 tetraploid cells are significantly enriched in the differential Hi-C and/or ChIP peaks. Synthesis of these datasets would strengthen this portion of the manuscript.

We have performed statistical analysis for HiC and ChIPseq results. We can improve the text description and figures to make ii more straightforward.

11. By comparing diploid to G1 tetraploid cells in Hi-C and ChIP-seq experiments, the authors cannot determine whether chromatin changes observed are shared across all means of tetraploidy induction or are unique to mitotic slippage.

Yes, the reviewer is right, this is exactly what we did. Since this new version of the manuscript is focused on the softness of the nucleus that we compared between mitotic slippage and cytokinesis failure, we can only discuss this point. I hope the reviewers and editors recognize that the amount of work and results here is already difficult to take in. We will focus the discussion only on these tested points.

12. The authors could clarify why they chose to examine gene expression in G1 versus G2 tetraploid cells, since G2 phase cells have not been characterized elsewhere in the manuscript (are they still deformed? Do they still have heterochromatin and lamina alterations? etc). Moreover, it would make more sense to compare their expression data to G1 or G2 tetraploid cells generated by other means rather than to diploid cells, as the authors showed previously that no matter the means of tetraploidy induction, 4n cells have DNA damage and genomic instability during DNA replication that could also affect gene expression. Without this data, it is not possible to conclude that these changes of gene expression are related to mitotic slippage specifically.

Yes, G2 cells are still deformed, and this data is presented in Supp Fig. 1P. We prefer to maintain the comparison between the three methods restricted to data included in the paper. Redoing RNAseq and Hi-C and many other omics data is certainly important and relevant, but this remains out of the scope of this paper and represents work for the years to come. We hope the editors of EMBO J agree with us.

Minor Critiques

1. The manuscript Results section is vague about the methods used to induce polyploidy (although this information is found in the Methods). More explicit clarity on this point would be helpful for the reader, especially at points where different methods to generate mitotic slippage are compared (for instance in Figure 2A-C / discussed at line 184; and Supp. Fig. 4 and discussion at line 235). What method was used for the majority of the experiments shown in the manuscript?

We can certainly improve the text to clarify this specific point. For example add this information in the figure legends?

2. Ideally, scatterplots could be presented as “superplots”, where the mean of each replicate is shown on graphs overlaid with the individual replicate points, to give a clearer picture of reproducibility and statistical significance.

We can do these changes.

3. what do the black, white, gray, and red bars underneath ChIPseq peaks in Figure 4E and F signify?

We have removed the data concerning the Chip-seq. The colors refered to chromosomal regions.

4. The authors show that microtubules induce nuclear deformations shortly after mitosis, and further show that enhancing microtubule stability (via taxol) increases the severity of deformations. Could the persistence of deformations through interphase be due to the stabilization of microtubules by acetylation?

We have characterized microtubule acetylation, but did not observe any major difference. We can include this data if required.

5. Cytokinesis failure also causes the formation of mononucleate tetraploid cells with some frequency. Are these nuclei also deformed? If no, this could also align with especially high H3S10 phosphorylation levels promoting nuclear deformation in mitotic slippage.

Nuclear deformations were never observed in cells after cytokinesis failure -even in mononucleated cells. We can include this information in the paper.

Reviewer #2 (Remarks to the Author):

Review on “Mitotic slippage causes nuclear instability in polyploid cells”.

In a previous landmark paper, the Basto lab induced diploid cells to become tetraploid using three different methods – mitotic slippage (MS), cytokinesis failure or endoreplication. All three methods caused high levels of DNA damage in the whole genome doubled cells due to replication stress. Here, they follow up on that work to identify differences in the downstream nuclear behaviour between MS and the other two methods.

In this new study, the authors noted that tetraploid cells induced by MS led to abnormal nuclear morphology. Increased histone acetylation and reduction in HP1 foci suggested increased open chromatin and resulted in softer nuclei as measured by atomic force microscopy, and this was related to altered nucleus-microtubule interactions (but not nucleus-actin). Treatment with nocodazole or taxol during tetraploidisation decreased or increased nuclear stiffness respectively. They also noted altered LaminA:Lamin B ratios at the nuclear membrane in tetraploid cells caused by MS. This was all affected by Histone H3-S10 phosphorylation status, which was tested in several ways, and overall appears to be a result of a premature release from mitosis before the natural removal of this mark during a normal mitotic exit. They noted altered gene transcription which they associated with altered chromatin organisation. Interventions in the following G1 all seem to fail to rescue these phenotypes, suggesting a short time window after tetraploidisation in which the ‘damage is done’. They then find similar effects to this manipulated system in a natural biological context; murine megakaryocytes which use MS, but not in murine hepatocytes nor drosophila salivary glands which use alternative methods to induce polyploidy.

Overall this manuscript was extremely well written with a lot of careful experiments and controls and is a good continuation of their previous work. All statements are well backed up by the data presented, and major conclusions are based off multiple lines of experimentation. The phenomenon of whole genome doubling (WGD) is of great interest in cancer and developmental biology fields. Many groups induce whole

genome doubled states to model the consequences, and understanding the impact of these methods is important. Furthermore, detailed mechanistic understanding of the cellular 'signature' of specific pathways to whole genome doubling provides a platform to understand the aetiology of this event in cancer, or in normal development.
In my opinion, therefore, and coupled to the high robustness of the presented work, these findings are of great interest to the readership of the journal.

Major points:

1. Induction of tetraploidy results in a mixture of diploid and tetraploid cells. What is the actual success rate of this induction? I.e. it would be helpful to see the percentage of tetraploid cells induced for each method. Relatedly, what are the specific criteria used to distinguish diploid and tetraploid cells? (ie nuclear or cell size). It would help to have provide more details on these aspects rather than only citation of their previous paper in the methods. Additionally, they seem to be using slightly different drugs to their previous paper to induce tetraploidy, why did they switch?

We can include all this information and justification in the paper.

2. The authors perform several experiments to reverse the impact of MS, one at a time (i.e. H3S10 mutated to alanine, or inhibit deacetylase, or the H3S10 kinase, or methylstat to block demethylation) which all give partial rescue of phenotypes. What happens if you combine them in one, do you get stronger rescue? In addition to providing a stronger rescue, this combined manipulation may reveal whether these mechanisms lie in the same, or distinct pathways. This is just a suggestion.

The rescue using methylstat is very impressive- to levels compared to control- suggesting a strong contribution from nuclear stiffness. However, we also know that it is not sufficient since when we reduced nuclear stiffness in diploid cells we do not observe nuclear deformations (we can add this set of experiments to the paper- Diploid cells treated with TSA to increase histone acetylation does not result in nuclear deformations).
We favor a model where the nuclear deformations result from a combination of decreased nuclear stiffness and microtubule cytoskeleton re-organisation. We can include this data and explain our rationale. Further, we can also combine H3S10A and nocodazole treatment, if required.

3. In Figure 4, the authors noted altered methylation, chromatin organisation and then transcription in tetraploid vs diploid, but the data is only from the MS tetraploids. Did they study the other two types of tetraploidisation? I think it is important to report findings from the other methods as a comparison for this method of tetraploidisation specifically, even if just for the gene expression analysis. For example, it is hardly surprising that transcript programs are altered upon tetraploidy in general, so is it also altered in the other tetraploidy methods? Additionally, is this expression alteration maintained after subsequent cell cycles or this is only after immediate tetraploidisation?

This question is similar to the question raised by the previous reviewer. We understand that a full comparison of RNAseq, HiC and even other omics approaches will be very useful to fully grasp possible communalities and differences between all the ways of undergoing whole genome duplication. But here, this study focus on mitotic slippage and its consequences. We hope therefore that the reviewers and the editors agree with our strategy of comparing several parameters but not all. It is important to mention that in the revised version, we are submitting here, we have expanded the analysis to other histone marks such as H3K9me3/H3K27me3 and H3K9ac/H3K27ac to all methods of inducing whole genome doubling (Fig. 3A-B +Supp Fig. 3G-H).

4. The authors find their results are duplicated in “natural MS” megakaryocytes and not in drosophila salivary glands or mouse hepatocytes (which do not use MS but rather cytokinesis failure or endoduplication). Are they examining this at the first tetraploidisation event? Or are these cells are already polyploid compared to the RPE1 in vitro experiments which went from diploid to tetraploid. What is the actual ploidy of the in vivo cells, does that play any role on top of the method to induce polyploidy? Could they provide a FACS profile to clarify this?

We are not examining megakaryocytes and salivary glands in their first cell cycle following mitotic slippage or endoreplication. This would be quite difficult to do for megakaryocytes, *in vitro* primary cultures. For *Drosophila* salivary glands, we have studied these for another project in the lab within the context of Drosophila embryogenesis. We know that endoreplication is initiated in the embryo and these nuclei do not show nuclear deformations. We will explain this better in the text.

5. Does the nuclear instability lead to any genomic instability? Ie DNA damage, genomic alterations? In their last publication replication stress was induced but it is not clear in this new manuscript whether this nuclear instability directly contributes further to genomic instability? I think the field would be very interested to know if this method of tetraploidisation specifically is likely to cause damage to the genome in addition to replication stress caused by WGD generally.

This is of course a very interesting question. In our previous study, we have indeed shown that all the methods employed to induce tetraploidy in RPE-1 cells generate replicative stress and DNA damage. However, in what concerns mitotic slippage, we have observed an inverse correlation between DNA damage and nuclear deformations suggesting that the nuclear deformations are not responsible for increasing DNA damage levels. We would be happy to include this data in the manuscript.

Minor points:

6. Could the abbreviations (MS, EdR etc) be added to Fig. 1A?
7. Line 259: typo in mean (should be means)

8. Line 290 typo (impact should be impacts or impacted)
9. Line 309 typo: improve should be improves

We have corrected these points.

Reviewer #3 (Remarks to the Author):

The paper by Gemble et al is about very important question in biology – how genome duplication and potentially polyploidy is achieved in both physiological and pathological contexts. The authors consider and compare three possible paths towards polyploidization – mitotic slippage, cytokinesis failure and endoreplication and show that the only one of the paths, mitotic slippage, leads to nuclear abnormalities, such as deformed nuclear shape, changes in histone modifications, and altered transcription.
The authors mostly used cultured cells with induced tetraploidy and microscopy after immunostaining as a readout followed by meticulous quantification and statistical analysis. Two important criteria of nuclear deformation are used by the authors - the nuclear circularity and the nuclear solidity, both of which decrease in tetraploid nuclei after mitotic slippage. The authors employed many experimental treatments leading to inhibition or activation of certain enzymes. The microscopy analysis is supplemented with Hi-C, analysis, ChIPseq and RNAseq. The manuscript is well written and meticulously illustrated.
The major conclusion of the work is that after mitotic slippage, cells undergoing high deformation of the nucleus resulting from an increased interaction with microtubules on the background of decreased nuclear stiffness and alteration of the nuclear envelope and peripheral chromatin properties.
I have to acknowledge that the authors undertook a colossal work on the level of microscopy analysis. What I especially praise, they used tissues and cell types with naturally occurring polyploidization for comparison.
The more it is hard for me to state that most of their work is based on the artifactual immunostainings and false rationale.
============================

(1) The main conclusion of the manuscript is that “MS-induced tetraploid cells fail to establish links between H3K9me2 chromatin and the Lamin A/C increasing nuclear deformability by the microtubule cytoskeleton”. The conclusion is based on the weakened or even absent staining of H3K9me2 using AM39239 antibody from Active Motif. However, the staining is artifactual, because this antibody is trapped at the very nuclear periphery, does not penetrate into nuclear interior and, hence, does not stain nucleolar periphery and other chromatin loci, including chromatin in deep nuclear invaginations. This antibody-trapping phenomenon is caused by combination of two factors, high antigen abundance and high antibody affinity. By now, this phenomenon is well known in the community and there is even the BioRxiv paper (10.1101/2025.04.09.648027), in which the authors use exactly the same anti-H3K9me2 AM39239 as an example, but also describe many other cases. I do not remember whether they used lamin A/C (SAB4200236, Sigma) but it might also show trapping effect.
I do not blame the authors who are unaware of this artifact. They used the antibody suggested in Ref.33 by Poleshko et al. Indeed this group used the same Active Motif antibody in several of their publications. The authors have not recognized the artifact,

and presumably were unaware that they publish false data.
Therefore, Figures 2D, 3E,J, 6B,J and many other panels in Supplemental figures show an artifactual peripheral staining and should not be published. Correspondingly, the summarizing schematics on Figs. 2K and 6L are incorrect.

We were not aware of this problem with the antibody and we thank this reviewer for pointing this out for us. However, we do not think that the data is artefactual as we used another antibody (cited as the good one in the bioRxiv paper) and tested another fixation protocol. We observed exactly the same local decrease at the local invaginations after mitotic slippage in RPE-1 cells and in megakaryocytes (Supp Fig. 7A-C + Supp Fig. 10H).

(2) The authors state that "…high H3S10 phosphorylation levels are accompanied by low H3K9 methylation levels and vice versa, creating a "phospho/methyl switch 33,52,53". The reference 53, however, states that absence of H3K9 methylation is an artifact caused by inaccessibility of antibodies and by histone extraction using SDS buffer. Moreover, it is a common knowledge nowadays that missing H3K9me3/2/1 immunostaining signals from prophase until early anaphase is due to epitope occlusion, since this is the exact time when the adjacent S10 is phosphorylated. I do not have a reference at hand, but many researchers know that some antibodies do not bind when the phospho-mark is abundant on chromatin.
Therefore, the so-called phospho/methyl switch is another artifact, on which the authors build their conclusions.

We have revised the manuscript and removed the phospho-methyl switch in the new version.

(3) However, several observation and conclusions do not depend on artifactual staining. Thus, that the authors convincingly show that upon mitotic slippage (i) tetraploid nuclei have decreased stiffness that coincides or might be caused by increased chromatin acetylation level; (ii) convoluted nuclear shape is somehow connected to interaction of nuclei with microtubules; (iii) nuclear deformations coincide with increased H3S10 phosphorylation and methylation of H3K9. Although, any mechanistic connections between these events and their causal sequence remain unclear, these are interesting facts.

In the revised version of our manuscript, we have included additional data, which allow us to conclude that the local changes observed are the consequence of the nuclear deformations and not the opposite. Please take into account, the results showing that upon methylstat treatment, increased nuclear stiffness can be observed. Even if microtubules are still accumulating at the nuclear envelope, deformations were no longer observed. These results were observed by live imaging approaches and we will be happy to include them in the revised version of the paper. Altogether, this strongly suggest that the two behaviors are independent.

=============================

There are some minor comments that might help the authors in the future:

(1) The authors described increased levels of H3K9ac, H3K27ac and H3.1/2 in tetraploid nuclei. I do not find it surprising because they measure signal intensity of the 3D projection after duplication of the genome. For a proper comparison, they would need to use single confocal sections and use the same ROI at different conditions.

We used the standard methods of calculating mean fluorescence intensities of Z projections, that take into account the area being measured. We can include single sections to provide additional measurements.

(2) Nuclear envelope (NE) segmentation is very crucial for this manuscript, because a lot of conclusions come from measurements “at the NE”. Unfortunately, the method of NE segmentation is not properly described and the authors only write “using the “segmented lines” tool from Image J V2.1.0/1.53c software”. On what staining the segmentation was based? Did they always stained nuclei for lamin B or lamin A/C?

When possible we labelled Lamin A/C and Lamin B1. The nuclear envelope measurement was performed through segmentation of the nuclear periphery in the DAPI channel to avoid any bias measurement of abnormal nuclear envelopes. We are confident that this allows for nuclear envelope measurements since in these cells, the DNA is closely associated with the Lamina network. We can include measurements to show colocalization between the lamin signals and the DAPI periphery through line scans to illustrate this point.

Dr. Renata Basto
Institut Curie
UMR144
12 rue Lhomond
Paris 75005
France

31st Jul 2026

Re: EMBOJ-2026-124882
Whole genome duplication through mitotic slippage causes nuclear instability

Dear Renata,

Thank you again for transferring your previously reviewed and revised manuscript to The EMBO Journal. We decided to send it back to two of the original referees, who have now returned the reports copied below. Both of them considered the work substantially improved and now in principle suitable for publication, but referee 2 still retains a few issues that would require additional modifications in a final round of minor revision; in this regard, please also refer to the attached PDF, in which I made further comments on your response to the previous reviews!

In addition, I would ask you at this stage to also address a number of editorial points as follows (please also consider our more detailed online Guide to Authors):

GENERAL:

- Please download and complete our author checklist (link provided below).

- Please upload the manuscript text (including figure legends) as an editable text file, and all main figures without legends as individual image files with sufficient resolution/quality for production.

- Please provide suggestions for a short 'blurb' text prefacing and summing up the conceptual aspect of the study in two sentences (max. 250 characters), followed by 3-5 one-sentence 'bullet points' with brief factual statements of key results of the paper; they will form the basis of an editor-written 'Synopsis' accompanying the online version of the article. Please also upload a synopsis image, which can be used as a "visual title" for the synopsis section of your paper (maybe utilizing Fig 7E for this purpose?)). The image should be in JPG format, and please make sure that it remains useful/intelligible in the modest dimensions of (exactly) 550 pixels wide and 300 pixels high.

TEXT:

- Please adjust the order as well as the headers of the different manuscript sections: Title page with complete author information, Abstract, Introduction, Results, Discussion, Methods, Data Availability, Acknowledgements, Disclosure and Competing Interests Statement, References, Main Figure Legends, Main Tables, Expanded View Figure Legends

- On the title page, all author names should be given in full - e.g. 'Simon Gemble', 'Sara A Wickström'

- As we have switched from a free-text author contribution statement towards a more formal statement based on Contributor Role Taxonomy (CRediT) terms, please remove the present Author Contribution section and instead specify each author's contribution(s) directly in the Author Information page of our submission system during upload of the final manuscript. See https://casrai.org/credit/ for more information.

- Please rename the Conflict of Interest section into "Disclosure and Competing Interests Statement", in accordance with our updated Guide to Authors (https://www.embopress.org/competing-interests)

- Please include a dedicated "Data Availability" section at the end of the Material and Methods (suggested wording: "The [structural coordinates | microarray | mass spectrometry] data from this publication have been deposited to the [name of the database] database [URL] and assigned the identifier [accession | permalink | hashtag].") This section should also mention any deposited code. Note that only datasets newly generated during the course of this study should be listed here; should there no data deposition to public repositories linked to the study, this should still be stated as "This study includes no data deposited in external repositories."

- Please adjust the format of the reference list and of the in-text citations according to EMBO Journal format (alphabetical order,

author name et al + year, first up to 10 authors should be listed, followed by 'et al'); and also make sure that all citations are complete with journal title, journal volume, page/elocator numbers (DOI info only in case of advance publications that do not yet have volume/page numbers). Furthermore, for preprint citations: Please use in-text referencing according to the format "preprint: Miller et al, 2025"; and in the reference list, continue after the manuscript title with the name of the preprint platform plus DOI + preprint label - e.g. "bioRxiv doi: 1234/002.dfj123 [PREPRINT]". Finally, all references (also for the methods part) should be in one single section.

- Please double-check to make sure to all relevant funding information in the manuscript is congruent with the info entered into our submission system.There are a number of funders acknowledged in the manuscripts that have not been entered into our submission system, in particular: European Research Council (ERC-2015-ADG-694694 ChromADICT), the Ligue Nationale contre le Cancer (Equipe labellisée Ligue); (ANR-11-LABX-0044_DEEP, ANR-10-IDEX-0001-02 PSL); FRM ...

- Please note that Materials and Methods need to be described in the main text using our 'Structured Methods' format: The in-text "Methods" section should contain method and protocol descriptions (ideally using a step-by-step protocol format to facilitate adoption of the methodologies across labs), while all key reagents, experimental models, software and relevant equipment - including their sources and relevant identifiers - should be listed in a separately uploaded Reagents and Tools Table, the template for which can be downloaded at https://media.springernature.com/original/springer-cms/rest/v1/content/27825802/data/v1.

ll Materials and Methods need to be described in the main text using our 'Structured Methods' format. The Methods section should include a (separately uploaded) Reagents and Tools Table (downloadable at https://www.embopress.org/page/journal/14693178/authorguide#structuredmethods) listing key reagents, experimental models, software and relevant equipment, and including their sources and relevant identifiers; and a part describing the methods (ideally using a step-by-step protocol format to facilitate adoption of the methodologies across labs).

- Please make sure to call out each figure and each panel at least once from the main text - several panels (e.g. Fig 2ILM, Fig 3F, Fig 4GH) are currently missing references. Also, double-check that only existing panels are called out (e.g. there seems to be reference to a non-existing Fig. 3H)

DATA:

- Please refer to our author guide (www.embopress.org/page/journal/14602075/authorguide#expandedview) regarding "supplementary figures", which should become either Expanded View Figures our go into an Appendix PDF. I would prefer them to be turned into the former (naming/referencing: 'Figure EV1/2/3...'), but since there are currently only 5 main figures, please consider promoting some of the data in current figures 'S1-S10' into additional main figures - we have no strict limits on this.

- We note that none of the videos/movies referenced in text have already been uploaded. Please do so, making sure to renaming them as Expanded View movies (in-text callouts and legend headers: "Movie EV1/2/..."). Furthermore, their legends should be moved out of the text into individual text files, each of which should be combined with the respective movie file into one separate ZIP file per EV Movie, and uploaded as such.

- During routine pre-acceptance checks, our data editors have raised the following queries regarding figures, data, and legends; I would appreciate if you briefly answered to them in the cover letter of your final submission, and made the requested text modifications with changes/additions highlighted via the "Track changes" option, to facilitate our final checking":
- Please provide the exact p values in the legends of figures 1F, H, I; 2B, D, G, J, L, M; 3C, E; 4D, F; 5B, D, F
- Please define the box plots in terms of centre in the legend of figure 2J
- Please note that information related to n is missing in the legend of figure 2H
- Please define the blue asterisk in the legend of figure 1G.
- Please define the 1B arrow heads in the legend of figure.

- Finally, you shall also receive a separate message from our Source Data curation team, with instructions on how to prepare and upload relevant image and numerical raw data.

I am returning the manuscript to you now for the final revisions, with the hyperlink below allowing you to upload all files once you are ready. Should you need additional guidance/feedback regarding this final adjustments, please do not hesitate to contact us directly. Thank you again for the opportunity to consider this work for The EMBO Journal, and I look forward to receiving your final version!

With kind regards,

Hartmut

Hartmut Vodermaier, PhD

Senior Editor, The EMBO Journal
h.vodermaier@embojournal.org

*** PLEASE NOTE: All revised manuscript are subject to initial checks for completeness and adherence to our formatting guidelines. Revisions may be returned to the authors and delayed in their editorial re-evaluation if they fail to comply to the following requirements. As a first step please read our guidelines for revised submissions: https://link.springer.com/journal/44318/submission-guidelines#cms-Revised-submissions

1) Every manuscript requires a Data Availability section (even if only stating that no deposited datasets are included). Primary datasets or computer code produced in the current study have to be deposited in appropriate public repositories prior to resubmission, and reviewer access details provided in case that public access is not yet allowed.

2) Each figure legend must specify
- size of the scale bars that are mandatory for all micrograph panels
- the statistical test used to generate error bars and P-values
- the type error bars (e.g., S.E.M., S.D.)
- the number (n) and nature (biological or technical replicate) of independent experiments underlying each data point
- Figures may not include error bars for experiments with n<3; scatter plots showing individual data points should be used instead.

3) Revised manuscript text (including main tables, and figure legends for main and EV figures) has to be submitted as editable text file (e.g., .docx format). We encourage highlighting of changes (e.g., via text color) for the referees' reference.

4) Each main and each Expanded View (EV) figure should be uploaded as individual production-quality files (preferably in .eps, .tif, .jpg formats). For suggestions on figure preparation/layout, please refer to our Figure Preparation Guidelines: https://media.springernature.com/original/springer-cms/rest/v1/content/27825798/data/v1

5) Point-by-point response letters should include the original referee comments in full together with your detailed responses to them (and to specific editor requests if applicable), and also be uploaded as editable (e.g., .docx) text files.

6) Please complete our Author Checklist, and make sure that information entered into the checklist is also reflected in the manuscript; the checklist will be available to readers as part of the Review Process File.

7) All authors listed as (co-)corresponding need to deposit, in their respective author profiles in our submission system, a unique ORCiD identifier linked to their name. Please see our Guide to Authors for detailed instructions.

8) Please note that supplementary information at EMBO Press has been superseded by the 'Expanded View' for inclusion of additional figures, tables, movies or datasets; with up to five EV Figures being typeset and directly accessible in the HTML version of the article.

9) To facilitate reproducibilty and cross-laboratory adoption of methodologies, please structure the Materials & Methods section as outlined in our guide to authors, including a completed Reagents and Tools Table.

10) Digital image enhancement is acceptable practice, as long as it accurately represents the original data and conforms to community standards. If a figure has been subjected to significant electronic manipulation, this must be clearly noted in the figure legend and/or the 'Materials and Methods' section. The editors reserve the right to request original versions of figures and the original images that were used to assemble the figure. Finally, we generally encourage uploading of numerical as well as gel/blot image source data.

At EMBO Press we ask authors to provide source data for the main manuscript figures. You will receive a separate email with instructions for providing source data with your revised manuscript, including how to upload and organize the files.

In the interest of ensuring the conceptual advance provided by the work, we recommend submitting a revision within 3 months (29th Oct 2026). Please discuss the revision progress ahead of this time with the editor if you require more time to complete the revisions. Use the link below to submit your revision:

Link Not Available

Referee #1:

This manuscript from Gemble and colleagues demonstrates that whole genome duplication due to mitotic slippage, where mitosis exit occurs before chromosome segregation, uniquely causes nuclear deformations. Interestingly, the authors demonstrate that the generation of polyploid megakaryocytes also occurs by mitotic slippage, implying that the characteristically irregular shape of megakaryocyte nuclei is due to the abnormalities in nuclear reformation that these authors have uncovered. The authors identify abnormal features of tetraploid nuclei generated by mitotic slippage, including abnormally high phosphorylation of histone H3 at serine 10, altered chromatin modifications, and nuclear softening. This work defines unique features of tetraploid nuclei induced by mitotic slippage and opens doors for future exploration into the potential unique properties of these nuclei, and how these properties may enable the specialized functions of megakaryocytes.

I reviewed a previous version of this paper and remain excited by the core biology explored here and the major finding - that polyploidy induced by mitotic slippage induces specific nuclear alterations - which is of significant interest. I applaud the authors for the extensive work they undertook to reframe the manuscript and re-focus it on nuclear softening in response to previous critiques.

Referee #2:

I have indicated our specific comments on the previous reviewer comments (as comments on the word document). Overall, we are happy with the revisions, apart from a few minor comments/suggestions (below) and I do not think any further experiments are required.

Minor comments:
1. It is still a bit unclear about the proposed hierarchy of the phenotypes - the retained phosphomark, altered methylation patterns and microtubule invaginations causing nuclear morphology and stiffness defects. Can the authors present a likely order of events that could be tested in future studies?

2. Methylation marks - one kind (H3K9me2) is specifically increased in MS and not diploid of other WGD methods - but what is the significance? They say methylation generally associated with closed chromatin and their other experiments point to this so how does this increase in H3K9me2 fit in?

3. The Hi-C and RNAseq data are not very informative and not fully explored. If they could relate specific gene expression or genome contacts with locations where invaginations are for example this data could be useful, but as it is, and without comparison to other methods of WGD induction this section is a bit uninformative. In particular, a very superficial analysis of gene expression changes is presented.

4. The purpose of such a mechanism (of nuclear instability upon mitotic slippage) is only briefly touched upon at the end of the discussion - gene expression changes that might sustain the megakaryocyte differentiation for example. Although I am not proposing the authors perform additional experiments at this stage, could they glean any information from the gene expression changes seen in the human induced MS model?

Referee #1:

This manuscript from Gemble and colleagues demonstrates that whole genome duplication due to mitotic slippage, where mitosis exit occurs before chromosome segregation, uniquely causes nuclear deformations. Interestingly, the authors demonstrate that the generation of polyploid megakaryocytes also occurs by mitotic slippage, implying that the characteristically irregular shape of megakaryocyte nuclei is due to the abnormalities in nuclear reformation that these authors have uncovered. The authors identify abnormal features of tetraploid nuclei generated by mitotic slippage, including abnormally high phosphorylation of histone H3 at serine 10, altered chromatin modifications, and nuclear softening. This work defines unique features of tetraploid nuclei induced by mitotic slippage and opens doors for future exploration into the potential unique properties of these nuclei, and how these properties may enable the specialized functions of megakaryocytes.

I reviewed a previous version of this paper and remain excited by the core biology explored here and the major finding - that polyploidy induced by mitotic slippage induces specific nuclear alterations - which is of significant interest. I applaud the authors for the extensive work they undertook to reframe the manuscript and re-focus it on nuclear softening in response to previous critiques.

We thank the reviewer for these positive comments on the new version of the manuscript.

In the previous revision, the reviewer 1 raised the following point to which we would like to respond:

4. The authors show that microtubules induce nuclear deformations shortly after mitosis, and further show that enhancing microtubule stability (via taxol) increases the severity of

deformations. Could the persistence of deformations through interphase be due to the stabilization of microtubules by acetylation?

We characterized microtubule acetylation but observed no significant differences between diploid and tetraploid cells (Figure for reviewer only 1B-C), suggesting that microtubule acetylation does not contribute to nuclear deformations in newly formed tetraploid cells.

Referee #2:

I have indicated our specific comments on the previous reviewer comments (as comments on the word document). Overall, we are happy with the revisions, apart from a few minor comments/suggestions (below) and I do not think any further experiments are required.

Minor comments:

1. It is still a bit unclear about the proposed hierarchy of the phenotypes - the retained phosphomark, altered methylation patterns and microtubule invaginations causing nuclear morphology and stiffness defects. Can the authors present a likely order of events that could be tested in future studies?

We agree with the reviewer. To address this point specifically, we expanded the text related with this point. This can be found in the new version of the manuscript (page 12, line 361). Briefly, based on our new results showing that preventing nuclear deformations was sufficient to prevent changes in nuclear envelope composition, we proposed that these changes are most likely a consequence of the nuclear deformations by the microtubules rather than a cause.

2. Methylation marks - one kind (H3K9me2) is specifically increased in MS and not diploid of other WGD methods - but what is the significance? They say methylation generally

associated with closed chromatin and their other experiments point to this so how does this increase in H3K9me2 fit in?

The fact that H3K9me2 levels are higher in tetraploid cells is quite surprising. However, we believe this does not contribute to the nuclear deformations. Indeed, treating cells with methylstat—which promotes methylation—increases H3K9me2 levels in diploid cells (Figure for reviewer only 1A). However, this treatment does not result in nuclear deformations (new Figure 3I). Moreover, we observed a decrease in H3K9me2 levels with ploidy in megakaryocytes (new Figure EV5C). Together, these data suggest that increased H3K9me2 levels do not contribute to the nuclear instability we observed upon MS.

3. The Hi-C and RNAseq data are not very informative and not fully explored. If they could relate specific gene expression or genome contacts with locations where invaginations are for example this data could be useful, but as it is, and without comparison to other methods of WGD induction this section is a bit uninformative. In particular, a very superficial analysis of gene expression changes is presented.

We thank the reviewer for this comment. We have removed the RNA-seq data (originally shown in Fig. S8H) from the revised manuscript. The number of deregulated genes upon mitotic slippage was too small to support GO term analysis with sufficient confidence, and further interpretation of this dataset would have required additional experiments beyond the scope of this revision. We therefore chose to remove it rather than retain an underpowered analysis.

However, we are convinced that the Hi-C data should remain in the manuscript, as this set of data demonstrate that nuclear deformations impact nuclear organization and 3D genome organization. We have therefore maintained the Hi-C data in the revised manuscript (new Fig. 5F) but moved some of the related data to Appendix Figures (new Appendix Figure S3F-I).

4. The purpose of such a mechanism (of nuclear instability upon mitotic slippage) is only briefly touched upon at the end of the discussion - gene expression changes that might

sustain the megakaryocyte differentiation for example. Although I am not proposing the authors perform additional experiments at this stage, could they glean any information from the gene expression changes seen in the human induced MS model?

Understanding the role of mitotic slippage-induced nuclear deformations in cellular function is crucial. However, we believe this is beyond the scope of the current study. Nevertheless, based on the reviewer's comment, we have expanded on this point in the revised "Discussion" section (Page 17, line 521).

Figure for reviewer only

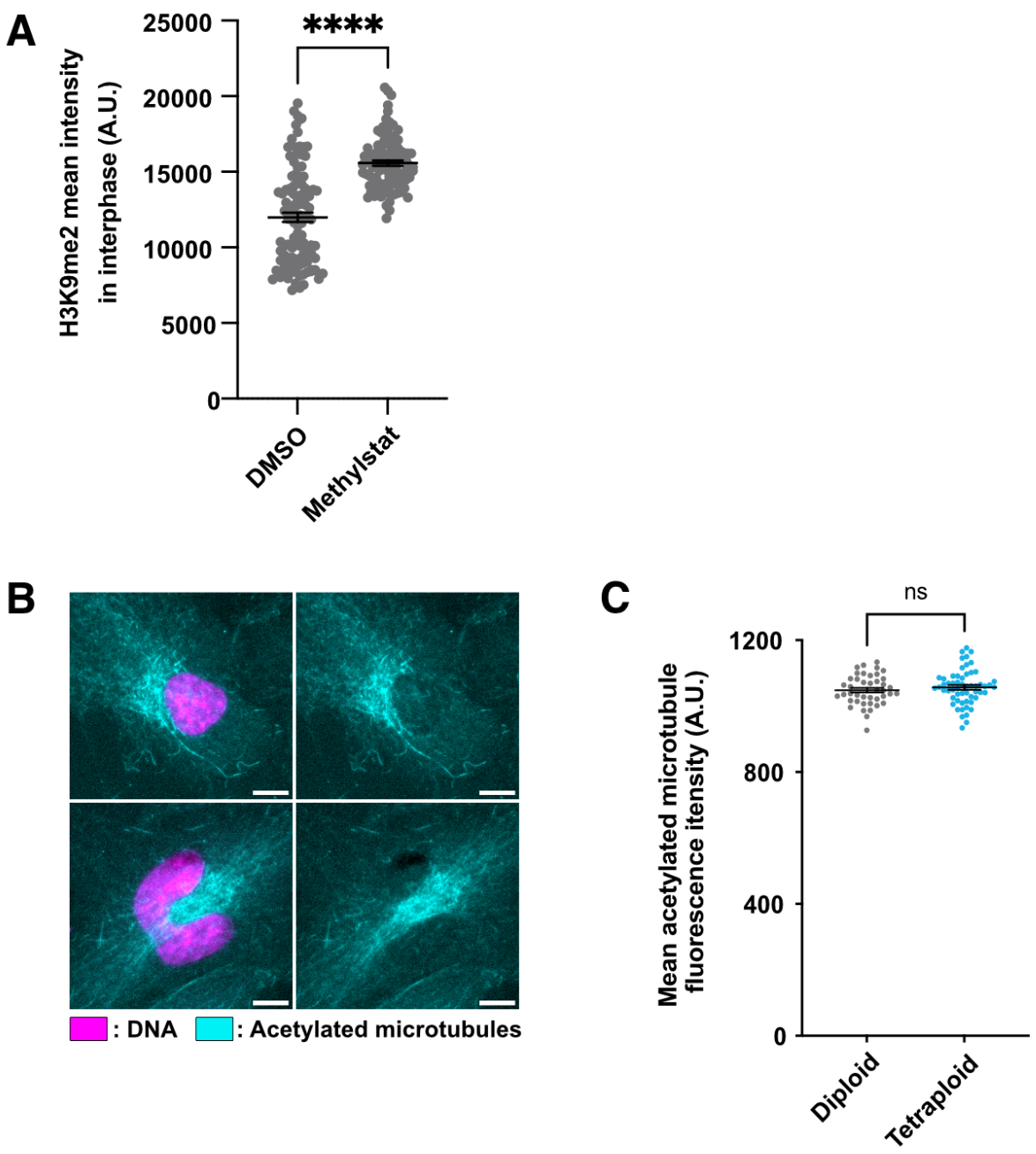

EMBO Press Author Checklist

Corresponding Author Name: Renata Basto	
Journal Submitted to: The EMBO Journal	
Manuscript Number: EMBOJ-2026-124882	

USEFUL LINKS FOR COMPLETING THIS FORM
The EMBO Journal - Author Guidelines
EMBO Reports - Author Guidelines
Molecular Systems Biology - Author Guidelines
EMBO Molecular Medicine - Author Guidelines

Reporting Checklist for Life Science Articles (updated January
This checklist is adapted from Materials Design Analysis Reporting (MDAR) Checklist for Authors. MDAR establishes a minimum set of requirements in transparent reporting in the life sciences (see Statement of Task: 10.31222/osf.io/9sm4x). Please follow the journal's guidelines in preparing your
Please note that a copy of this checklist will be published alongside your article.

Abridged guidelines for figures
1. Data
The data shown in figures should satisfy the following conditions:
- ➞ the data were obtained and processed according to the field's best practice and are presented to reflect the results of the experiments in an accurate and unbiased manner.
- ➞ ideally, figure panels should include only measurements that are directly comparable to each other and obtained with the same assay.
- ➞ plots include clearly labeled error bars for independent experiments and sample sizes. Unless justified, error bars should not be shown for technical
- ➞ if n<5, the individual data points from each experiment should be plotted. Any statistical test employed should be justified.
- ➞ Source Data should be included to report the data underlying figures according to the guidelines set out in the authorship guidelines on Data

2. Captions
Each figure caption should contain the following information, for each panel where they are relevant:
- ➞ a specification of the experimental system investigated (eg cell line, species name).
- ➞ the assay(s) and method(s) used to carry out the reported observations and measurements.
- ➞ an explicit mention of the biological and chemical entity(ies) that are being measured.
- ➞ an explicit mention of the biological and chemical entity(ies) that are altered/varied/perturbed in a controlled manner.
- ➞ the exact sample size (n) for each experimental group/condition, given as a number, not a range;
- ➞ a description of the sample collection allowing the reader to understand whether the samples represent technical or biological replicates (including how many animals, litters, cultures, etc.).
- ➞ a statement of how many times the experiment shown was independently replicated in the laboratory.
- ➞ definitions of statistical methods and measures:
  - common tests, such as t-test (please specify whether paired vs. unpaired), simple χ2 tests, Wilcoxon and Mann-Whitney tests, can be unambiguously identified by name only, but more complex techniques should be described in the methods section;
  - are tests one-sided or two-sided?
  - are there adjustments for multiple comparisons?
  - exact statistical test results, e.g., P values = x but not P values < x;
  - definition of 'center values' as median or average;
  - definition of error bars as s.d. or s.e.m.

Please complete ALL of the questions below.
Select "Not Applicable" only when the requested information is not relevant for your study.

Materials

Newly Created Materials	Information included in the manuscript?	In which section is the information available? (Reagents and Tools Table, Materials and Methods, Figures, Data Availability Section)
New materials and reagents need to be available; do any restrictions apply?	Yes	Material and methods

Antibodies	Information included in the manuscript?	In which section is the information available? (Reagents and Tools Table, Materials and Methods, Figures, Data Availability Section)
For **antibodies** provide the following information:<br>- Commercial antibodies: RRID (if possible) or supplier name, catalogue number and or/clone number<br>- Non-commercial: RRID or citation	Yes	Material and methods

DNA and RNA sequences	Information included in the manuscript?	In which section is the information available? (Reagents and Tools Table, Materials and Methods, Figures, Data Availability Section)
**Short novel DNA or RNA including primers, probes**: provide the sequences.	Not Applicable	

Cell materials	Information included in the manuscript?	In which section is the information available? (Reagents and Tools Table, Materials and Methods, Figures, Data Availability Section)
**Cell lines:** Provide species information, strain. Provide accession number in repository **OR** supplier name, catalog number, clone number, and/**OR** RRID.	Yes	Material and methods
**Primary cultures:** Provide species, strain, sex of origin, genetic modification status.	Yes	Material and methods
Report if the cell lines were recently **authenticated** (e.g., by STR profiling) and tested for mycoplasma contamination.	Yes	Material and methods

Experimental animals	Information included in the manuscript?	In which section is the information available? (Reagents and Tools Table, Materials and Methods, Figures, Data Availability Section)
**Laboratory animals or Model organisms:** Provide species, strain, sex, age, genetic modification status. Provide accession number in repository **OR** supplier name, catalog number, clone number, **OR** RRID.	Yes	Material and methods
**Animal observed in or captured from the field:** Provide species, sex, and age where possible.	Not Applicable	
Please detail **housing and husbandry conditions**.	Yes	Material and methods

Plants and microbes	Information included in the manuscript?	In which section is the information available? (Reagents and Tools Table, Materials and Methods, Figures, Data Availability Section)
**Plants:** provide species and strain, ecotype and cultivar where relevant, unique accession number if available, and source (including location for collected wild specimens).	Not Applicable	
**Microbes:** provide species and strain, unique accession number if available, and source.	Not Applicable	

Human research participants	Information included in the manuscript?	In which section is the information available? (Reagents and Tools Table, Materials and Methods, Figures, Data Availability Section)
If collected and within the bounds of privacy constraints report on age, sex and gender or ethnicity for all study participants.	Not Applicable	

Core facilities	Information included in the manuscript?	In which section is the information available? (Reagents and Tools Table, Materials and Methods, Figures, Data Availability Section)
If your work benefited from core facilities, was their service mentioned in the acknowledgments section?	Yes	Acknowledgments

Design

Study protocol	Information included in the manuscript?	In which section is the information available? (Reagents and Tools Table, Materials and Methods, Figures, Data Availability Section)
If study protocol has been **pre-registered, provide DOI in the manuscript**. For clinical trials, provide the trial registration number **OR** cite DOI.	Not Applicable	
Report the **clinical trial registration number** (at ClinicalTrials.gov or equivalent), where applicable.	Not Applicable	

Laboratory protocol	Information included in the manuscript?	In which section is the information available? (Reagents and Tools Table, Materials and Methods, Figures, Data Availability Section)
Provide DOI OR other citation details if **external detailed step-by-step protocols** are available.	Not Applicable	

Experimental study design and statistics	Information included in the manuscript?	In which section is the information available? (Reagents and Tools Table, Materials and Methods, Figures, Data Availability Section)
Include a statement about **sample size** estimate even if no statistical methods were used.	Yes	Material and methods
Were any steps taken to minimize the effects of subjective bias when allocating animals/samples to treatment (e.g. **randomization procedure**)? If yes, have they been described?	Not Applicable	
Include a statement about **blinding** even if no blinding was done.	Not Applicable	
Describe **inclusion/exclusion criteria** if samples or animals were excluded from the analysis. Were the criteria pre-established?<br><br>If sample or data points were omitted from analysis, report if this was due to attrition or intentional exclusion and provide justification.	Not Applicable	
For every figure, are **statistical tests** justified as appropriate? Do the data meet the assumptions of the tests (e.g., normal distribution)? Describe any methods used to assess it. Is there an estimate of variation within each group of data? Is the variance similar between the groups that are being statistically compared?	Yes	Material and methods

Sample definition and in-laboratory replication	Information included in the manuscript?	In which section is the information available? (Reagents and Tools Table, Materials and Methods, Figures, Data Availability Section)
In the figure legends: state number of times the experiment was **replicated** in laboratory.	Yes	Figure Legends
In the figure legends: define whether data describe **technical or biological replicates**.	Yes	Figure Legends

Ethics

Ethics	Information included in the manuscript?	In which section is the information available? (Reagents and Tools Table, Materials and Methods, Figures, Data Availability Section)
Studies involving **human participants**: State details of **authority granting ethics approval** (IRB or equivalent committee(s), provide reference number for approval.	Not Applicable	
Studies involving **human participants**: Include a statement confirming that **informed consent** was obtained from all subjects and that the experiments conformed to the principles set out in the WMA Declaration of Helsinki and the Department of Health and Human Services Belmont Report.	Not Applicable	
Studies involving **human participants:** For publication of **patient photos**, include a statement confirming that consent to publish was obtained.	Not Applicable	
Studies involving experimental **animals**: State details of **authority granting ethics approval** (IRB or equivalent committee(s), provide reference number for approval. Include a statement of compliance with ethical regulations.	Yes	Material and methods
Studies involving **specimen and field samples:** State if relevant **permits** obtained, provide details of authority approving study; if none were required, explain why.	Not Applicable	

Dual Use Research of Concern (DURC)	Information included in the manuscript?	In which section is the information available? (Reagents and Tools Table, Materials and Methods, Figures, Data Availability Section)
Could your study fall under dual use research restrictions? Please check biosecurity documents and list of **select agents and toxins** (CDC): https://www.selectagents.gov/sat/list.htm	Not Applicable	
If you used a select agent, is the security level of the lab appropriate and reported in the manuscript?	Not Applicable	
If a study is subject to dual use research of concern regulations, is the name of the **authority granting approval and reference number** for the regulatory approval provided in the manuscript?	Not Applicable	

Reporting

The MDAR framework recommends adoption of discipline-specific guidelines, established and endorsed through community initiatives. Journals have their own policy about requiring specific guidelines and recommendations to complement MDAR.

Adherence to community standards	Information included in the manuscript?	In which section is the information available? (Reagents and Tools Table, Materials and Methods, Figures, Data Availability Section)
State if relevant guidelines or checklists (e.g., **ICMJE, MIBBI, ARRIVE, PRISMA**) have been followed or provided.	Not Applicable	
For **tumor marker prognostic studies,** we recommend that you follow the **REMARK** reporting guidelines (see link list at top right). See author guidelines, under 'Reporting Guidelines'. Please confirm you have followed these guidelines.	Not Applicable	
For **phase II and III randomized controlled trials**, please refer to the **CONSORT** flow diagram (see link list at top right) and submit the CONSORT checklist (see link list at top right) with your submission. See author guidelines, under 'Reporting Guidelines'. Please confirm you have submitted this list.	Not Applicable	

Data Availability

Data availability	Information included in the manuscript?	In which section is the information available? (Reagents and Tools Table, Materials and Methods, Figures, Data Availability Section)
Have **primary datasets** been deposited according to the journal's guidelines (see 'Data Deposition' section) and the respective accession numbers provided in the Data Availability Section?	Yes	Data Availabity section
Were **human clinical and genomic datasets** deposited in a public access-controlled repository in accordance to ethical obligations to the patients and to the applicable consent agreement?	Not Applicable	
Are **computational models** that are central and integral to a study available without restrictions in a machine-readable form? Were the relevant accession numbers or links provided?	Not Applicable	
If publicly available data were reused, provide the respective **data citations in the reference list**.	Not Applicable	
