# Supplementary material for: Whole genome duplication through mitotic slippage causes nuclear instability
Source: EMBO J. 2026 Sep 19;45(20):7103–35. doi: 10.1038/s44318-026-00912-y (PMC13672370; doi:10.1038/s44318-026-00912-y)
Supplement: Supplementary file 5 [file 44318_2026_912_MOESM11_ESM.zip › Expanded view Movie 9/Movie EV9.docx]

Movie EV9: Megakaryocytes become polyploid by mitotic slippage.

Time lapse videos Megakaryocytes expressing mCherry-H2B (in magenta) and performing mitotic slippage. Images were acquired every 10 minutes.
