# Supplementary material for: Whole genome duplication through mitotic slippage causes nuclear instability
Source: EMBO J. 2026 Sep 19;45(20):7103–35. doi: 10.1038/s44318-026-00912-y (PMC13672370; doi:10.1038/s44318-026-00912-y)
Supplement: Supplementary file 14 [file 44318_2026_912_MOESM3_ESM.zip › Expanded view Movie 1/Movie EV1.docx]

Movie EV1: Mitotic slippage generates tetraploid cells with nuclear deformations.

Time lapse videos of RPE-1 cells expressing mCherry-H2B (in magenta) and performing canonical mitosis. Images were acquired every 5 minutes.
