# Supplementary material for: Whole genome duplication through mitotic slippage causes nuclear instability
Source: EMBO J. 2026 Sep 19;45(20):7103–35. doi: 10.1038/s44318-026-00912-y (PMC13672370; doi:10.1038/s44318-026-00912-y)
Supplement: Supplementary file 17 [file 44318_2026_912_MOESM6_ESM.zip › Expanded view Movie 4/Movie EV4.docx]

Movie EV4: Microtubules accumulate around nuclear deformations upon mitotic slippage.

Time lapse videos of RPE-1 cells expressing mCherry-H2B (in magenta) performing mitotic slippage. Actin was labeled using SPY FAST Actin (in blue). Images were acquired every 3 minutes.
