# Supplementary material for: Whole genome duplication through mitotic slippage causes nuclear instability
Source: EMBO J. 2026 Sep 19;45(20):7103–35. doi: 10.1038/s44318-026-00912-y (PMC13672370; doi:10.1038/s44318-026-00912-y)
Supplement: Supplementary file 20 [file 44318_2026_912_MOESM9_ESM.zip › Expanded view Movie 7/Movie EV7.docx]

Movie EV7: Microtubule depolymerization prevents nuclear deformations upon mitotic slippage induced by CCNB1 depletion.

Time lapse videos of RPE-1 CCNB1 cells expressing mCherry-H2B (in magenta) and performing mitotic slippage in the absence of Nocodazole. Microtubules were labeled using SPY Tubulin (in Cyan). Images were acquired every 3 minutes.
